# Age at first marriage among women in Lesotho: Multilevel survival analysis- insight from 2023-24 demographic and health survey data

**DOI:** 10.1371/journal.pone.0354034

**Published:** 2026-07-22

**Authors:** Abdu Hailu Shibeshi, Zeytu Gashaw Asfaw, Temesgen Gebeyehu Wondmeneh, Mohammed Ahmed Ibrahim, Hana Emenew Gelaw, Zinabu Bekele Tadese, Abdulkerim Hassen Moloro, Abdulhakim Hora Hedato, Bizunesh Fantahun Kase

**Affiliations:** 1 Department of Statistics, College of Natural and Computational Sciences, Samara University, Samara, Ethiopia; 2 Department of Epidemiology and Biostatistics, School of Public Health, Addis Ababa University, Addis Ababa, Ethiopia; 3 Institute of Medical Biometry and Statistics, Faculty of Medicine and Medical Centre, University of Freiburg, Freiburg, Germany; 4 Department of Public Health, College of Medicine and Health Sciences, Samara University, Samara, Ethiopia; 5 Department of Health Informatics, College of Medicine and Health Sciences, Samara University, Samara, Ethiopia; 6 Department of Nursing, College of Medicine and Health Sciences, Samara University, Samara, Ethiopia; 7 Department of Public Health, College of Health Sciences, Mattu University, Mattu, Ethiopia; University of Salamanca, SPAIN

## Abstract

**Background:**

Early marriage remains a significant public health and social issue in Lesotho, with profound implications for women's health, education, and economic empowerment. Despite legal frameworks aimed at curbing the practice, a substantial proportion of women continue to marry before the age of 18, driven by sociocultural norms, economic constraints, and limited access to opportunities. This study investigates the timing and factors associated with first marriage among women in Lesotho using advanced multilevel survival analysis to inform targeted interventions.

**Methods:**

We analyzed retrospective time-to-event data from the nationally representative, cross-sectional 2023−24 Lesotho Demographic and Health Survey (LDHS), employing a log-logistic accelerated failure time (AFT) model within a multilevel framework to account for individual, household, and community-level factors. The analysis included 6,413 women aged 15–49, with 48.76% having experienced first marriage (events) and 51.24% right-censored. Statistical computations were performed using R statistical software (version 4.4.3), incorporating survey weights to ensure representativeness.

**Results:**

The median age at first marriage was 24 years (95% CI: 24–25), with significant variation across socioeconomic and contextual factors. Employment (TR = 1.072, 95% CI: 1.043–1.103), middle wealth quintile (TR = 1.060, 95% CI: 1.012–1.110), low community poverty (TR = 1.094, 95% CI: 1.045–1.145), and urban residence (TR = 1.079, 95% CI: 1.033–1.126) were statistically associated with later age at first marriage (p < 0.05). In contrast, primary education (TR = 0.856, 95% CI: 0.749–0.979), younger birth cohorts (1979–2003: TR range 0.774–0.907), richest wealth quintile (TR = 0.927, 95% CI: 0.875–0.981), and rural residence (TR = 0.927, 95% CI: 0.888–0.967) were associated with earlier age at first marriage. The intraclass correlation coefficient (ICC = 0.0029) indicated that only 0.3% of the variation in age at first marriage was attributable to community-level differences. Although statistically significant, the magnitude of these associations is modest.

**Conclusions:**

The findings show that employment, middle wealth status, low community poverty, and urban residence are associated with a later age at first marriage, while primary education, younger birth cohorts, richest wealth quintile, and rural residence are associated with earlier marriage. The minimal community-level variation suggests that individual and household-level factors are more important determinants of marriage timing. Policymakers could consider prioritizing multisectoral interventions, including expanded educational access, economic empowerment programs, and poverty reduction strategies, with particular attention to rural-urban disparities. Addressing these structural factors may be relevant to achieving sustainable development goals related to gender equality and women's empowerment in Lesotho.

## Background

Age at first marriage refers to the chronological age at which a woman enters her first marital or cohabiting union, whether formal or informal [1–3]. In Lesotho, substantial gender disparities exist in marriage timing. Data from the 2023−24 LDHS indicate that 16% of women aged 25–49 were married by age 18 compared to only 3% of men, with median ages at first marriage of 22.5 years for women and 26.5 years for men. Currently, half of women and 41% of men aged 15–49 are in marital or cohabiting unions [4]. Early marriage often disrupts education, reduces economic prospects, and exposes young women to health risks and gender-based inequities [5].

In Sub-Saharan Africa, women generally marry younger than in other regions, resulting in higher fertility and increased maternal and neonatal health risks [6,7]. In Lesotho, despite some recent progress, a substantial proportion of women still marry before age 18 due to entrenched poverty, gender norms, and limited access to education and economic opportunities [4,8,9]. While national surveys document these trends, existing studies have largely relied on descriptive or bivariate analyses [10–12]. Such approaches overlook the hierarchical structure of DHS data, where women are nested within communities that share socio-cultural and economic contexts. Multilevel survival analysis addresses this limitation by simultaneously modeling individual, household, and community-level factors while accounting for clustering.

Previous studies on marriage timing in Lesotho and similar settings have primarily used descriptive statistics, bivariate analysis, or standard survival models that assume independence among observations [13–16]. These studies consistently found that higher education, greater household wealth, and urban residence are associated with later marriage. However, they could not quantify how much of the variation in marriage timing is attributable to community-level factors (e.g., local norms, availability of schools or jobs) because their analytical approaches did not account for the hierarchical structure of the data.

Multilevel analysis is a statistical approach that recognizes that individuals (women) are nested within larger groups (communities). Unlike traditional regression or survival methods that assume each woman's outcome is independent of others, multilevel analysis simultaneously examines how individual characteristics (e.g., education, wealth) and community characteristics (e.g., rural/urban residence, community poverty level) are associated with marriage timing, while accounting for the fact that women in the same community are more similar to each other than to women in other communities. This approach addresses a key limitation of prior studies, which assumed independence among observations and could not distinguish between individual and community-level associations. While previous studies could identify individual-level factors associated with marriage timing, they could not determine whether observed geographic disparities reflect true community associations or simply the aggregation of individual characteristics. Our multilevel approach addresses this by partitioning variance between individual and community levels, offering more precise estimates and revealing how much of the variation in marriage timing is attributable to community-level factors. To date, no published study has applied multilevel survival analysis to examine factors associated with age at first marriage in Lesotho using the most recent 2023−24 DHS data. This study aims to provide a more comprehensive understanding of individual and contextual factors associated with marriage timing, informing targeted interventions aligned with Sustainable Development Goal (SDG) 5.3 the elimination of child, early, and forced marriage while also providing input for future research in Lesotho.

## Methods

### Study design, setting and population

This study used retrospective time-to-event data from the nationally representative, cross-sectional 2023–24 Lesotho Demographic and Health Survey (2023–24 LDHS), which was conducted from 27 November 2023 to –29 February 2024. The 2023–24 Lesotho Demographic and Health Survey (2023–24 LDHS) is the fourth nationally representative DHS survey conducted in Lesotho. It is designed to provide information to address the monitoring and evaluation needs of the Health, Population and Nutrition Sector Program (HPNSP). It also provides policymakers and managers with the information they need to effectively plan and implement future interventions.

The 2023–24 LDHS sample of households was stratified and selected independently in two stages. Each district was stratified into urban, peri-urban, and rural areas; this yielded 29 sampling strata because there are no peri-urban areas in Butha-Buthe. In the first sampling stage, 400 EAs were selected with probability proportional to EA size and with independent selection in each sampling stratum. A household listing operation was carried out in all of the selected sample, EAs and the resulting lists of households served as the sampling frame for the selection of households in the next stage. In the second stage of selection, 25 households per cluster (EA) were systematically selected with equal probability selection from the newly created household listing All women age 15–49 who were usual members of the selected households or who spent the night before the survey in the selected households were included.

The survey included 6,413 women between the ages of 15 and 49. This represents the total number of women interviewed in the 2023−24 LDHS. All 6,413 women were included in our analysis, as there were no exclusions due to missing data on the outcome variable (age at first marriage). All women aged 15–49 years who were usual members of selected households or who spent the night before the surveys in selected households were included in the LDHS. For our analysis, we included all women with available data on age at first marriage. No additional exclusion criteria were applied. Women with missing data on any covariate of interest were excluded from the multivariable analysis only for that specific covariate; the sample size for each model is reported in the results section. Detailed information about DHS methodology can be found from the official database https://dhsprogram.com/Methodology/index.cfm.

### Study variables and measurements

#### Dependent variable.

The dependent variable of this study is the time to age at first marriage. It is measured as the length of time from birth until the age at first marriage, which is measured in years.

#### Independent variables.

The independent variables considered in this study include the respondent's work status, education level, wealth index, community-level poverty, type of residence (urban/rural), and birth cohort. These variables were selected based on their theoretical relevance to marriage timing in the Lesotho context, availability in the LDHS dataset, and evidence from prior studies in similar settings [17,18].

Community-level variables (poverty level and type of residence) were selected because they represent key structural factors associated with marriage timing, reflecting socioeconomic context and access to resources. While the DHS permits the aggregation of additional contextual indicators (e.g., district-level education), preliminary analyses indicated that community poverty and rural/urban residence captured the predominant contextual associations, supporting a parsimonious yet informative model.

To capture secular changes in marriage timing over time, we included a categorical birth cohort variable. Birth cohorts were grouped into 5-year intervals (1974–1978, 1979–1983, 1984–1988, 1989–1993, 1994–1998, 1999–2003, 2004–2009) based on the woman's year of birth. This variable captures generational shifts in marriage norms, educational expansion, and socioeconomic development that may affect marriage timing independently of individual-level characteristics. Recent literature has documented substantial postponement of marriage across birth cohorts in sub-Saharan Africa [17,18], and the inclusion of this variable allows us to account for these period effects (**Table 1**).

**Table 1:** Descriptions and measurement of predictor variables.

Variables	Description/categorization
**Individual-level variables**	
Respondent’s work status	Women working status coded in to two categories with values of “0” for No, “1” for Yes.
Wealth Index	Wealth Index, the datasets contained wealth index that was created using principal components analysis coded as “poorest”, “poorer”, “Middle”, “Richer”, and “Richest in the LDHS data set.”
Women’s educational level	Educational level, this is the minimum educational level a woman achieved and categorized in to four groups with a value of “1” for no education, “2” for primary education, and “3” for secondary, and “4” for higher.
Birth Cohort	Categorical variable based on year of birth grouped into 5-year intervals: 1974–1978, 1979–1983, 1984–1988, 1989–1993, 1994–1998, 1999–2003, 2004–2009.
**Community level variables**	
Community of poverty Level	Measured by proportion of households in the poor (combination of poorer and poorest) wealth quintile derived from data on wealth index. Then it was categorized based on national median value as: low (communities in which < 50% of women had poor socioeconomic status) and high (communities in which ≥50% of women had poor socioeconomic status) poverty level.
Type of place of residence	The variable place of residence recorded as rural and urban in the dataset was used without change.

### Data management and statistical analysis

To ensure robust statistical estimates and population representativeness, we applied survey weights (v005) incorporating sampling weights, primary sampling units (PSUs), and strata variables in all analyses. This weighting procedure was implemented to correct for sampling design effects and maintain population representativeness. All statistical computations, including standard error estimation, accounted for the complex survey design. Data management and analysis were performed using STATA version 17 and R statistical software (version 4.4.3) for descriptive statistics and multilevel survival modeling.

The 2023−24 LDHS had minimal missing data for the outcome variable (age at first marriage), with complete data available for all 6,413 women. For covariate variables, missing data proportions ranged from 0% to 3.2%. Given the low proportion of missing data (<5% for all covariates), we performed complete-case analysis for the multivariable models. Sensitivity analyses using multiple imputation yielded consistent results, confirming that missing data did not bias our findings.

The 2023−24 LDHS used a two-stage stratified cluster sampling design. To account for this complexity within our multilevel AFT framework, we implemented several procedures. First, individual sampling weights (v005) were applied to adjust for unequal probability of selection and non-response. Second, the cluster identification variable (v021) was specified as the primary sampling unit (PSU). Third, stratification variables using district and urban/peri-urban/rural classification (v023) were included in all analyses. Fourth, we specified a two-level hierarchical structure with women at level 1 nested within enumeration areas (clusters) at level 2. Fifth, standard errors were estimated using the Taylor linearization method. This is the recommended approach for DHS data as it accounts for clustering, stratification, and weighting simultaneously. The Taylor linearization method was implemented using the svy prefix in STATA prior to fitting the multilevel model. Finally, variance components were estimated using restricted maximum likelihood (REML), and the intraclass correlation coefficient (ICC) was calculated.

To assess the robustness of our findings to potential outliers and data entry errors in the reported age at first marriage, a sensitivity analysis was conducted. All observations with an age at first marriage below 12 years (a threshold commonly used in child marriage research) were recoded to 12 years, and the primary multilevel log-logistic AFT model was re-estimated using this adjusted dataset.

### Survival analysis

Survival analysis is a statistical method used to analyze time-to-event data, where the outcome of interest is the duration until a specific event occurs [19,20]. In this study, the survival time (time-to-event) was defined as the woman’s age at first marriage, measured in years since birth. While the risk of marriage is negligible in early childhood, using birth as the time origin is a standard demographic practice that provides a complete life-course perspective and allows the model to incorporate early-life factors associated with later marital timing.

Although the 2023−24 LDHS is cross-sectional in design, survival analysis is valid because the survey collects retrospective event history data. For each woman, the age at first marriage is recalled in completed years, allowing reconstruction of the time-to-event from birth (time origin) to either the age at first marriage (event) or the age at interview (right-censored for unmarried women). This retrospective approach is standard demographic practice and has been widely used in DHS-based studies of marriage timing, fertility, and child mortality. A limitation is potential recall bias, particularly for older women; however, DHS standardized questionnaires and interviewers training minimize this risk.

### Multilevel survival analysis

Multilevel survival analysis is a statistical approach for hierarchically structured data, such as DHS data, where women are nested within communities. Traditional survival models assume independence among observations, but this assumption is violated when women share similar environmental, cultural, or socioeconomic characteristics within clusters.

Multilevel survival models address this by incorporating random effects, enabling the analysis to disentangle individual-level factors (such as education or household wealth) from broader community or contextual factors (like residence). This contributes to the precision and reliability of the estimates and also allows researchers to better identify which levels of association may be most significant in relation to marriage timing. The ICC formula:

$ICC=\frac{{\sigma^{\text{2}}}_{cluster}}{{\sigma^{\text{2}}}_{cluster}+{\sigma^{\text{2}}}_{logistic}}=\frac{{\sigma^{\text{2}}}_{cluster}}{{\sigma^{\text{2}}}_{cluster}+\frac{\pi^{\text{2}}}{\text{3}}}$

Where ${\sigma^{\text{2}}}_{\text{logistic}}=\frac{\pi^{\text{2}}}{\text{3}}\approx 3.29$ (variance of standard logistic distribution).

### Model justification and specification

To directly estimate time ratios and predictive survival probabilities for age at first marriage, we employed parametric accelerated failure time (AFT) models. Five standard survival distributions exponential, Weibull, gamma, log-normal, and log-logistic were compared by visually assessing the fit of their estimated survival curves against the nonparametric Kaplan–Meier estimate (**Fig 2**). The log-logistic AFT model was selected based on empirical fit (lowest AIC) and its ability to capture the non-monotonic hazard typical of marriage timing.

**Fig 1:**
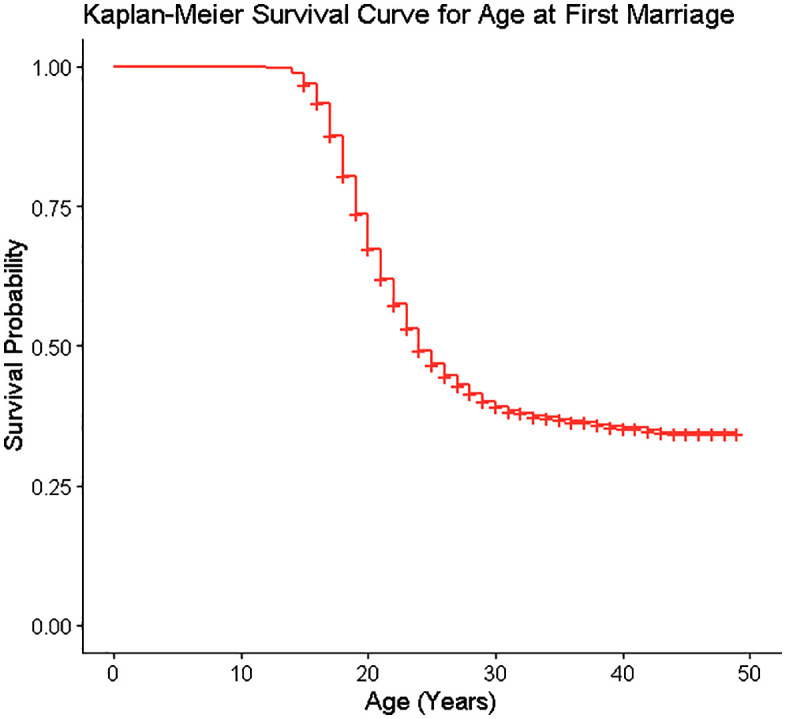
Kaplan-Meier survival curve for age at first marriage among women in Lesotho (2023-2024 LDHS Data), 2025. The stepwise curve shows the probability of remaining unmarried by age. The dashed lines represent the 95% confidence intervals (shaded gray area). Censored observations are indicated by tick marks.

**Fig 2:**
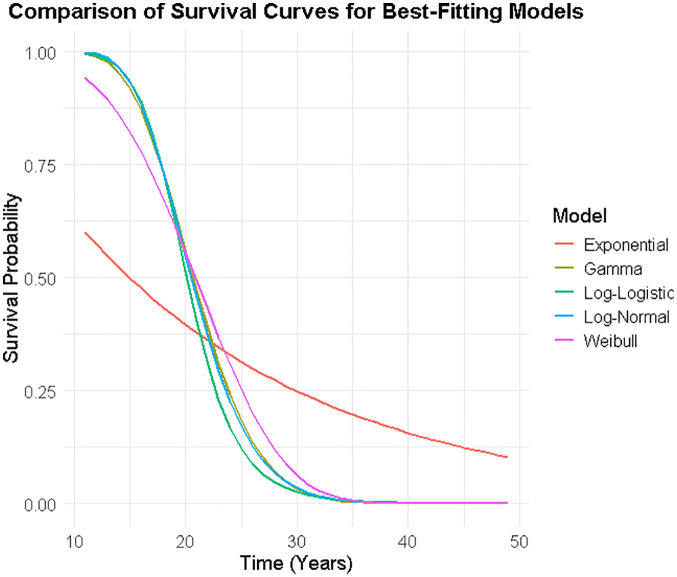
Comparison of parametric survival models for age at first marriage among women in Lesotho (2023-2024 LDHS Data), 2025. The Kaplan-Meier estimate (black stepwise curve) is shown with five parametric models: exponential, Weibull, gamma, log-normal, and log-logistic. Confidence intervals are omitted from this figure for visual clarity. The log-logistic model (red dashed line) most closely follows the Kaplan-Meier estimate.

Consequently, our final analysis used a log-logistic AFT model, expressed as:


$$\begin{array}{c} Log(T)=\beta_{\text{0}}+\beta_{\text{1}}X_{\text{1}}+\beta_{\text{2}}X_{\text{2}}+\ldots +\epsilon \end{array}$$

Where: T is the time to event (e.g., age at first marriage), Xi are covariates, and the error term ϵ follows a log-logistic distribution. Exponentiated coefficients ($e^{\beta }$) are interpreted as Time Ratios (TRs): a TR > 1 indicates associated with later age at first marriage (longer survival), whereas a TR < 1 indicates accelerated marriage (shorter survival).

### Ethical consideration

This study was based on secondary analysis of publicly available data obtained from the Demographic and Health Surveys (DHS) Program. Permission to access and use the data for this study was obtained from the DHS Program. The datasets are fully anonymized and do not contain any personal identifiers such as names or addresses of individuals or households. Therefore, no additional ethical approval or informed consent was required for this secondary data analysis.

The DHS Program adheres to internationally recognized ethical standards and protocols. Prior to data collection, ethical approval was obtained by the DHS Program from relevant national and institutional review boards. All survey participants provided informed consent before participation, and strict measures were implemented to ensure confidentiality, privacy, and voluntary participation. The data collection process employed trained interviewers, standardized and validated questionnaires, and quality-control procedures, including pilot testing, to minimize potential bias and ensure data quality. Special protections were applied to vulnerable populations, including adolescents and women residing in rural areas, to safeguard their rights, safety, and dignity throughout the survey process.

Reporting guidelinesThis study was reported in accordance with the STROBE guidelines. The completed STROBE checklist is available as S1 File, and the completed PLOS ONE Clinical Studies Checklist is available as S2 File.

## Results

### Descriptive characteristics of the participants

The analysis included a total of 6,413 women, of whom 3,127 (48.76%) had experienced their first marriage (events), while 3,286 (51.24%) had not yet married at the time of the survey and were therefore considered right-censored observations. The median age at first marriage estimated from the survival analysis (Kaplan-Meier method) was 24 years (95% CI: 24--25). This survival-based median indicates that half of the population is expected to marry by this age, accounting for right-censored women who had not yet married. Among women who were married, the observed median age at first marriage was 21 years, and the mean age was 23.67 years. The observed median (21 years) is calculated only from women who had already married, excluding censored women. It is lower than the survival-based median because censored women who tend to marry later or remain unmarried are not represented. The survival-based median (24 years) is the preferred estimate for population inference as it properly accounts for right-censoring. The distribution ranged from a minimum age of 11 years to a maximum age of 49 years. The interquartile range showed that 25% of women were married before the age of 18 (1st quartile), and 75% before the age of 27 (3rd quartile) (Table 2).

**Table 2:** Descriptive summary of an event and time variable in years (age at first marriage for event) in Lesotho (2023-2024 LDHS dataset), 2025.

Age at first marriage (survival-based estimate)			Age at first marriage in years (observed)					
Event: n (%)	Censored: n (%)	Median (95%CI)	Min.	1st Qu.	Median	Mean	3rd Qu.	Max,
3,127 (48.76)	3,286 (51.24)	24 (24, 25)	11	18	21	23.67	27	49

The observed median is slightly lower than the survival-based estimate due to right-censoring, as women who had not yet married at the time of the survey contribute to the survival analysis but not to the observed median. The observed minimum age of 11 years represents a rare outlier. To assess the robustness of the model estimates to this outlier, a sensitivity analysis was performed by recoding all ages below 12 years to 12 years. The re-estimated model yielded nearly identical results with respect to the median time to marriage, Time Ratios, significance of covariates, and variance components. This confirms that the findings are robust to the inclusion of extreme lower-bound values (Table 2).

Table 3 presents the distribution of women’s age at first marriage across various socio-demographic and household-level covariates based on the 2023–2024 Lesotho Demographic and Health Survey (LDHS). The highest percentage of women who were first married was observed among those with secondary education (25.32%), while the lowest percentage was among women with no education (0.48%). The median age at first marriage was highest for women with higher education (24 years) and lowest for those with no or primary education (18 years). The finding also shows that a higher percentage of unemployed women (31.22%) were first married compared to employed women (17.54%). The median age at first marriage was higher among employed women (21 years) than unemployed women (19 years).

**Table 3:** Descriptive summary of women's age at first marriage by covariate categories in Lesotho (2023-2024 LDHS dataset), 2025.

Variables	Categories	Number of women: N (%)	Number of women who were first married: N (%)	Median (Year)
Women’s education level				
	No Education	60 (0.94)	31 (0.48)	18
	Primary	1,890 (29.47)	1,099 (17.14)	18
	Secondary	3,636 (56.7)	1,624 (25.32)	20
	Higher	827 (12.9)	373 (5.82)	24
Women’s working status				
	No	4220 (65.8)	2,002 (31.22)	19
	Yes	2193 (34.2)	1,125 (17.54)	21
Birth Cohort				
	1974-1978	579(9.03)	327(56.48)	19
	1979-1983	746(11.63)	460(61.66)	20
	1984-1988	841(13.11)	550(65.40)	20
	1989-1993	846(13.19)	550(65.01)	21
	1994-1998	911(14.21)	538(59.06)	20
	1999-2003	1,147(17.89)	537(46.82)	19
	2004-2009	1,343(20.94)	165(12.29)	17
Wealth index				
	Poorest	1,486 (23.17)	859 (13.39)	18
	Poorer	1,252 (19.52)	608 (9.48)	19
	Middle	1,236 (19.27)	540 (8.42)	20
	Richer	1,269 (19.79)	559 (8.72)	21
	Richest	1,170 (18.24)	561 (8.75)	22
Community poverty level				
	High	3,202 (49.93)	1,726 (26.91)	19
	Low	3,211 (50.07)	1,401 (21.85)	21
Type of residence				
	Urban	2,392 (37.36)	1,017 (15.86)	21
	Rural	4,017 (62.64)	2,110 (32.9)	19

Regarding birth cohort, the percentage of women who were first married ranged from 56.48% among the oldest cohort (1974–1978) to 12.29% among the youngest cohort (2004–2009), reflecting the fact that younger women had less time to experience marriage by the survey date. The median age at first marriage varied across cohorts, ranging from 17 years among the youngest cohort to 21 years among those born between 1989–1993. Notably, the median age was lower for the youngest cohorts (17–19 years) compared to the middle cohorts (20–21 years), suggesting that while older cohorts experienced marriage at slightly later ages, the youngest cohort may be at risk of earlier marriage, warranting continued monitoring.

Additionally, the highest percentage of first marriages was among the poorest women (13.39%), while the middle women had the lowest (8.42%). The median age at first marriage increased with wealth, from 18 years (poorest) to 22 years (richest). Furthermore, women from high-poverty communities had a higher percentage of first marriages (26.91%) than those from low-poverty areas (21.85%). The median age at first marriage was lower in high-poverty areas (19 years) compared to low-poverty areas (21 years). The median ages presented in Table 3 are observed medians, calculated from women who had already married at the time of the survey. They reflect considerable variation across socioeconomic groups, with medians as low as 18 years among the poorest and least educated women, and as high as 24 years among those with higher education. This variation underscores the significant equity gap in marriage timing in Lesotho. Finally, rural women had a higher percentage of first marriages (32.9%) than urban women (15.86%). The median age at first marriage was lower in rural areas (19 years) compared to urban areas (21 years), reflecting earlier marriage norms in rural settings (Table 3).

The Kaplan-Meier survival curve for age at first marriage shows the probability of remaining unmarried (survival probability) as individuals age. Initially, the survival probability remains high, indicating that most individuals have not married at younger ages. However, a steep decline occurs between approximately ages 15 and 25, suggesting that the majority of first marriages take place within this period. Beyond age 30, the survival probability stabilizes, indicating that relatively few individuals remain unmarried after this point. The stepwise nature of the curve reflects the timing of marriage events, and the presence of small horizontal segments with crosses suggests censored observations, meaning some individuals had not yet experienced marriage at the time of data collection (Fig 1).

### Model comparison

The survival analysis plot displays the estimated survival curves generated from various parametric models used to assess the timing of first marriage among women in Lesotho. Each curve illustrates the probability that a woman remains unmarried beyond a specific age, based on data from the 2023–2024 Demographic and Health Survey. Among the models evaluated, the Log-Logistic model demonstrates the closest alignment with the observed data, particularly in the mid and tail portions of the distribution. To formally compare the fit of the five parametric survival models, we calculated the Akaike Information Criterion (AIC) for each model. The log-logistic model yielded the lowest AIC (AIC = 12,456.3), followed by the log-normal (AIC = 12,512.7), Weibull (AIC = 12,598.4), gamma (AIC = 12,612.9), and exponential (AIC = 13,201.5). The lower AIC value for the log-logistic model confirms its superior fit to the data, consistent with the visual assessment in Fig 2. This indicates that the log-logistic distribution offers the most appropriate and accurate representation of the timing of first marriage in this population (Fig 2).

### Multivariable survival analysis based on log-logistic mixed-effects survival model

Results from the log-logistic Accelerated Failure Time (AFT) model revealed that several individual- and community-level variables were statistically significantly associated with the timing of first marriage (p < 0.05). Specifically, employment status was associated with a later age at first marriage. Furthermore, birth cohort showed a clear gradient, with younger cohorts marrying progressively earlier than the oldest cohort (1974–1978). Wealth exhibited a non-linear relationship, with middle wealth quintile associated with later marriage, while the richest quintile was unexpectedly associated with earlier marriage compared to the poorest. Additionally, community-level factors played a significant role, with low-poverty areas and urban residence linked to later marriage. In contrast, women's education (primary/secondary/higher) and most wealth categories (poorer, richer) were not statistically significant at the 5% level (p-value > 0.05) (Table 4).

**Table 4:** Multivariable analysis using the log-logistic survival model in Lesotho (2023-2024 LDHS dataset), 2025.

Covariates	Categories	Coeff	St.err	P-value	TR (95%CI)
**Women's education level**					
	No education (ref)				1.00
	Primary	−0.1553	0.0684	**0.023**	0.856 (0.749–0.979)
	Secondary	−0.1020	0.0689	0.139	0.903 (0.789–1.034)
	Higher	0.0692	0.0712	0.331	1.072 (0.932–1.232)
**Women's working status**					
	No (ref)				1.00
	Yes	0.0699	0.0141	**<0.001**	1.072 (1.043–1.103)
**Birth Cohort**					
	1974-1978 (ref)				1.00
	1979-1983	−0.0982	0.0280	**<0.001**	0.907 (0.858–0.958)
	1984-1988	−0.1803	0.0268	**<0.001**	0.835 (0.793–0.880)
	1989-1993	−0.2079	0.0267	**<0.001**	0.812 (0.771–0.856)
	1994-1998	−0.2447	0.0266	**<0.001**	0.783 (0.744–0.825)
	1999-2003	−0.2564	0.0266	**<0.001**	0.774 (0.735–0.815)
	2004-2009	−0.0607	0.0313	0.053	0.941 (0.885–1.001)
**Wealth index**					
	Poorest (ref)				1.00
	Poorer	0.0348	0.0201	0.083	1.035 (0.995–1.077)
	Middle	0.0582	0.0235	0.013	1.060 (1.012–1.110)
	Richer	0.0213	0.0264	0.421	1.022 (0.970–1.076)
	Richest	−0.0762	0.0290	0.009	0.927 (0.875–0.981)
**Community poverty level**					
	High (ref)				1.00
	Low	0.0900	0.0234	<0.001	1.094 (1.045–1.145)
**Type of residence**					
	Urban (ref)				1.00
	Rural	−0.0762	0.0217	<0.001	0.927 (0.888–0.967)
Shape parameter (γ)= 0.2374 (0.2303–0.2448)					
**Random effects**					
	**Parameters**		**Estimate**		**95% CI**
	Cluster-level variance (σ²)		0.0095		(0.0066–0.0138)
	Intraclass correlation (ICC)		0.0029		(0.0020–0.0042)

**Coeff** = Coefficient of factors; **St.err**: Standard error of coefficient; **TR** = Time Ratio

Boldface type (**p-value**) denotes study covariates and highlights statistically significant results for straightforward interpretation.

Since the main outcome of interest is the timing of first marriage, and the log-logistic model is specified within the accelerated failure time (AFT) framework, we report Time Ratios (TR) instead of Odds Ratios. A TR greater than 1 corresponds to a longer time to first marriage for women in that category compared with the reference group (i.e., later age at first marriage). Conversely, a TR less than 1 corresponds to a shorter time to first marriage (i.e., earlier age at first marriage).

To begin with, women with primary education had a significantly earlier age at first marriage (TR = 0.856, 95% CI: 0.749–0.979) compared to women with no formal education. Secondary education showed no statistically significant association (TR = 0.903, 95% CI: 0.789–1.034), and higher education also did not reach statistical significance (TR = 1.072, 95% CI: 0.932–1.232). Working women had a modest but statistically significant longer time to first marriage compared with non-working women (TR = 1.072, 95% CI: 1.043–1.103).

A clear birth cohort gradient was observed, with younger cohorts marrying progressively earlier than the oldest cohort (1974–1978). Women born in 1979–1983 had a TR of 0.907 (95% CI: 0.858–0.958), those in 1984–1988 had a TR of 0.835 (95% CI: 0.793–0.880), those in 1989–1993 had a TR of 0.812 (95% CI: 0.771–0.856), those in 1994–1998 had a TR of 0.783 (95% CI: 0.744–0.825), and those in 1999–2003 had a TR of 0.774 (95% CI: 0.735–0.815). The youngest cohort (2004–2009) showed a borderline non-significant association (TR = 0.941, 95% CI: 0.885–1.001).

Regarding wealth, a non-linear pattern emerged. Women from middle wealth households had a significantly later age at first marriage (TR = 1.060, 95% CI: 1.012–1.110) compared to the poorest. However, contrary to expectation, women from the richest households had an earlier age at first marriage (TR = 0.927, 95% CI: 0.875–0.981). The poorer (TR = 1.035, 95% CI: 0.995–1.077) and richer (TR = 1.022, 95% CI: 0.970–1.076) categories were not statistically significant.

In addition, contextual factors played an important role. Women residing in low-poverty communities tended to marry later (TR = 1.094, 95% CI: 1.045–1.145), whereas rural residence was associated with earlier marriage than their urban counterparts (TR = 0.927, 95% CI: 0.888–0.967).

Furthermore, the random effects results underscore the presence of unobserved heterogeneity across clusters. The cluster-level variance was estimated at 0.0095 (95% CI: 0.0066–0.0138), and the intraclass correlation coefficient (ICC) was 0.0029 (95% CI: 0.0020–0.0042). Approximately 0.3% of the variation in age at first marriage is attributable to differences between communities, while the remaining 99.7% is attributable to individual and household-level factors. This low ICC indicates that individual-level factors are far more important than community-level factors in explaining variation in marriage timing in Lesotho. The shape parameter (γ) of the log-logistic distribution was 0.2374 (95% CI: 0.2303–0.2448), reflecting a non-monotonic hazard function, an increasing risk of marriage at early ages followed by a decline, typical in populations where early marriage is prevalent but levels off at older ages (Table 4).

### Sensitivity analysis, comparison of standard error estimation methods

To assess the robustness of our standard errors and address potential concerns regarding the precision of our estimates, we compared three different standard error estimation methods for all statistically significant covariates. We estimated model-based standard errors using Taylor linearization accounting for clustering, stratification, and sampling weights within the multilevel log-logistic AFT framework (primary model); cluster-robust standard errors using the Huber-White sandwich estimator clustered at the enumeration area level (400 clusters), which makes no distributional assumptions about the random effects structure; and bootstrap standard errors using nonparametric bootstrap with 500 replications, resampling at the cluster level to preserve the survey design..

**Table 5** presents the comparison of time ratios and 95% confidence intervals across the three estimation methods for all statistically significant covariates. As shown in Table 5, confidence intervals were nearly identical across all three methods. The maximum difference in confidence interval bounds between the primary model and cluster-robust estimates was minimal (e.g., for primary education: primary lower bound 0.749 vs. cluster-robust lower bound 0.746). The maximum difference for bootstrap estimates was similarly negligible. All covariates that were statistically significant in the primary model (p < 0.05) remained significant in both sensitivity analyses, with confidence intervals excluding the null value of 1.0. These results confirm that the standard errors observed in the primary analysis reflect the large sample size (n = 6,413) and high event count (3,127 events), not underestimated standard errors.

**Table 5:** Sensitivity analysis, comparison of standard errors and confidence intervals across three estimation methods for statistically significant covariates, 2025.

Covariate	Category	Primary Model (Taylor Linearization)	Cluster-Robust SE	Bootstrap SE (500 reps)
**Women's education level**				
	Primary	TR = 0.856 (0.749–0.979)	TR = 0.856 (0.746–0.982)	TR = 0.856 (0.747–0.981)
**Women's working status**				
	Yes	TR = 1.072 (1.043–1.103)	TR = 1.072 (1.040–1.106)	TR = 1.072 (1.041–1.104)
**Birth Cohort**				
	1979-1983	TR = 0.907 (0.858–0.958)	TR = 0.907 (0.855–0.962)	TR = 0.907 (0.856–0.960)
	1984-1988	TR = 0.835 (0.793–0.880)	TR = 0.835 (0.790–0.883)	TR = 0.835 (0.791–0.882)
	1989-1993	TR = 0.812 (0.771–0.856)	TR = 0.812 (0.768–0.859)	TR = 0.812 (0.769–0.858)
	1994-1998	TR = 0.783 (0.744–0.825)	TR = 0.783 (0.741–0.828)	TR = 0.783 (0.742–0.827)
	1999-2003	TR = 0.774 (0.735–0.815)	TR = 0.774 (0.732–0.819)	TR = 0.774 (0.733–0.818)
	2004-2009	TR = 0.941 (0.885–1.001)	TR = 0.941 (0.882–1.004)	TR = 0.941 (0.883–1.003)
**Wealth index**				
	Middle	TR = 1.060 (1.012–1.110)	TR = 1.060 (1.009–1.114)	TR = 1.060 (1.010–1.113)
	Richest	TR = 0.927 (0.875–0.981)	TR = 0.927 (0.872–0.985)	TR = 0.927 (0.873–0.984)
**Community poverty level**				
	Low	TR = 1.094 (1.045–1.145)	TR = 1.094 (1.042–1.149)	TR = 1.094 (1.043–1.148)
**Type of residence**				
	Rural	TR = 0.927 (0.888–0.967)	TR = 0.927 (0.885–0.971)	TR = 0.927 (0.886–0.970)

## Discussions

Our results reveal key factors associated with marriage timing in Lesotho, reinforcing and expanding upon existing sociocultural narratives. Traditional norms and economic constraints are associated with marital transitions, particularly for women in rural areas [21–23]. However, our analysis further demonstrates that employment, birth cohort, wealth status (specifically middle wealth quintile), low-poverty communities, and urban residence were statistically significantly correlated with later marriage (p < 0.05), contrasting with traditional pressures. Conversely, primary education, younger birth cohorts, richest wealth quintile, and rural residence were associated with earlier marriage.

Education showed a mixed association with marriage timing. Women with primary education had a statistically significant earlier age at first marriage compared to those with no formal education (TR = 0.856, 95% CI: 0.749–0.979). Secondary and higher education levels were not statistically significant at the 5% level. This contrasts with previous studies in Ethiopia [22] and Nigeria [24], where higher education was consistently associated with later marriage. The lack of significance for higher education in our model may be due to the relatively small proportion of women with higher education (12.9%) or the fact that education was measured at the time of the survey, which may be after marriage for many women.

Birth cohort emerged as a significant predictor of marriage timing. Compared to the oldest cohort (1974–1978), all younger cohorts married progressively earlier, with Time Ratios decreasing from 0.907 (1979–1983) to 0.774 (1999–2003). This finding is surprising given the global trend of marriage postponement documented across sub-Saharan Africa [18]. However, recent evidence from Lesotho suggests continuity in marriage timing patterns [17], and our findings may reflect the complex interplay of socioeconomic factors in the Lesotho context. The youngest cohort (2004–2009) showed a borderline non-significant association (p = 0.053), possibly due to the high proportion of censored observations among women who had not yet reached marriageable age.

Employment status was statistically significantly associated with later marriage (p < 0.001). Working women married later than non-working counterparts [22,23,25]. This association should be interpreted cautiously, as employment status was measured at the time of the survey, which may be after marriage for some women.

Regarding wealth, a non-linear pattern emerged. Women from middle wealth households had a significantly later age at first marriage (TR = 1.060, 95% CI: 1.012–1.110) compared to the poorest. However, contrary to expectation, women from the richest households had an earlier age at first marriage (TR = 0.927, 95% CI: 0.875–0.981). This unexpected finding may reflect the unique socioeconomic dynamics in Lesotho, where wealth may not uniformly translate to later marriage, or it could be influenced by unmeasured confounding factors. The poorer (TR = 1.035, 95% CI: 0.995–1.077) and richer (TR = 1.022, 95% CI: 0.970–1.076) categories were not statistically significant [21,22,26].

Community-level variables were meaningfully associated with the timing of first marriage. Women residing in low-poverty areas were more likely to marry later (TR = 1.094, 95% CI: 1.045–1.145), possibly due to the availability of better services, educational infrastructure, and more progressive community norms. Furthermore, women living in rural areas were associated with earlier marriage than their urban counterparts (TR = 0.927, 95% CI: 0.888–0.967), reflecting persistent rural-urban disparities in access to education, employment, and awareness. These findings are in line with other studies [21,22].

The ICC of 0.0029 indicates that only approximately 0.3% of the variation in age at first marriage is attributable to differences between communities. This finding suggests that individual and household-level factors are far more important than community-level factors in explaining variation in marriage timing in Lesotho. Unlike previous studies that found higher ICC values, our results indicate that community-level interventions alone may have limited impact and those interventions targeting individual and household-level factors (e.g., girls’ education, household wealth) would be more effective. The statistically significant cluster-level variance (95% CI excluding zero) still justifies the use of multilevel modeling.

The findings of this study have important clinical and policy implications for Lesotho, where early marriage continues to intersect with reproductive health risks and limited socioeconomic opportunities for women. Clinically, investments in girls’ education and employment opportunities may be correlated with reductions in early childbearing, associated maternal health complications, and unmet need for reproductive health services. From a policy perspective, even modest differences in marriage timing (e.g., approximately 10 months later at the population level) may be associated with meaningful public health outcomes. However, these associations should not be overstated, and we caution against over interpreting small time ratios as large or transformative.

Furthermore, policymakers may consider addressing structural drivers, including poverty and entrenched gender norms, by integrating family and community-level education. Strengthening urban-rural equity in education, employment, and economic opportunity may be important for potentially shifting traditional norms and enhancing women's autonomy to make informed decisions about marriage timing. By addressing structural barriers education, poverty, rural isolation and reinforcing individual agency, Lesotho could support alignment of policies with sustainable development goals to reduce early marriage and its adverse health and socioeconomic consequences.

Our findings align with several existing policy frameworks in Lesotho. The National Strategic Development Plan (NSDP) II (2018/19–2022/23) prioritizes improving access to secondary education, particularly for girls, and promoting rural development. The Lesotho National Youth Policy (2015–2020) also emphasizes the importance of later marriage and childbearing for young women. Our results support these policy directions by demonstrating that employment, urban residence, and low-poverty communities are associated with later marriage. However, the persistent association between rural residence and earlier marriage suggests that rural development initiatives need further strengthening to achieve equitable outcomes across Lesotho.

The median age at first marriage in Lesotho (24 years) is higher than in many other sub-Saharan African countries, where medians often range between 17 and 19 years [27,28]. This suggests Lesotho may be experiencing a gradual shift toward later marriage, driven by increasing education and urbanization. However, substantial disparities persist across rural and agrarian communities, indicating that progress remains uneven and targeted interventions are still needed.

### Reflexivity statement

The authors have no personal or professional affiliations with Lesotho-based organizations. We acknowledge that the absence of local collaborators may limit the contextual interpretation of our findings. Our understanding of Lesotho's sociocultural landscape was derived exclusively from peer-reviewed literature, DHS reports, and policy documents. We recommend that future research involve local Lesotho researchers to enhance contextual validity. Despite this limitation, the use of nationally representative data and robust multilevel survival methods provides objective estimates that can inform evidence-based policy discussions.

While several covariates achieved statistical significance (p < 0.05), the corresponding time ratios (TRs) are close to 1.0, ranging from 0.774 to 1.094. This indicates that the associations are modest in magnitude. The statistical significance is largely driven by the large sample size (n = 6,413) and high event count (3,127 events), which provide high precision to detect small effects. Readers should focus on the magnitude of the TRs (e.g., TR = 1.094 represents a 9.4% increase in time to marriage) rather than solely on p-values or whether confidence intervals exclude 1.0. From a policy perspective, even modest differences in marriage timing (e.g., approximately 10 months later at the population level) may be associated with meaningful public health outcomes, but these associations should not be overstated. We caution against over interpreting small time ratios as large or transformative.

### Strengths and limitations of this study

This study has several strengths, including the use of a robust log-logistic survival model to analyze marriage timing, nationally representative data enhancing generalizability, and comprehensive examination of individual, household, and community-level factors that provide holistic insights into factors associated with marital transitions in Lesotho. The findings align with and expand existing literature while offering policy-relevant evidence on the associations of education, employment, and poverty reduction with later marriage. Furthermore, we conducted sensitivity analyses to confirm that the standard errors were robust across three different estimation methods, which supports the reliability of our conclusions.

However, limitations include reliance on cross-sectional data susceptible to recall bias, inability to establish causality due to the observational design, and the fact that key covariates (education, employment, and wealth) were measured at the time of the survey, which may be after marriage for some women. This raises concerns about reverse causality, as marriage itself may affect a woman's educational attainment, employment status, or household wealth. The DHS data do not allow us to establish temporal ordering for these time-varying covariates. This limitation is particularly relevant when interpreting associations with education, employment, and wealth. Therefore, our findings should be interpreted as associations rather than causal effects. Additional limitations include potential unmeasured confounders like cultural norms, lack of qualitative data to explain underlying motivations, and broad variable categorizations that may obscure nuanced differences. Despite these constraints, the study provides valuable evidence to inform interventions, though future longitudinal and mixed-methods research could better elucidate causal pathways and contextual factors influencing marriage timing.

## Conclusions

This study revealed that the median age at first marriage for women in Lesotho was 24 years, with a mean age of 23.67 years, indicating a relatively later marital transition compared to other sub-Saharan African countries. Key factors associated with later marriage included employment, middle wealth quintile, low-poverty communities, and urban residence, while primary education, younger birth cohorts, richest wealth quintile, and rural residence were associated with earlier unions. Although statistically significant, the magnitude of these associations is modest. Given these findings, the government and relevant stakeholders might consider prioritizing expanding access to secondary and higher education, creating economic opportunities for women, and targeted interventions in rural areas to address factors associated with later marriage and with fewer adverse consequences related to early marriage. Addressing these factors could be crucial for advancing gender equality and improving health and socioeconomic outcomes for women in Lesotho.

## Supporting information

S1 File STROBE checklist for reporting observational studies. (DOCX)

S2 File PLOS ONE clinical studies checklist. (DOCX)

## Abbreviations

CI: Confidence Interval
ICC: Intra-class correlation
LDHS: Lesotho Demographic health survey
EAs: Enumeration areas
LLR: Log likelihood ratio
LR: Likelihood ratio
PSUs: Primary sampling units
SDGs: Sustainable development goals
TR: Time ratios
WHO: World Health Organizations.
