# Supplementary material for: Age at first marriage among women in Lesotho: Multilevel survival analysis- insight from 2023-24 demographic and health survey data
Source: PLoS One. 2026 Jul 22;21(7):e0354034. doi: 10.1371/journal.pone.0354034 (PMC13390838; doi:10.1371/journal.pone.0354034)
Supplement: Attachment — Submitted filename: PONE-D-25-41860_Lesotho_2.pdf [file pone.0354034.s003.pdf]

PLOS ONE

Time to age at first marriage among women in Lesotho: Multilevel survival analysis (insights from recent 2023-24 demographic and health survey data)

--Manuscript Draft--

**Manuscript Number:**	PONE-D-25-41860
**Article Type:**	Research Article
**Full Title:**	Time to age at first marriage among women in Lesotho: Multilevel survival analysis (insights from recent 2023-24 demographic and health survey data)
**Short Title:**	Time to age at first marriage among women in Lesotho
**Corresponding Author:**	Abdu Hailu Shibeshi, MSc<br>Samara University<br>Semera, Afar ETHIOPIA
**Keywords:**	Age at first marriage; Lesotho; multilevel survival analysis; Demographic and Health Survey
**Abstract:**	Background<br><br>Early marriage remains a significant public health and social issue in Lesotho, with profound implications for women's health, education, and economic empowerment. Despite legal frameworks aimed at curbing the practice, a substantial proportion of women continue to marry before the age of 18, driven by sociocultural norms, economic constraints, and limited access to opportunities. This study investigates the timing and determinants of first marriage among women in Lesotho using advanced multilevel survival analysis to inform targeted interventions.<br><br>Methods<br><br>We conducted a population-based cross-sectional design from the 2023–24 Lesotho Demographic and Health Survey (LDHS), employing a log-logistic accelerated failure time (AFT) model within a multilevel framework to account for individual, household, and community-level factors. The analysis included 6,413 women aged 15–49, with 48.76% having experienced first marriage (events) and 51.24% right-censored. Statistical computations were performed using R statistical software (version 4.4.3), incorporating survey weights to ensure representativeness.<br><br>Results<br><br>The median age at first marriage was 24 years (95% CI: 24–25), with significant variation across socioeconomic and contextual factors. Higher education (TR = 1.112, 95% CI: 1.032–1.197), employment (TR = 1.033, 95% CI: 1.018–1.049), media exposure (TR = 1.019, 95% CI: 1.002–1.036), and wealth (richest: TR = 1.038, 95% CI: 1.008–1.070) delayed marriage, while agricultural household occupation (TR = 0.968, 95% CI: 0.946–0.991) and rural residence (TR = 0.977, 95% CI: 0.960–0.995) accelerated it. Cluster-level variance (ICC = 0.1461) indicated significant community-level heterogeneity.<br><br>Conclusions<br><br>The findings underscore the protective role of education, economic empowerment, and urban residence against early marriage, while highlighting the persistent influence of rural livelihoods and intergenerational norms. Policymakers should prioritize multisectoral interventions, including expanded educational access, media campaigns, and poverty reduction, to delay marriage and improve women’s health and autonomy. Addressing structural disparities between rural and urban areas is critical for achieving sustainable development goals in Lesotho.
**Order of Authors:**	Abdu Hailu Shibeshi, MSc, MPH
	Zeytu Gashaw Asfaw, PhD (Associate Professor)

Powered by Editorial Manager® and ProduXion Manager® from Aries Systems Corporation

	Temesgen Gebeyehu Wondmeneh, MPH
	Mohammed Ahmed Ibrahim, PhD
	Hana Emenew Gelaw, MSc
	Zinabu Bekele Tadese, MSc
	Abdulkerim Hassen Moloro, MSc
	Abdulhakim Hora Hedato, MPH
	Bizunesh Fantahun Kase, MPH
**Additional Information:**	
**Question**	**Response**
**Financial Disclosure**<br><br>Enter a financial disclosure statement that describes the sources of funding for the work included in this submission. Review the submission guidelines for detailed requirements. View published research articles from *PLOS ONE* for specific examples.<br><br>This statement is required for submission and **will appear in the published article** if the submission is accepted. Please make sure it is accurate.<br><br>**Funded studies**<br>Enter a statement with the following details:<br>• Initials of the authors who received each award<br>• Grant numbers awarded to each author<br>• The full name of each funder<br>• URL of each funder website<br>• Did the sponsors or funders play any role in the study design, data collection and analysis, decision to publish, or preparation of the manuscript?<br><br>Did you receive funding for this work?	The author(s) received no specific funding for this work.
**Competing Interests**<br><br>Use the instructions below to enter a competing interest statement for this submission. On behalf of all authors, disclose any competing interests that could be perceived to bias this work—acknowledging all financial support and any other relevant financial or non-	The authors have declared that no competing interests exist.

financial competing interests.<br><br>This statement is required for submission and **will appear in the published article** if the submission is accepted. Please make sure it is accurate and that any funding sources listed in your Funding Information later in the submission form are also declared in your Financial Disclosure statement.<br><br>View published research articles from *PLOS ONE* for specific examples.<br><br>**NO authors have competing interests**<br><br>Enter: *The authors have declared that no competing interests exist.*<br><br>**Authors with competing interests**<br><br>Enter competing interest details beginning with this statement:<br><br>*I have read the journal's policy and the authors of this manuscript have the following competing interests: [insert competing interests here]*<br><br>* typeset	
**Ethics Statement**<br><br>Enter an ethics statement for this submission. This statement is required if the study involved:<br><br>• Human participants<br>• Human specimens or tissue<br>• Vertebrate animals or cephalopods<br>• Vertebrate embryos or tissues<br>• Field research<br><br>Write "N/A" if the submission does not require an ethics statement.<br><br>General guidance is provided below.<br>Consult the submission guidelines for	N/A

Powered by Editorial Manager® and ProduXion Manager® from Aries Systems Corporation

detailed instructions. **Make sure that all information entered here is included in the Methods section of the manuscript.**<br><br>**Format for specific study types**<br><br>**Human Subject Research (involving human participants and/or tissue)**<br>• Give the name of the institutional review board or ethics committee that approved the study<br>• Include the approval number and/or a statement indicating approval of this research<br>• Indicate the form of consent obtained (written/oral) or the reason that consent was not obtained (e.g. the data were analyzed anonymously)<br><br>**Animal Research (involving vertebrate animals, embryos or tissues)**<br>• Provide the name of the Institutional Animal Care and Use Committee (IACUC) or other relevant ethics board that reviewed the study protocol, and indicate whether they approved this research or granted a formal waiver of ethical approval<br>• Include an approval number if one was obtained<br>• If the study involved *non-human primates*, add *additional details* about animal welfare and steps taken to ameliorate suffering<br>• If anesthesia, euthanasia, or any kind of animal sacrifice is part of the study, include briefly which substances and/or methods were applied<br><br>**Field Research**<br><br>Include the following details if this study involves the collection of plant, animal, or other materials from a natural setting:<br>• Field permit number<br>• Name of the institution or relevant body that granted permission	
**Data Availability**<br><br>Authors are required to make all data underlying the findings described fully available, without restriction, and from the time of publication. PLOS allows rare	Yes - all data are fully available without restriction

Powered by Editorial Manager® and ProduXion Manager® from Aries Systems Corporation

exceptions to address legal and ethical concerns. See the PLOS Data Policy and FAQ for detailed information.<br><br>A Data Availability Statement describing where the data can be found is required at submission. Your answers to this question constitute the Data Availability Statement and **will be published in the article**, if accepted.<br><br>**Important:** Stating 'data available on request from the author' is not sufficient. If your data are only available upon request, select 'No' for the first question and explain your exceptional situation in the text box.<br><br>Do the authors confirm that all data underlying the findings described in their manuscript are fully available without restriction?	
**Describe where the data may be found in full sentences. If you are copying our sample text, replace any instances of XXX with the appropriate details.**<br><br>• If the data are **held or will be held in a public repository**, include URLs, accession numbers or DOIs. If this information will only be available after acceptance, indicate this by ticking the box below. For example: *All XXX files are available from the XXX database (accession number(s) XXX, XXX.*).<br>• If the data are all contained **within the manuscript and/or Supporting Information files**, enter the following: *All relevant data are within the manuscript and its Supporting Information files.*<br>• If neither of these applies but you are able to provide **details of access elsewhere**, with or without limitations, please do so. For example:<br><br>*Data cannot be shared publicly because of [XXX]. Data are available from the XXX Institutional Data Access / Ethics Committee (contact via XXX) for researchers who meet the criteria for*	All relevant data are within the manuscript and its Supporting Information files.

access to confidential data.<br><br>*The data underlying the results presented in the study are available from (include the name of the third party and contact information or URL).*<br>• This text is appropriate if the data are owned by a third party and authors do not have permission to share the data.<br><br>* typeset	
Additional data availability information:	

Powered by Editorial Manager® and ProduXion Manager® from Aries Systems Corporation

Manuscript Click here to access/download;Manuscript;Manuscript.docx

Time to age at first marriage among women in Lesotho: Multilevel survival analysis (insights from recent 2023-24 demographic and health survey data)

Consider modifying the title: Age at first marriage among women in Lesotho: Multilevel survival analysis (insights from 2023-24 demographic and health survey data). Multilevel survival analysis took care of timing which is missing in the suggested title.

Abdu Hailu Shibeshi[1*], Zeytu Gashaw Asfaw[2], Temesgen Gebeyehu Wondmeneh[3], Mohammed Ahmed Ibrahim[3], Hana Emenew Gelaw[1], Zinabu Bekele Tadese[4], Abdulkerim Hassen Moloro[5], Abdulhakim Hora Hedato[3], Bizunesh Fantahun Kase[3]

Affiliations

[1]Department of Statistics, College of Natural and Computational Sciences, Samara University, Samara, Ethiopia

[2]Department of Epidemiology and Biostatistics, School of Public Health, Addis Ababa University, Addis Ababa, Ethiopia

[3]Department of Public Health, College of Medicine and Health Sciences, Samara University, Samara, Ethiopia

[4]Department of Health Informatics, College of Medicine and Health Sciences, Samara University, Samara, Ethiopia

[5]Department of Nursing, College of Medicine and Health Sciences, Samara University, Samara, Ethiopia

[*]Corresponding author

Name: Abdu Hailu Shibeshi

Email: abduhailu01@gmail.com

Abstract

Background: Early marriage remains a significant public health and social issue in Lesotho, with profound implications for women's health, education, and economic empowerment. Despite legal frameworks aimed at curbing the practice, a substantial proportion of women continue to marry before the age of 18, driven by sociocultural norms, economic constraints, and limited access to opportunities. This study investigates the timing and determinants of first marriage among women in Lesotho using advanced multilevel survival analysis to inform targeted interventions.

Methods: We conducted a population-based cross-sectional design from the 2023–24 Lesotho Demographic and Health Survey (LDHS), employing a log-logistic accelerated failure time (AFT) model within a multilevel framework to account for individual, household, and community-level factors. The analysis included 6,413 women aged 15–49, with 48.76% having experienced first marriage (events) and 51.24% right-censored. Statistical computations were performed using R statistical software (version 4.4.3), incorporating survey weights to ensure representativeness.

Results: The median age at first marriage was 24 years (95% CI: 24–25), with significant variation across socioeconomic and contextual factors. Higher education (TR = 1.112, 95% CI: 1.032–1.197), employment (TR = 1.033, 95% CI: 1.018–1.049), media exposure (TR = 1.019, 95% CI: 1.002–1.036), and wealth (richest: TR = 1.038, 95% CI: 1.008–1.070) delayed marriage, while agricultural household occupation (TR = 0.968, 95% CI: 0.946–0.991) and rural residence (TR = 0.977, 95% CI: 0.960–0.995) accelerated it. Cluster-level variance (ICC = 0.1461) indicated significant community-level heterogeneity.

Conclusions: The findings underscore the protective role of education, economic empowerment, and urban residence against early marriage, while highlighting the persistent influence of rural

livelihoods and intergenerational norms. Policymakers should prioritize multisectoral interventions, including expanded educational access, media campaigns, and poverty reduction, to delay marriage and improve women’s health and autonomy. Addressing structural disparities between rural and urban areas is critical for achieving sustainable development goals in Lesotho.

Keywords: Age at first marriage, Lesotho, multilevel survival analysis, Demographic and Health Survey

Background

Time to age at first marriage defined as the duration from birth until a woman enters her first legally or socially recognized marital union [1]. This demographic measure is closely related to age at first marriage, which specifically denotes the chronological age when a woman first enters such a partnership, regardless of whether it is formalized through legal processes or established through informal social recognition [2, 3]. In Lesotho, the age at first marriage is measured by the percentage of women aged 20–24 who were first married by the age of 18 [4, 5].

Suggest re-writing this sentence as: In Lesotho, indicator for age of first marriage is the percentage of 20-24-year-old women who were married at age 18.

Marriage patterns in Lesotho reveal stark gender disparities. Women tend to marry significantly earlier than men, 16% of women aged 25–49 were married by 18, compared to just 3% of men. The median age at first marriage is 22.5 years for women and 26.5 years for men. However, the trend reverses for sexual debut, with 57% of men and 43% of women reporting first sexual intercourse by age 18. Presently, 50% of women and 41% of men aged 15–49 are in marital or cohabiting unions [6].

Despite legal frameworks aimed at reducing early marriage, a considerable proportion of women in Lesotho continue to marry before 18, highlighting the limited effectiveness of current

interventions [6]. Early marriage often disrupts education, reduces economic prospects, and exposes young women to health risks and gender-based inequities. These patterns underscore the importance of investigating the timing and determinants of first marriage [7].

In Sub-Saharan Africa, women generally marry younger than in other regions, resulting in higher fertility and increased maternal and neonatal health risks [8, 9]. Lesotho stands out for its relatively high prevalence of early marriage, warranting focused attention [10]. Although recent trends suggest a rise in the median age at first marriage, potentially influenced by urbanization, education, and socioeconomic changes persistent challenges like poverty, gender norms, and limited access to opportunities continue to shape marriage behavior [11].

National survey data such as the Demographic and Health Surveys (DHS) have reported descriptive statistics and trends on age at first marriage, highlighting that a significant proportion of women still marry before the age of 18 [6]. However, a critical gap remains in the analytical depth and methodological rigor of existing studies. Most analyses to date rely heavily on basic descriptive statistics or bivariate relationships, which fail to capture the complex interplay of individual, household, and community-level factors that influence the timing of marriage. Moreover, current literature lacks any dedicated, empirical investigation into the factors associated with time to age at first marriage using advanced statistical approaches, particularly multilevel survival analysis in Lesotho [12-14]. This is a major limitation, given the hierarchical structure of DHS data, where individual women are nested within communities or clusters, which in turn may have shared socio-cultural, economic, or policy influences affecting marriage timing.

To date, no published study has applied multilevel survival analysis to examine the timing and associated factors of age at first marriage in Lesotho using the most recent 2023–2024 DHS data. This gap presents a compelling opportunity to contribute novel insights to the literature, support data-driven policy recommendations, and align with Sustainable Development Goal (SDG) 5.3 on eliminating early and forced marriage. By leveraging robust, multilevel modeling techniques, this study will provide a more comprehensive understanding of both individual-level characteristics and contextual-level influences, thereby informing targeted interventions aligned with Sustainable Development Goal 5.3 to eliminate child, early, and forced marriage, and it provides input for further study in Lesotho.

Methods

Study design, setting and population

This study used a population-based cross-sectional design and data from 2023–24 Lesotho Demographic and Health Survey (2023–24 LDHS), which was gathered from 27 November 2023 to 29 February 2024. The 2023–24 Lesotho Demographic and Health Survey (2023–24 LDHS) is the fourth nationally representative DHS survey conducted in Lesotho. It is designed to provide information to address the monitoring and evaluation needs of the Health, Population and Nutrition Sector Program (HPNSP). It also provides policymakers and managers with the information they need to effectively plan and implement future interventions.

The 2023–24 LDHS sample of households was stratified and selected independently in two stages. Each district was stratified into urban, peri-urban, and rural areas; this yielded 29 sampling strata because there are no peri-urban areas in Butha-Buthe. In the first sampling stage, 400 EAs were

selected with probability proportional to EA size and with independent selection in each sampling stratum. A household listing operation was carried out in all of the selected sample EAs, and the resulting lists of households served as the sampling frame for the selection of households in the next stage. In the second stage of selection, 25 households per cluster (EA) were systematically selected with equal probability selection from the newly created household listing All women age 15–49 who were usual members of the selected households or who spent the night before the survey in the selected households were included. The survey included 6,413 women between the ages of 15 and 49. Detailed information about DHS methodology can be found from the official database https://dhsprogram.com/Methodology/index.cfm.

Study variables and measurements

Dependent variable

The dependent variable of this study is the time to age at first marriage. It is measured as the length of time from birth until the age at first marriage, which is measured in years.

Independent variables

The independent variables considered in this study are the respondent's work status, religion, type of residence, head education level, women education level, head occupation, access to media, and wealth index (Table 1). All independent variables were extracted based on reviewed literatures [15].

Table 1 Descriptions and measurement of predictor variables

Variables	Description/categorization
**Individual-level variables**	
Respondent's work status	Women working status coded in to two categories with values of "0" for No, "1" for Yes.
Wealth Index	Wealth Index, the datasets contained wealth index that was created using principal components analysis coded as "poorest", "poorer", "Middle", "Richer", and "Richest in the LDHS data set."
Head occupation	Occupation, recoded in seven categories as no working, professional, clerical, sales and services, skilled manual, unskilled manual, agriculture
Media exposure	Media exposure, A composite variable obtained by combining whether a respondent reads newspaper/magazine, listen to radio, and watch television with a value of "0" if women were not exposed to at least one of the three medias, and "1" if a woman has access/exposure to at least one of the three medias.
Women's educational level	Educational level, this is the minimum educational level a woman achieved and categorized in to four groups with a value of "1" for no education, "2" for primary education, and "3" for secondary, and "4" for higher.
Head education	Educational level, this is the minimum educational level a woman achieved and categorized in to four groups with a value of "1" for no education, "2" for primary education, and "3" for secondary, and "4" for higher
**Community level variables**	
Community of poverty Level	Measured by proportion of households in the poor (combination of poorer and poorest) wealth quintile derived from data on wealth index. Then it was categorized based on national median value as: low (communities in which < 50% of women had poor socioeconomic status) and high (communities in which ≥50% of women had poor socioeconomic status) poverty level.
Type of place of residence	The variable place of residence recorded as rural and urban in the dataset was used without change.

Data management and statistical analysis

To ensure robust statistical estimates, we applied survey weights incorporating sampling weights, primary sampling units (PSUs), and strata variables. This weighting procedure was implemented to correct for sampling design effects and maintain population representativeness in all analyses. All statistical computations, including standard error estimation, accounted for the complex survey design. Data management and analysis were performed using STATA version 17 and R statistical software (version 4.4.3) for descriptive statistics and multilevel survival modeling.

Survival Analysis

Survival analysis is a statistical method used to analyze time-to-event data, where the outcome of interest is the duration until a specific event occurs [16, 17].

Multilevel survival analysis

Multilevel survival analysis is a powerful statistical approach that offers significant advantages in studying time-to-event data, particularly in contexts where data are hierarchically structured as is the case with Demographic and Health Survey (DHS) data. In the analysis of time to age at first marriage, this method is especially important because it accounts for both the timing of the event (i.e., marriage) and the nested nature of the data, where individuals are grouped within households or communities. Traditional survival models often assume independence among observations, but this assumption is violated when women share similar environmental, cultural, or socioeconomic characteristics within clusters.

Multilevel survival models address this by incorporating random effects, enabling the analysis to disentangle individual-level factors (such as education or household wealth) from broader community or contextual influences (like residence). This not only improves the precision and reliability of the estimates but also allows researchers to identify which levels of influence are most significant in shaping marriage timing. Therefore, multilevel survival analysis provides a more nuanced and realistic understanding of the determinants of early marriage, making it a critical tool for informing targeted interventions and policy development in Lesotho and similar settings.

The ICC formula:

$$ICC = \frac{{\sigma^2}_{cluster}}{{\sigma^2}_{cluster} + {\sigma^2}_{logistic}} = \frac{{\sigma^2}_{cluster}}{{\sigma^2}_{cluster} + \frac{\pi^2}{3}}$$

Where $\sigma^2{}_{\text{logistic}} = \frac{\pi^2}{3} \approx \mathbf{3.29}$ (variance of standard logistic distribution).

Model justification

The Log-Logistic model was selected as the most appropriate for analyzing time to age at first marriage due to its ability to accommodate a non-monotonic hazard function. In the context of marriage timing, the risk of entering a first union typically increases during adolescence and early adulthood, peaks in the mid-20s, and then declines with age. The Log-Logistic distribution effectively captures this pattern, making it well-suited for modeling such life-course events. Additionally, its flexibility in handling long-tailed distributions aligns with observed variability in marriage timing, where some women marry much later or remain unmarried within the observation window. Given its strong theoretical and empirical suitability for demographic and social transitions, the Log-Logistic model provides both a robust and interpretable framework for this analysis.

Parametric survival models

Parametric survival models constitute a family of analytical approaches that require specification of an underlying probability distribution for survival times. Among the most frequently utilized distributions in survival analysis are the exponential, Weibull, gamma, log-normal, and log-logistic distributions. Each distribution offers unique characteristics that make it suitable for modeling different patterns of survival data [18].

Exponential distribution

The exponential distribution is characterized by its memoryless property, indicating a constant hazard rate over time. For a survival time T following an exponential distribution with rate parameter $\lambda > 0$, the probability density function (PDF) is given by:

$f(t) = \lambda e^{-\lambda t}$, where $t \geq 0, \lambda \geq 0$

The corresponding survival function and hazard function are respectively: $S(t) = e^{-\lambda t}$ and $h(t) = \lambda$. where λ represents the constant hazard rate

The Weibull distribution

The Weibull distribution is unique in its ability to accommodate both proportional hazards and accelerated failure time model parameterizations. Its flexible hazard function can model monotonically increasing ($\gamma > 1$), decreasing ($\gamma < 1$), or constant ($\gamma = 1$) hazard rates over time [19]. For survival time T following a Weibull distribution with scale parameter $\lambda > 0$ and shape parameter $\gamma > 0$, the key functions are defined as follows:

Probability density function (PDF): $f(t) = \lambda\gamma t^{\gamma-1}e^{-\lambda t^{\gamma}} \quad \lambda,\gamma > 0$

Survival function: $S(t) = e^{-\lambda t^{\gamma}}$ and

Hazard function: $h(t) = \lambda\gamma t^{\gamma-1}$

Where,

- λ (scale parameter) determines the distribution's spread
- γ (shape parameter) governs the hazard function's behavior.

Log-logistic distribution

The log-logistic distribution is particularly suitable for modeling survival data with non-monotonic hazard rates, where the hazard function initially increases before eventually decreasing (unimodal shape). For a survival time T following a log-logistic distribution with scale parameter θ and shape parameter $k > 0$, the key functions are defined as:

Probability density function (PDF): $f(t) = \frac{e^{\theta}\, kt^{k-1}}{(1+e^{\theta}t^{k})^{2}} \quad for\ 0 \leq t < \infty,\ k > 0$

Survival function: $S(\,t) = \ (1 + e^{\theta} t^{k)}\ ^{-1}$and

Hazard function: $h(\,t)\ = \frac{e^{\theta}\ kt^{k-1}}{1+e^{\theta}t^{k}}$

Where,

- $\theta\ \in\ \mathbb{R}$ is the scale parameter governing the distribution's location.
- $k\ >\ 0$ is the shape parameter determining the hazard function's behavior.
- When $k\ \leq\ 1$, the hazard function decreases monotonically.
- When $k\ >\ 1$, the hazard function peaks at $t\ =\ [(k-1)/e\hat{}\theta]\hat{}(1/k)$

Log-normal distribution

The lognormal distribution is particularly suited for modeling positively valued random variables. A survival time T follows a lognormal distribution with parameters μ and σ if $log(T)$ is normally distributed with mean μ and standard deviation σ. The key functions characterizing this distribution are.

Probability Density Function (PDF): $f(t) = \frac{1}{\delta\sqrt{2\pi}} t^{-1} e^{-\frac{1}{2}\frac{(-log(t)-\mu)^2}{\delta^2}}\ \ where\ 0 \leq t < \infty,\ \delta\ > 0$

Survival Function: $S(\,t) = \ 1 - \ \Phi\left(\frac{logt-\ \mu}{\sigma}\right)$ and

Hazard Function: $h(\,t)\ = \frac{\phi\left(\frac{logt}{\sigma}\right)}{1-\ \Phi\left(\frac{logt}{\sigma}\right)\sigma t}$

Where,

- $\phi(\cdot)$ denotes the standard normal probability density function
- $\Phi(\cdot)$ represents the standard normal cumulative distribution function
- $\mu \in R$ determines the scale of the distribution
- $\sigma > 0$ controls the shape and spread of the distribution

The gamma distribution

The gamma distribution is characterized by two positive parameters: shape (ρ) and rate (λ). For a survival time T following this distribution, the key functions are defined as follows:

Probability Density Function (PDF): $f(t) = \frac{\lambda^{\rho} t^{\rho-1} e^{-\lambda t}}{\Gamma(\rho)}\ for\ \ 0 \leq t < \infty, \quad \lambda > 0, and\ \ \rho > 0$

Where,

- Mean: $\mu\ =\ \rho/\lambda$
- Variance: $\sigma^2\ =\ \rho/\lambda^2$

Survival Function: $S(t) =\ 1 - \frac{\gamma(\rho,\lambda t)}{\Gamma(\rho)}$

Hazard Function: $h(t) = {f(t)}/{S(t)}$, where $\gamma(\rho, \lambda t)$is known as the incomplete gamma function, given by; $\gamma(\rho, \lambda t)$= $\int_0^{\lambda t} u^{\rho-1}\, e^{-u} du$

Accelerated failure time model (AFT)

Accelerated failure time model is a type of parametric survival model that directly models the log of survival time as a linear function of covariates [20]. The general AFT form:

$$Log(T) = \beta_0 + \beta_1 X_1 + \beta_2 X_2 + \cdots + \epsilon$$

Where:

- T is the time to event (e.g., age at first marriage),
- Xi are covariates,
- ϵ is an error term from a specific distribution (e.g., log-logistic, Weibull, log-normal, etc.).

Coefficients in AFT models and Odds Ratio (OR)

Definition: $TR = e^{\beta}$

- Exponentiated coefficients $e^{\beta}$are Time Ratios (TRs).

- A Time Ratio $>$ 1 means the event happens later (delayed).
- A Time Ratio $<$ 1 means the event happens sooner (accelerated).

Definition: $OR(t) = \dfrac{\frac{S\left(\frac{t}{X_1}\right)}{1-S\left(\frac{t}{X_1}\right)}}{\frac{S\left(\frac{t}{X_0}\right)}{1-S\left(\frac{t}{X_0}\right)}} = e^{\beta.shape}$

- $OR > 1$: Higher odds of survival at any time t.
- $OR < 1$: Lower odds of survival at any time t.

Ethical consideration

Since this study was a secondary data analysis of publicly available survey data from the measure DHS program**,** it did not require ethical approval or participant consent. The data was obtained from http://www.dhsprogram.com with permission for this study. The datasets do not contain any names or addresses of individuals or households.

Results

Descriptive characteristics of the participants

The analysis included a total of 6,413 women, of whom 3,127 (48.76%) had experienced their first marriage (events), while 3,286 (51.24%) had not yet married at the time of the survey and were therefore considered right-censored observations. The median time to first marriage was 24 years (95% CI: 24–25), indicating that half of the population married by this age. Among those who were married, the observed mean age at first marriage was 23.67 years. The distribution ranged from a minimum age of 11 years to a maximum age of 49 years. The interquartile range showed that 25% of women were married before the age of 18 (1st quartile), and 75% before the age of 27 (3rd quartile). The observed median age at marriage (50th percentile) was 21 years,

which is slightly lower than the survival-based median, possibly due to the impact of right censoring (Table 2).

Table 2: Descriptive summary of an event and time variable in years (age at first marriage for event) in Lesotho (2023-2024 LDHS dataset), 2025

**Time to age at first marriage**			**Age at first marriage (in years)**					
Event: n (%)	Censored: n (%)	Median (95%CI)	Min.	1st Qu.	Median	Mean	3rd Qu.	Max,
3,127 (48.76)	3,286 (51.24)	24 (24, 25)	11	18	21	23.67	27	49

Table 3 presents the distribution of women's age at first marriage across various socio-demographic and household-level covariates based on the 2023–2024 Lesotho Demographic and Health Survey (LDHS). The highest percentage of women who were first married was observed among those with secondary education (25.32%), while the lowest percentage was among women with no education (0.48%). The median age at first marriage was highest for women with higher education (24 years) and lowest for those with no or primary education (18 years). The finding also shows that a higher percentage of unemployed women (31.22%) were first married compared to employed women (17.54%). The median age at first marriage was higher among employed women (21 years) than unemployed women (19 years).

Regards to the educational level of household's head, women from households where the head had primary education had the highest percentage of first marriages (20.51%), while those with a higher-educated head had the lowest (5.69%). The median age at first marriage was highest (23 years) when the household head had higher education and lowest (19 years) when the head had no or primary education. The results also indicate that the highest proportion of first marriages (14.42%) occurred among women from households where the head was engaged in unskilled

manual occupations, whereas the lowest proportion (0.8%) was observed in households headed by individuals in clerical occupations. The median age at first marriage was highest (22 years) for women with professional household heads and lowest (19 years) for those with agricultural or non-working heads.

Women exposed to mass media had a higher percentage of first marriages (33.17%) than those without exposure (15.59%). However, the median age at first marriage was higher (20 years) among women with media exposure compared to those without (18 years). Additionally, the highest percentage of first marriages was among the poorest women (13.39%), while the middle women had the lowest (8.42%). The median age at first marriage increased with wealth, from 18 years (poorest) to 22 years (richest).

Furthermore, women from high-poverty communities had a higher percentage of first marriages (26.91%) than those from low-poverty areas (21.85%). The median age at first marriage was lower in high-poverty areas (19 years) compared to low-poverty areas (21 years). Finally, rural women had a higher percentage of first marriages (32.9%) than urban women (15.86%). The median age at first marriage was lower in rural areas (19 years) compared to urban areas (21 years), reflecting earlier marriage norms in rural settings (Table 3).

Table 3: Descriptive summary of women's age at first marriage by covariate categories in Lesotho (2023-2024 LDHS dataset), 2025

Variables	Categories	Number of women: N (%)	Number of women who were first married: N (%)	Median (Year)
Women's education level	No Education	60 (0.94)	31 (0.48)	18
	Primary	1,890 (29.47)	1,099 (17.14)	18
	Secondary	3,636 (56.7)	1,624 (25.32)	20
	Higher	827 (12.9)	373 (5.82)	24

Women's working status	No	4220 (65.8)	2,002 (31.22)	19
	Yes	2193 (34.2)	1,125 (17.54)	21
Head education level (n=3,226)	No Education	438 (6.83)	415 (6.47)	19
	Primary	1,348 (21.02)	1,315 (20.51)	19
	Secondary	1,066 (16.62)	1,032 (16.09)	20
	Higher	374 (5.83)	365 (5.69)	23
Head occupation (n=3,226)	Did not work	920 (14.35)	892 (13.91)	19
	Professional	233 (3.63)	225 (3.51)	22
	Clerical	51 (0.8)	51 (0.8)	20
	Sales and services	220 (3.43)	211 (3.29)	20
	Skilled manual	510 (7.95)	494 (7.7)	20
	Unskilled manual	961 (14.99)	925 (14.42)	20
	Agriculture	331 (5.16)	329 (5.13)	19
Mass media exposure	No	1,902 (29.66)	1,000 (15.59)	18
	Yes	4,511 (70.34)	2,127 (33.17)	20
Wealth index	Poorest	1,486 (23.17)	859 (13.39)	18
	Poorer	1,252 (19.52)	608 (9.48)	19
	Middle	1,236 (19.27)	540 (8.42)	20
	Richer	1,269 (19.79)	559 (8.72)	21
	Richest	1,170 (18.24)	561 (8.75)	22
Community poverty level	High	3,202 (49.93)	1,726 (26.91)	19
	Low	3,211 (50.07)	1,401 (21.85)	21
Type of residence	Urban	2,392 (37.36)	1,017 (15.86)	21
	Rural	4,017 (62.64)	2,110 (32.9)	19

The Kaplan-Meier survival curve for age at first marriage shows the probability of remaining unmarried (survival probability) as individuals age. Initially, the survival probability remains high, indicating that most individuals have not married at younger ages. However, a steep decline occurs between approximately ages 15 and 25, suggesting that the majority of first marriages take place within this period. Beyond age 30, the survival probability stabilizes, indicating that relatively few individuals remain unmarried after this point. The stepwise nature of the curve reflects the timing of marriage events, and the presence of small horizontal segments with crosses suggests censored observations, meaning some individuals had not yet experienced marriage at the time of data collection (Fig 1).

Fig 1: Kaplan-Meier survival curve for age at first marriage among women in Lesotho (2023-2024 LDHS Data), 2025

Model comparison

The survival analysis plot displays the estimated survival curves generated from various parametric models used to assess the timing of first marriage among women in Lesotho. Each curve illustrates the probability that a woman remains unmarried beyond a specific age, based on data from the 2023–2024 Demographic and Health Survey. Among the models evaluated, the Log-Logistic model demonstrates the closest alignment with the observed data, particularly in the mid and tail portions of the distribution. This indicates that the log-logistic distribution offers the most appropriate and accurate representation of the timing of first marriage in this population (Fig 2).

Fig 2: Comparison of parametric survival models for age at first marriage among women in Lesotho (2023-2024 LDHS Data), 2025

Multivariable survival analysis based on log-logistic mixed-effects survival model

Results from the log-logistic Accelerated Failure Time (AFT) model revealed that several individual- and community-level variables were significantly associated with the timing of first marriage at the 5% level of significance ($p < 0.05$). Specifically, higher education, employment status, and media exposure all showed protective effects against early marriage. Furthermore, wealth demonstrated a dose-response relationship, with each increasing quintile (from poorer to richest) corresponding to greater marriage delays. At the household level, both head's primary education and agricultural occupation were associated with earlier marriage. Additionally, community characteristics emerged as important, with low-poverty areas and urban residence showing protective effects. In contrast, women's education (primary/secondary), head education

(secondary/higher), and most head occupation categories were not significant at the 5% level of significance (p-value > 0.05) (Table 4).

Since the main outcome of interest is the timing of first marriage, and the log-logistic model is specified within the accelerated failure time (AFT) framework, we report Time Ratios (TR) instead of Odds Ratios. A TR greater than 1 indicates a delayed transition to first marriage for women in that category compared to the reference group, implying they marry later. Conversely, a TR less than 1 suggests an earlier transition to marriage, meaning women in that group tend to marry sooner than their counterparts in the reference category.

To begin with, women who attained higher education experienced a significantly delayed age at first marriage (TR = 1.112, 95% CI: 1.032–1.197) compared to women with no formal education. Similarly, working women had a longer time to first marriage (TR = 1.033, 95% CI: 1.018–1.049) than non-working women. Consistently, exposure to mass media was associated with delayed marriage (TR = 1.019, 95% CI: 1.002–1.036).

Moreover, a clear wealth gradient was observed: women from all wealth categories above the poorest were more likely to delay marriage, including the poorer (TR = 1.023), middle (TR = 1.051), richer (TR = 1.059), and richest (TR = 1.038), all with 95% confidence intervals excluding 1, indicating statistical significance. In contrast, women living in households where the head had only primary education were significantly more likely to marry earlier (TR = 0.970, 95% CI: 0.949–0.991). Likewise, agricultural occupation of the household head was associated with earlier first marriage (TR = 0.968, 95% CI: 0.946–0.991), while other occupations were not significant.

In addition, contextual factors played an important role. Women residing in low-poverty communities tended to delay marriage (TR = 1.025, 95% CI: 1.005–1.046), whereas those in rural areas were significantly more likely to marry earlier than their urban counterparts (TR = 0.977, 95% CI: 0.960–0.995).

Furthermore, the random effects results underscore the presence of unobserved heterogeneity across clusters. The cluster-level variance was estimated at 0.5612 (95% CI: 0.1623–0.8326), and the intraclass correlation coefficient (ICC) was 0.1461 (95% CI: 0.0472–0.2523), suggesting that approximately 15% of the total variation in timing of first marriage can be attributed to differences between clusters. The shape parameter (γ) of the log-logistic distribution was 0.1079 (95% CI: 0.1045–0.1114), reflecting a non-monotonic hazard function, an increasing risk of marriage at early ages followed by a decline, typical in populations where early marriage is prevalent but levels off at older ages (Table 4).

Table 4: Multivariable analysis using the log-logistic survival model in Lesotho (2023-2024 LDHS dataset), 2025

Covariates	Categories	Coeff	St.err	P-value	TR (95%CI)
**Women's education level**	No education(ref)				1.00
	Primary	-0.0478	0.0353	0.176	0.953 (0.890–1.022)
	Secondary	-0.0019	0.0357	0.958	0.998 (0.931–1.070)
	Higher	0.1058	0.0377	**0.005**	1.112 (1.032–1.197)
**Women's working status**	No(ref)				1.00
	Yes	0.0329	0.0075	**<0.001**	1.033 (1.018–1.049)
**Head education level (n=3,226)**	No education(ref)				1.00
	Primary	-0.0308	0.0110	**0.005**	0.970 (0.949–0.991)
	Secondary	-0.0051	0.0127	0.689	0.995 (0.971–1.020)
	Higher	0.0264	0.0174	0.129	1.027 (0.992–1.062)
**Head occupation (n=3,226)**	Did not work(ref)				
	Professional	0.0166	0.0156	0.285	1.017 (0.986–1.048)
	Clerical	-0.0196	0.0272	0.472	0.981 (0.930–1.034)

	Sales and services	-0.0224	0.0143	0.119	0.978 (0.951–1.006)
	Skilled manual	0.0087	0.0111	0.431	1.009 (0.987–1.031)
	Unskilled manual	-0.0021	0.0091	0.819	0.998 (0.980–1.016)
	Agriculture	-0.0323	0.0118	**0.006**	0.968 (0.946–0.991)
**Mass media exposure**	No(ref)				1.00
	Yes	0.0184	0.0085	**0.030**	1.019 (1.002–1.036)
**Wealth index**	Poorest(ref)				
	Poorer	0.0229	0.0104	**0.028**	1.023 (1.002–1.044)
	Middle	0.0498	0.0118	<**0.001**	1.051 (1.027–1.076)
	Richer	0.0571	0.0138	<**0.001**	1.059 (1.030–1.088)
	Richest	0.0373	0.0153	**0.015**	1.038 (1.008–1.070)
**Community poverty level**	High(ref)				
	Low	0.0249	0.0102	**0.016**	1.025(1.005–1.046)
**Type of residence**	Urban(ref)				1.00
	Rural	-0.0231	0.0093	**0.013**	0.977 (0.960–0.995)
Shape parameter (γ)= 0.1079 (0.1045–0.1114)					
**Random effects**	**Parameters**		**Estimate**	**95% CI**	
	Cluster-level variance (σ^2)		0.5612	(0.1623–0.8326)	
	Intraclass correlation (ICC)		0.1461	(0.0472–0.2523)	

Coeff = Coefficient of factors; **St.err**: Standard error of coefficient; **TR** = Time Ratio
Boldface type (**p-value**) denotes study covariates and highlights statistically significant results for straightforward interpretation.

Discussions

Our results based on the log-logistic survival model reveals key determinants of marriage timing in Lesotho, reinforcing and expanding upon existing sociocultural narratives. Similar to the Cox frailty model insights, traditional norms and economic constraints shape marital transitions, particularly for women in rural areas, where parents often view marriage as a protective mechanism against sexual risks and a culturally sanctioned pathway to adulthood especially for girls perceived as less academically successful than boys [21-23]. However, our analysis further demonstrates that higher education, employment, media exposure, wealth status, and urban residence significantly delay first marriage, countering these traditional pressures. These factors empower women with greater autonomy, alternative opportunities, and exposure to modern norms

that prioritize personal and economic stability over early marital transitions. Conversely, agricultural household occupation and primary education of household heads accelerate marriage timing, reflecting entrenched rural livelihoods and limited aspirations beyond traditional gender roles.

Education played a critical role in influencing marriage timing. Women with higher educational attainment had significantly delayed first marriage compared to those with no formal education. This is consistent with other studies, such as those conducted in Ethiopia [22] and Nigeria [24], underscoring that education improves awareness of the consequences of early marriage and enhances decision-making capacity. Educated women are more likely to understand the risks of early marriage and resist familial or societal pressures. It is important to note that while primary and secondary education levels for women were not statistically significant at the 5% level, higher education retained a significant effect. This may suggest that only advanced education provides sufficient empowerment or life course changes to substantially influence marriage timing in this population.

Additionally, the educational level of the head of household was significantly associated with marriage timing. Contrary to some previous findings [21, 22, 25] , our results showed that women from households headed by individuals with only primary education were more likely to marry earlier, possibly reflecting limited support or understanding of the benefits of delayed marriage within such households. Employment status also emerged as a significant factor in delaying marriage. Working women were found to marry later than their non-working counterparts, supporting the notion that women's economic participation can enhance their independence and bargaining power within the household [22, 23, 26]. Similarly, media exposure was significantly

associated with later marriage, highlighting the role of information access in shaping social norms and attitudes toward early marriage.

Interestingly, our results highlighted a clear wealth gradient in marriage timing. Women from wealthier households experienced systematically delayed marriages, with each successive wealth quintile from poorer to richest associated with longer time to first marriage. This dose-response relationship suggests that improved economic conditions not only delay marriage but may also enhance access to education and reproductive health information, both of which contribute to women's empowerment. These findings contradict previous research [21, 22, 27] that found no significant association between wealth index and marriage timing. The observed relationship in our study may be attributed to improved living standards, better educational opportunities, and increased exposure to urban lifestyles among wealthier households, all of which can contribute to delayed marriage.

In contrast to previous literature [21-23], agricultural occupation of the household head was associated with earlier marriage among women, potentially due to traditional or economic motivations that encourage earlier unions in rural, agrarian settings. Notably, most of other occupation categories did not show significant associations at the 5% level of significance, suggesting that only specific household contexts exert measurable influence on marriage timing.

Community-level variables also demonstrated meaningful impacts. Women residing in low-poverty areas were more likely to delay marriage, possibly due to the availability of better services, educational infrastructure, and more progressive community norms. Furthermore, women living in rural areas were more likely to marry earlier than their urban counterparts, reflecting persistent

rural-urban disparities in access to education, employment, and awareness. The finding is also in line with other studies [21, 22].

The findings of this study have important clinical and policy implications for Lesotho, where early marriage continues to intersect with reproductive health risks and limited socioeconomic opportunities for women. Clinically, delaying marriage through investments in girls' higher education and employment opportunities can reduce early childbearing, associated maternal health complications, and unmet need for reproductive health services. From a policy perspective, the results underscore the urgency of scaling up multisectoral interventions that target rural and agrarian communities such as conditional cash transfers for girls' secondary and tertiary education, employment programs for young women, and public awareness campaigns via media. Furthermore, policies must address structural drivers, including poverty and entrenched gender norms, by integrating family and community-level education, especially targeting households where heads have only primary education or rely on agriculture. Strengthening urban-rural equity in education, information access, and economic opportunity is critical to shift traditional norms and empower women to make informed decisions about marriage timing. By addressing structural barriers education, poverty, rural isolation and reinforcing individual agency, Lesotho can align its policies with sustainable development goals to reduce early marriage and its adverse health and socioeconomic consequences.

Strengths and limitations of this study

This study has several strengths, including the use of a robust log-logistic survival model to analyze marriage timing, nationally representative data enhancing generalizability, and comprehensive examination of individual, household, and community-level factors that provide holistic insights

into determinants of marital transitions in Lesotho. The findings align with and expand existing literature while offering policy-relevant evidence on delaying marriage through education, employment, and poverty reduction. However, limitations include reliance on cross-sectional data susceptible to recall bias, inability to establish causality due to the observational design, potential unmeasured confounders like cultural norms, lack of qualitative data to explain underlying motivations, and broad variable categorizations that may obscure nuanced differences. Despite these constraints, the study provides valuable evidence to inform interventions, though future longitudinal and mixed-methods research could better elucidate causal pathways and contextual factors influencing marriage timing.

Conclusions

This study revealed that the median age at first marriage for women in Lesotho was 24 years, with a mean age of 23.67 years, indicating a relatively later marital transition compared to other sub-Saharan African countries. Key determinants influencing delayed marriage included higher education, employment, media exposure, wealth status and low-poverty areas, while agricultural livelihoods, primary education of household heads, and rural residence were associated with earlier unions. Given these findings, the government and relevant stakeholders should prioritize expanding access to secondary and higher education, creating economic opportunities for women, and targeted interventions in rural areas to further delay early marriage and mitigate its adverse consequences. Strengthening media outreach and community awareness programs could also help shift social norms, ensuring women have greater autonomy over their marital decisions. Addressing these factors will be crucial for advancing gender equality and improving health and socioeconomic outcomes for women in Lesotho.

Abbreviations

CI: Confidence Interval; ICC: Intra-class correlation; LDHS: Lesotho Demographic health survey; EAs: Enumeration areas; LLR: Log likelihood ratio; LR: Likelihood ratio; PSUs: Primary sampling units; SDGs: Sustainable development goals; TR: Time ratios; WHO: World Health Organizations

Declaration

Acknowledgements

We would like to thank the measure DHS program for providing the datasets.

Author Contributions

Conceptualization: Abdu Hailu Shibeshi, Zeytu Gashaw Asfaw, Temesgen Gebeyehu Wondmeneh, Mohammed Ahmed Ibrahim, Hana Emenew Gelaw, Zinabu Bekele Tadese, Abdulkerim Hassen Moloro, Abdulhakim Hora Hedato, Bizunesh Fantahun Kase

Data curation: Abdu Hailu Shibeshi, Zeytu Gashaw Asfaw, Temesgen Gebeyehu Wondmeneh, Mohammed Ahmed Ibrahim, Bizunesh Fantahun Kase

Formal analysis: Abdu Hailu Shibeshi, Bizunesh Fantahun Kase

Investigation: Abdu Hailu Shibeshi, Zeytu Gashaw Asfaw, Temesgen Gebeyehu Wondmeneh, Mohammed Ahmed Ibrahim, Bizunesh Fantahun Kase

Methodology: Abdu Hailu Shibeshi, Zeytu Gashaw Asfaw, Temesgen Gebeyehu Wondmeneh, Mohammed Ahmed Ibrahim, Bizunesh Fantahun Kase

Software: Abdu Hailu Shibeshi, Zeytu Gashaw Asfaw, Temesgen Gebeyehu Wondmeneh, Mohammed Ahmed Ibrahim, Bizunesh Fantahun Kase

Validation: Abdu Hailu Shibeshi, Zeytu Gashaw Asfaw, Temesgen Gebeyehu Wondmeneh, Mohammed Ahmed Ibrahim, Bizunesh Fantahun Kase

Visualization: Abdu Hailu Shibeshi, Zeytu Gashaw Asfaw, Temesgen Gebeyehu Wondmeneh, Mohammed Ahmed Ibrahim, Bizunesh Fantahun Kase

Writing – original draft: Abdu Hailu Shibeshi, Bizunesh Fantahun Kase

Writing – review & editing: Abdu Hailu Shibeshi, Zeytu Gashaw Asfaw, Temesgen Gebeyehu Wondmeneh, Mohammed Ahmed Ibrahim, Hana Emenew Gelaw, Zinabu Bekele Tadese, Abdulkerim Hassen Moloro, Abdulhakim Hora Hedato, Bizunesh Fantahun Kase

Availability of data and materials

Data is available online and you can access it from www.measuredhs.com.

Competing Interests

The authors of this study have declared that they have no conflict of interest

Funding

No funding was obtained for this study.

References

1. Plesons M, Travers E, Malhotra A, Finnie A, Maksud N, Chalasani S, et al. Updated research gaps on ending child marriage and supporting married girls for 2020–2030. 2021;18:1-7.
2. MacQuarrie KL, Mallick L, Allen C. Sexual and reproductive health in early and later adolescence: DHS data on youth age 10-19. 2017.
3. Fafard St-Germain A-A, Kirby RS, Urquia MLJPM. Reproductive health among married and unmarried mothers aged less than 18, 18–19, and 20–24 years in the United States, 2014–2019: A population-based cross-sectional study. 2022;19(3):e1003929.
4. Koski A, Clark S, Nandi AJP, review d. Has child marriage declined in sub-Saharan Africa? An analysis of trends in 31 countries. 2017:7-29.
5. Ministry of Health Maseru L, and The DHS Program,, ICF. Lesotho DHS, 2023-24 [dataset]. 2024.
6. ICF MoHLa. Lesotho Demographic and Health Survey 2023–24: Final Report. Maseru, Lesotho, and Rockville, Maryland, USA. Ministry of Health and ICF. 2024.

7. Likoti PM, Sibanyoni EKJIJoC, Sociology. Children as victims of forced marriages in Lesotho: A question of cultural practice or approval of child exploitation. 2020;9:723-34.
8. Budu E, Ahinkorah BO, Seidu A-A, Hagan Jr JE, Agbemavi W, Frimpong JB, et al. Child marriage and sexual autonomy among women in Sub-Saharan Africa: evidence from 31 demographic and health surveys. 2021;18(7):3754.
9. Gobena MG, Berelie YJRH. Modeling the determinant of time to age at first marriage among women in Ethiopia using Cox models with mixed effects. 2022;19(1):32.
10. Low AJ, Frederix K, McCracken S, Manyau S, Gummerson E, Radin E, et al. Association between severe drought and HIV prevention and care behaviors in Lesotho: A population-based survey 2016–2017. 2019;16(1):e1002727.
11. McPherson P. Economic cost of gender based violence against women and girls in Lesotho. 2023.
12. Motsima T, Malela-Majika J-CJAjorh. The effects of early first sexual intercourse amongst Lesotho women: evidence from the 2009 Lesotho demographic and health survey. 2016;20(2):34-42.
13. Islam MA, Rahman AJCE, Health G. Age at first marriage and fertility in developing countries: A meta analytical view of 15 Demographic and Health Surveys. 2020;8(3):775-9.
14. Tjamela KJ, Tingum EN, Rapapa MJIJoEPR, Review. The effects of early marriage and early fertility on women educational attainment: Evidence from Lesotho. 2021.
15. Worku MG, Tessema ZT, Teshale AB, Tesema GA, Yeshaw Y. Prevalence and associated factors of adolescent pregnancy (15–19 years) in East Africa: a multilevel analysis. BMC Pregnancy and Childbirth. 2021;21:1-8.
16. Kleinbaum DG, Klein M. Survival analysis a self-learning text: Springer; 1996.
17. Hosmer DW, Lemeshow S, May SJWSiP, Statistics. Applied survival analysis. 2008:60.
18. George B, Seals S, Aban IJJonc. Survival analysis and regression models. 2014;21(4):686-94.
19. Klein JP, Moeschberger ML. Survival analysis: techniques for censored and truncated data: Springer Science & Business Media; 2006.
20. Collett D. Modelling survival data in medical research: Chapman and Hall/CRC; 2023.
21. Gobena MG, Alemu YM. Analyzing factors associated with time to age at first marriage among women in Ethiopia: log logistic-gamma shared frailty model. BMC Women's Health. 2022;22(1):191.
22. Gobena MG, Berelie Y. Modeling the determinant of time to age at first marriage among women in Ethiopia using Cox models with mixed effects. Reproductive Health. 2022;19(1):32.
23. Mitiku Y, Kiffle D, Siyoum D, Birlie B. Determinants of time to first marriage among rural women in Ethiopia. Biomed Stat Inform. 2018;3(1):1.
24. Adedokun OA. Shifting patterns of age at first marriage and implications for family size in metropolitan Lagos (Nigeria). 1999.
25. Bates LM, Maselko J, Schuler SR. Women's education and the timing of marriage and childbearing in the next generation: evidence from rural Bangladesh. Studies in Family Planning. 2007;38(2):101-12.
26. Crowther MJ, Look MP, Riley RD. Multilevel mixed effects parametric survival models using adaptive Gauss–Hermite quadrature with application to recurrent events and individual participant data meta-analysis. Statistics in medicine. 2014;33(22):3844-58.
27. Haloi A, Limbu DK. Socio-economic factors influence the age at first marriage of Muslim women of a remote population from North-East India. Sociology. 2013;75:79.

Figure

Click here to access/download;Figure;Fig 1.tif

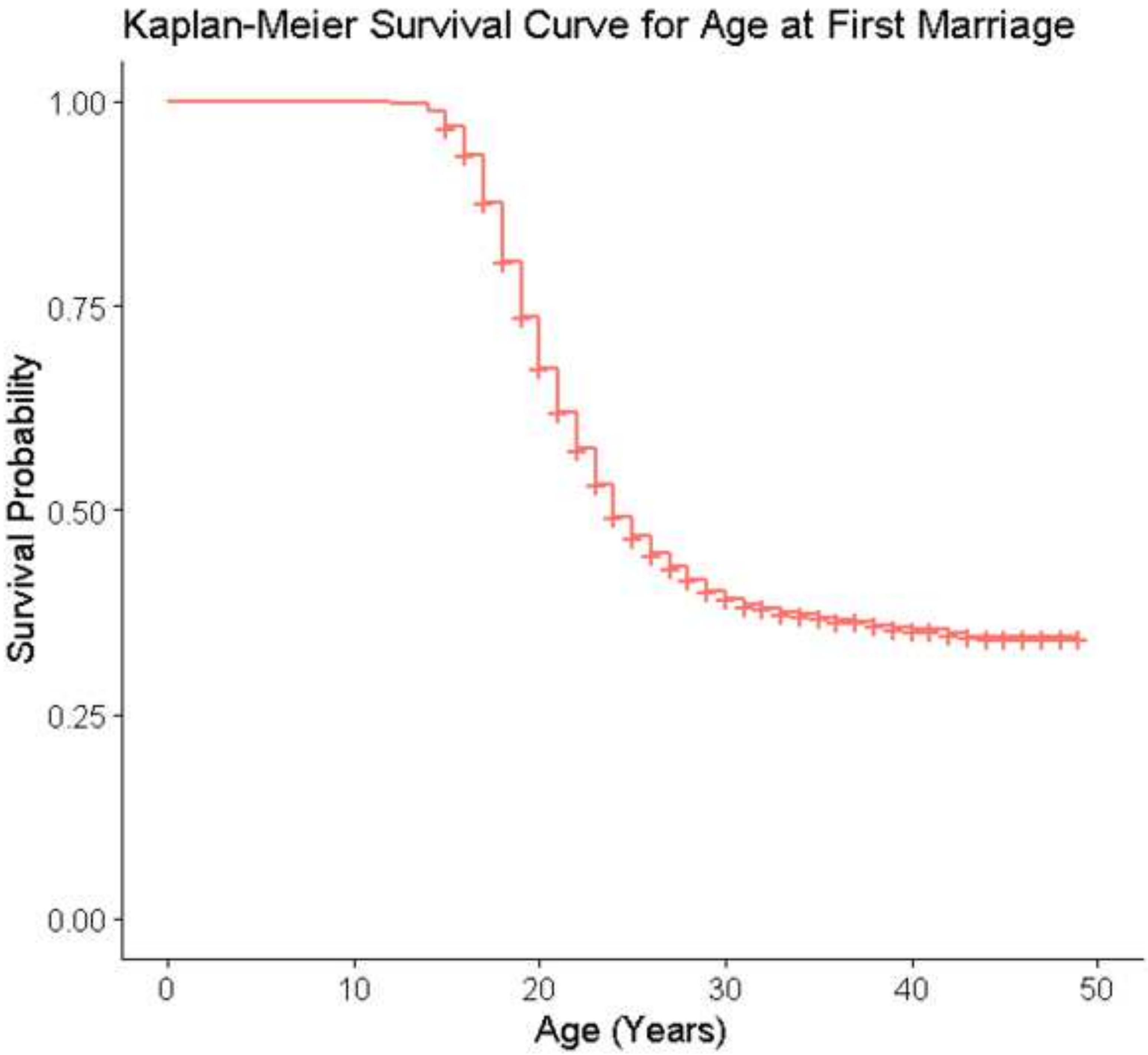

Figure

Click here to access/download;Figure;Fig 2.tif

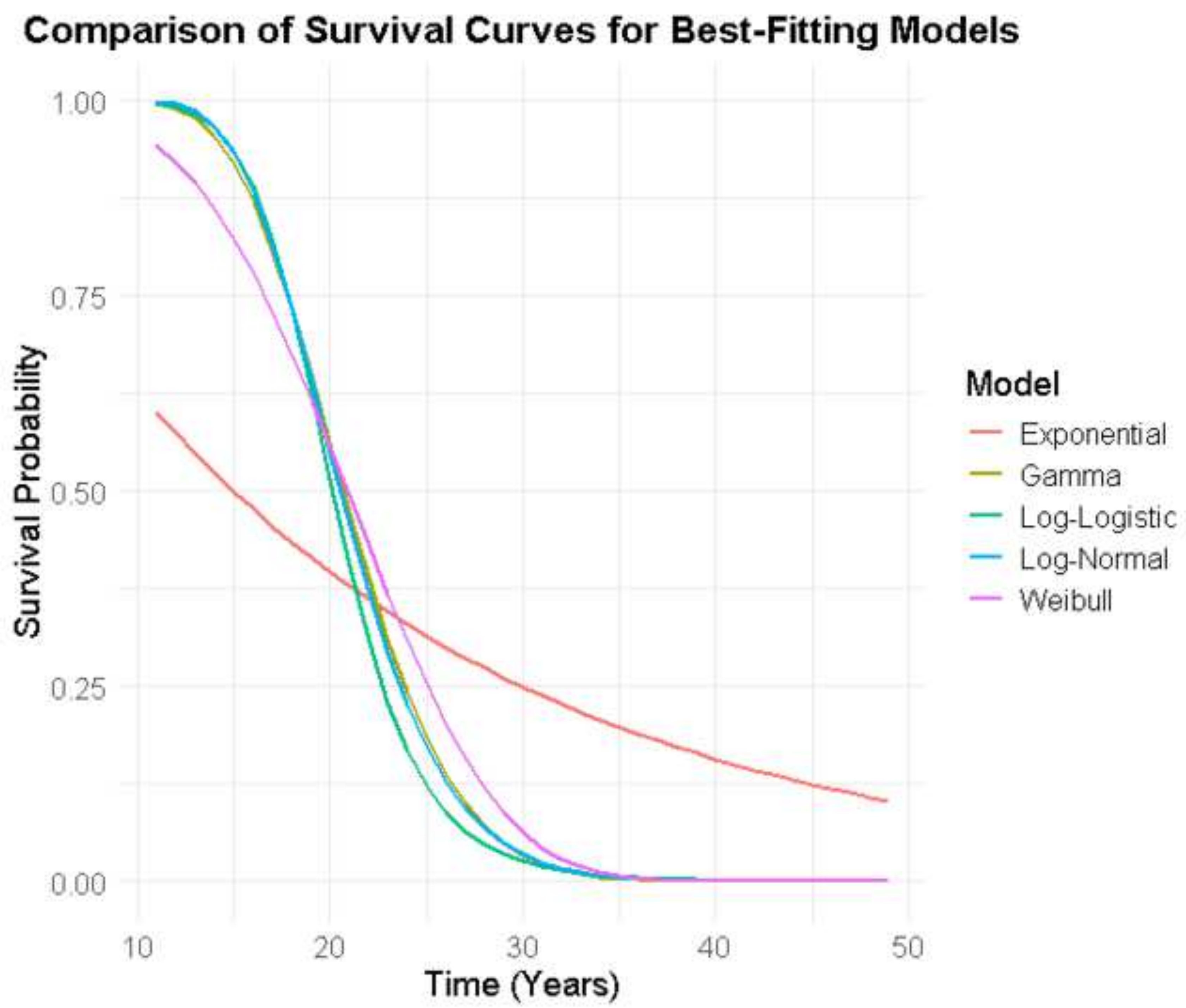

Click here to access/download

Supporting Information

STROBE_checklist_v4_combined.docx
