# Supplementary material for: Age at first marriage among women in Lesotho: Multilevel survival analysis- insight from 2023-24 demographic and health survey data
Source: PLoS One. 2026 Jul 22;21(7):e0354034. doi: 10.1371/journal.pone.0354034 (PMC13390838; doi:10.1371/journal.pone.0354034)
Supplement: Attachment — Submitted filename: Response to Reviewers.docx [file pone.0354034.s005.docx]

We would like to thank the reviewers and the editor for their thoughtful and constructive feedback on our manuscript, *“****Time to age at first marriage among women in Lesotho: Multilevel survival analysis (insights from recent 2023-24 Demographic and Health Survey data)****.”*

We have carefully considered all comments and suggestions. Below, we provide a detailed point-by-point response to each reviewer’s comment. Changes made to the manuscript are highlighted in **tracked changes manuscript**. We hope that these revisions adequately address the reviewers’ concerns and improve the clarity, rigor, and overall quality of the manuscript.

Review Comments to the Author

**Reviewer 1**
**Reviewer #1:** Time to age at first marriage among women in Lesotho study is important study for their community. Its important to improve the morbidity and mortality of mother and birth outcome. Clear methodology and discussion.

Response: We thank the reviewer for their positive comments and for recognizing the importance of our study. We appreciate the acknowledgment of our methodology and discussion, and we are encouraged that our work may contribute to improving maternal and child health outcomes in Lesotho.

**Reviewer 2**
**Reviewer #2:** Overall, the manuscript addresses an important demographic and public health issue using nationally representative DHS data and multilevel log-logistic accelerated failure time (AFT) modeling. The topic is timely, relevant to SDG 5.3, and expands the limited analytical work available for the study area. The methods are generally appropriate, the manuscript is well structured, and the results are clearly presented.
However, the following scientific, methodological, and reporting issues needs to be addressed to make the manuscript more suitable for publication.
1. Clarity on Study Design
The manuscript repeatedly refers to a “population-based cross-sectional design” while using retrospective time-to-event analysis.
Although this is permissible in DHS studies, the design description must be scientifically precise. I recommend the author should State explicitly that the study analyzed retrospective age-at-event data from a cross-sectional DHS survey, not longitudinal data.

Response: We thank the reviewer for this important comment. We have revised the manuscript to clarify the study design. Specifically, we now explicitly state that the study analyzed retrospective age-at-event (time-to-event) data collected from a cross-sectional DHS survey, rather than longitudinal data. This clarification has been added in the Abstract section (**lines 28-29)** and Methods section (**lines 98–100**) to ensure scientific precision and avoid any potential confusion regarding the study design.

2. Model Justification and Parametric Distributions
The manuscript includes long, textbook-style descriptions of exponential, Weibull, gamma, log-normal, and log-logistic distributions. These sections are unnecessary for a research paper and reduce readability.
The author should Remove mathematical derivations unless essential and condense model justification to 2–3 paragraphs focusing on:
1. Why the log-logistic distribution was optimal
2. Its empirical fit compared to alternatives
3. Its appropriateness for non-monotonic hazards in marriage timing

Response: We thank the reviewer for this constructive feedback. We have revised the Methods section (**lines 159-179**) by removing the extended mathematical derivations for exponential, Weibull, gamma, log-normal, and log-logistic distributions to improve readability.
3. Interpretation of Findings
Some interpretations overstate small effects. TR values close to 1.00 (e.g., 1.019 or 1.033) represent modest delays. The manuscript calls these “protective,” which is subjective.
I suggest the author should use neutral, quantitative language. For Example:
“Higher education was associated with a modest but statistically significant delay in marriage timing.”

Response: We thank the reviewer for this insightful comment. We have revised the Results sections (**lines 274-281)** to replace subjective terms such as “protective” with neutral, quantitative language. Specifically, Time Ratio (TR) values close to 1.00 are now interpreted as modest but statistically significant effects.
4. Inconsistency Between Observed and Survival-Adjusted Medians
The observed median age (21 years) differs from the survival-estimated median (24 years). This is expected due to right-censoring but not clearly explained.
The author should add a brief explanation in Results or Discussion to avoid confusion.

Response: We thank the reviewer for this insightful comment. We have revised the Results section (**lines 194-196**) to clarify the difference between the observed and survival-adjusted median ages. Specifically, we now explain that the survival-adjusted median (24 years) accounts for right-censoring, including women who had not yet married at the time of the survey, whereas the observed median (21 years) is based only on women who had already married. This distinction highlights that right-censored observations contribute to the survival analysis but not to the observed median, thereby explaining the slightly higher survival-based estimate.
5. Overemphasis on Household-Head Variables
The manuscript includes head occupation and head education but does not justify their conceptual relevance.
The authors should provide rationale based on household decision-making theories or remove non-essential predictors.

Response: We thank the reviewer for this important observation. We have revised the manuscript to clarify the conceptual relevance of household-head variables under independent variables (**lines 123-128**).
6. Community-Level Variables
Only two community-level variables were included (poverty level and residence). DHS allows aggregation of many others (education, media exposure, employment patterns, etc.).
The author should either justify why these two were selected or expand community-level variables.

Response: We thank the reviewer for this comment. We have clarified in the Methods section (**lines 128-133**)
7. Language and Redundancy
There are multiple redundant sections, overly long sentences, and repeated explanations. The authors should revise these for conciseness and scientific tone.

Response: We thank the reviewer for this valuable feedback. We have carefully revised the manuscript to remove redundant sections, shortened overly long sentences, and eliminated repeated explanations.
8. Minor Comments
a) Figures should include confidence bands and be referenced with clear captions.

Response: We appreciate this suggestion regarding the inclusion of confidence bands. In our analysis**, Fig. 1** presents the Kaplan–Meier survival curve, which is a nonparametric descriptive plot used to visualize the survival function without modeling assumptions. While confidence bands are sometimes included in such plots, our primary focus was on visual clarity and interpretability of the overall trend specifically, the steep decline in survival probability between ages 15–25, which indicates the period of highest marriage incidence. Adding confidence bands to this curve may obscure the main visual message, particularly given the large sample size where the confidence intervals would be very narrow.

To address the reviewer’s point, we have enhanced the figure caption to clearly describe the data, method, and interpretation, and we have included a note in the figure legend acknowledging that confidence intervals are available upon request. Additionally, we have added a sentence in the Results section noting the precision of the Kaplan–Meier estimate.

We have also ensured that **Fig. 2** which compares parametric survival models—is clearly captioned and referenced, with an emphasis on visual model comparison rather than uncertainty estimation, which is standard in model selection displays.

b) Tables require formatting improvement for readability, especially Table 3 and Table 4.

Response: We have reformatted Table 3 and Table 4 for improved clarity and readability.
c) Some references are incomplete or incorrectly formatted.

Response: We have reviewed and corrected all references in accordance with the journal’s style guidelines. Missing details (DOIs, volume/issue numbers, full journal names) have been added, and formatting inconsistencies have been resolved.
d) Avoid subjective terms such as “protective,” “persistent influence,” or “empower.”

Response: We have revised the manuscript to use more neutral and observational language. Terms like “**protective**” have been replaced with “**associated with later marriage**,” “**persistent influence**” with “**continued association**,” and “**empower**” with “**enhance autonomy**” or “**support decision-making**.” These changes appear throughout the **abstract**, **discussion**, and **conclusions**.
e) The Background section is lengthy and can be shortened.

Response: We have condensed the Background section by removing redundant statements and tightening the narrative, particularly in paragraphs describing DHS trends and regional context. The revised section is now more focused on the research gap and study rationale without sacrificing essential context.

**Reviewer 3**

**Reviewer #3:** This is a well written manuscript. The authors provided a concise background to the study, clearly defined the objective of the study, and described the methods in sufficient details for reproducibility. The results were clearly presented, and the discussions captured the essence of the findings and its relatedness to previous studies.
However, the authors introduced a new thought in the first paragraph of the conclusions indicating higher age at first marriage in Lesotho compared to other sub-Saharan African countries. This opinion had not been previously established in the discussions. I would encourage the authors to substantiate this claim in the discussion, before introducing it in their conclusions.

Response: We thank the reviewer for the positive evaluation of the manuscript and for this insightful comment. We agree that introducing a comparative interpretation in the Conclusions without prior discussion may reduce clarity. In response, we have revised the Discussion section (**in the last paragraph**) to explicitly substantiate the observation that the age at first marriage in Lesotho is relatively higher than in many other sub-Saharan African countries.

**Reviewer 4**

**Reviewer #4**: The manuscript can be passed as a good and scientific research that is publishable.
The title: I suggest that the authors should consider to re-write the title to be simple, catchy and bind every section of the paper of the paper tightly together (Age at first marriage among women in Lesotho: Multilevel survival analysis- insight from 2023-24 demographic and health survey data).
Data management and the statistical analyses were thoroughly done with the right data tools STATA and R studio needed to perform Multilevel survival analysis. I would suggest that all the calculations be taken to appendix section of the manuscript. Data analysis and result interpretation were excellently presented.
All the supporting data needed for this manuscript were available, appropriate and detailed enough for this research. Although the data used was a secondary data derived from Lesotho demographic and health survey data, I expect to see more elements of ethical considerations line 245: data accuracy, reduction of bias and respect for the different categories of women to feel protected and respected. The discussion drew insights from the result analysis for informed decision making and recommending strategic shift in policy making in Lesotho.

Response: We appreciate the suggestion to refine the title for greater clarity and cohesion. We have revised the title as recommended to: “**Age at first marriage among women in Lesotho: Multilevel survival analysis – insights from the 2023–24 demographic and health survey data**.”

**Regarding moving calculations to the Appendix**, Thank you for this suggestion. We have carefully considered the balance between readability and methodological transparency. While we agree that extensive formulas can sometimes overwhelm readers, the model justification and specification section including the rationale for selecting the log-logistic AFT model and its mathematical formulation is integral to understanding our analytical approach. Therefore, we have revised this content in the main Methods section narratively to ensure that readers can follow the logic behind our model choice without needing to refer to an appendix. We believe this maintains clarity and supports reproducibility.

We are grateful for the reviewer’s positive remarks regarding our data management, statistical rigor, use of STATA and R, result interpretation, and policy-relevant discussion.

Concerning on **Ethical Consideration**, Thank you for raising this important point. We have expanded the **Ethical Consideration** section in the revised manuscript to explicitly address data accuracy, bias reduction, and ethical protections for vulnerable participants. The updated section now includes details on the DHS program’s ethical protocols, informed consent, anonymization, and measures taken to ensure dignity and safety for all respondents, especially adolescent and rural women.

**Reviewer 5**

**Reviewer #5:** This manuscript addresses an important demographic and public health issue using recent DHS data and multilevel survival methods. While the topic and dataset are appropriate, there are major methodological, conceptual, and reporting issues that must be addressed before the manuscript can be considered for publication.
Response**: We sincerely thank the reviewer for these insightful and important methodological comments. We have carefully considered each point and have made revisions to the manuscript to address them directly, improving the clarity, justification, and transparency of our analysis.**
Lines 49–54; lines 252–259
The manuscript defines “time to age at first marriage” as duration from birth to first marriage (lines 49–54), yet later interprets results primarily as age at first marriage without revisiting the implications of this time origin.
Starting the survival clock at birth rather than at a socially relevant risk onset (e.g., adolescence) requires stronger justification, particularly given that marriage before puberty is biologically and socially implausible.

Response**:** We agree that this requires clarification. Using birth as the time origin (t=0) is a standard demographic convention in survival analyses of age at first marriage, as it allows for the modeling of the full life-course and facilitates the direct interpretation of the “survival time” as “age at event.” This approach is widely used in DHS-based studies and avoids introducing subjective or variable thresholds for risk onset (e.g., onset of adolescence, which varies individually and culturally). Our chosen model, the log-logistic Accelerated Failure Time model, is particularly suitable as it can accommodate a hazard function that remains near zero during childhood and rises sharply only in adolescence, aligning with biological and social reality.

To strengthen the justification, we have added the following clarification in the Methods section under "Survival Analysis": “**In this study, the survival time (time-to-event) was defined as the woman’s age at first marriage, measured in years since birth. While the risk of marriage is negligible in early childhood, using birth as the time origin is a standard demographic practice that provides a complete life-course perspective and allows the model to incorporate early-life determinants of later marital timing**.”

Furthermore, we have ensured consistency in the Results and Discussion by using the term "**age at first marriag**e" when presenting findings (e.g., median age, TRs), while retaining "**time to first marriage**" only in methodological contexts.

The reported minimum age at marriage of 11 years (line 257) raises concerns about data validity and plausibility checks, which are not described.

Response: We acknowledge this concern. The value of 11 years is an outlier in the lower tail of the distribution, representing 0.1% of married women in the sample. Such extremes can arise from recall error, social desirability bias, or data entry issues in large-scale surveys, but they are a known feature of self-reported retrospective data.

To address data validity, we have now explicitly described our data screening process in the Methods section under "**Data management and statistical analysis**": “**Prior to analysis, all variables were examined for logical inconsistencies and extreme values. The distribution of age at first marriage was reviewed, and extreme lower-bound values were identified for sensitivity testing.”**

The authors should clarify the rationale for using birth as the time origin, discuss potential left-truncation issues, and explicitly state whether any data validation or sensitivity analyses were conducted for extreme ages.
Response: Yes, we conducted a sensitivity analysis to ensure our results were not driven by extreme values. We performed a winsorizing procedure where all observations with an age at first marriage below 12 years were recoded to 12 years (a common threshold in child marriage research). The primary multilevel log-logistic AFT model was then re-estimated.

We have added a narrative summary of this analysis in the **Results section** following the presentation of descriptive statistics (**Table 2**): “**The observed minimum age of 11 years represents a rare outlier.  To ensure that this outlier did not unduly influence the model estimates, a sensitivity analysis was performed by recoding all ages below 12 years to 12 years. The re-estimated model yielded nearly identical results with respect to the median time to marriage, Time Ratios, significance of covariates, and variance components. This confirms that the findings are robust to the inclusion of extreme lower-bound values**.”

Left-truncation is not a concern in this study design. Because we define the time origin as birth and the event of interest (first marriage) could not logically occur before observation began, all individuals are “at risk” from time zero. There is no delayed entry into the risk set.

Lines 135–237
The Methods section includes extensive derivations of exponential, log-logistic, log-normal, and gamma distributions (lines 168–228).
Although the authors state that the log-logistic model was chosen as the “best-fitting” model (lines 309–315), no quantitative model comparison statistics (e.g., AIC, BIC, likelihood values) are reported.
The authors can condense this section (unless allowed by the Plos one), move mathematical derivations to supplementary material, and explicitly report formal model selection criteria.
Response: We agree that the detailed derivations of parametric distributions can be condensed to enhance readability while preserving methodological transparency. In accordance with PLOS ONE’s guidelines, which encourage clarity and conciseness without unnecessary mathematical detail, we have significantly shortened this section. We now provide only the essential formulas for the log-logistic model (our selected distribution) in the main text.

We appreciate this suggestion to strengthen the statistical justification for our model selection. In the original analysis, we relied on visual model comparison (presented in Fig. 2) and theoretical suitability (the non-monotonic hazard of the log-logistic model) to select the final model. This is a common and valid approach in survival analysis, especially when the visual fit clearly distinguishes one model.

However, we acknowledge that providing formal metrics like AIC and BIC would offer a more objective, quantitative basis for our choice. Since these values were not computed in the initial analysis, we would be happy to calculate and report the AIC and BIC for all compared models in a revised version of the manuscript, should the editor find this necessary.

Lines 254–261; lines 268–272
The survival-based median age at first marriage is reported as 24 years (line 254), while the observed median among married women is 21 years (line 259).
Although right-censoring is mentioned (lines 259–261), the implications of this discrepancy are not fully discussed.

Response: We have now explicitly clarified this discrepancy in the Results section (following Table 2). The added text explains: “**The observed median (21 years) is slightly lower than the survival-based estimate (24 years) due to right-censoring, as women who had not yet married at the time of the survey contribute to the survival analysis but not to the observed median**.”

In Table 3, median ages by subgroup often cluster around 18–21 years, which appears inconsistent with the narrative emphasis on later marriage.
The authors should provide clearer interpretation of survival-based vs observed medians and align conclusions with DHS-standard reporting practices to avoid overstatement.

Response: We acknowledge this apparent inconsistency and have revised the narrative to better contextualize the subgroup medians reported in Table 3.
Lines 113–121
Religion is listed as a potential independent variable (line 119) but is not included in the final models (Table 4).
Other well-established predictors of marriage timing (e.g., region/district, ethnicity, age at sexual debut) are not discussed.
Household head characteristics are modeled on a reduced subsample (n = 3,226), but implications of this restriction are not addressed.
The authors should justify variable selection more explicitly and acknowledge omitted confounders more clearly in the limitations.
Response: We acknowledge that the initial variable list was broader than what was included in the final model. Our variable selection was guided by **conceptual relevance**, **data availability,** and **prior literature**, with a focus on factors that are modifiable and relevant for policy intervention in the **Lesotho** context.

- **Religion:** While collected in the LDHS, preliminary univariate analyses showed that religion did not exhibit significant variation in marriage timing in this sample, and its inclusion did not improve model fit. Moreover, Lesotho is a relatively homogeneous country in terms of religious affiliation, which may explain the lack of predictive power.
- **Region/District:** We intentionally used community-level clustering (ICC) and place of residence (urban/rural) to capture geographical and contextual heterogeneity, which is methodologically more robust than including multiple district dummies in a multilevel survival model.
- **Ethnicity**: Ethnicity is not a significant social stratifies in Lesotho’s context in the same way it might be in other African settings. The LDHS does not emphasize ethnicity as a key demographic variable for Lesotho.
- **Age at Sexual Debut**: While an important life-course event, our study focuses on socioeconomic and structural determinants of marriage timing rather than behavioral or biological mediators. Including sexual debut would introduce issues of temporality and mediation, which are beyond the scope of this analysis.

Lines 319–435
Several statistically significant associations have very small effect sizes (e.g., TR = 1.019–1.059). These effects are repeatedly described as “protective” or “delaying” without discussion of practical significance.
Policy recommendations (lines 420–435) imply causal effects despite the cross-sectional design acknowledged earlier (lines 443–445).
Authors should discuss effect magnitude more critically, and frame policy implications as suggestive rather than prescriptive.
Response: We sincerely thank the reviewer for these critical and thoughtful observations. We have carefully revised the manuscript to address concerns regarding effect size interpretation, practical relevance, and appropriate framing of policy recommendations.
Lines: 48–54, 93–100, 254–261
The manuscript alternates between “time to age at first marriage,”
“age at first marriage,” and “time to first marriage.” The authors should be consistent.

Response: Thank you for highlighting this inconsistency in terminology. We acknowledge that the manuscript initially used multiple terms interchangeably, which may have caused confusion. We have now revised the manuscript to ensure consistent and precise use of terminology throughout.

This distinction clarifies that "**time to age at first marriage**" is our analytic variable (measured in person-years from birth), while "**age at first marriage**" is the demographic outcome we interpret and discuss. This aligns with standard practice in demographic survival analysis and improves readability without sacrificing methodological precision.

- **We believe these revisions significantly enhance the manuscript's scientific rigor, clarity, and adherence to publishing standards. Thank you again for your valuable feedback.**
