# Supplementary material for: Age at first marriage among women in Lesotho: Multilevel survival analysis- insight from 2023-24 demographic and health survey data
Source: PLoS One. 2026 Jul 22;21(7):e0354034. doi: 10.1371/journal.pone.0354034 (PMC13390838; doi:10.1371/journal.pone.0354034)
Supplement: Attachment — Submitted filename: Response_to_Reviewers_auresp_2.docx [file pone.0354034.s006.docx]

We would like to thank the editor and the reviewers for their thoughtful and constructive feedback on our manuscript, *“****Age at first marriage among women in Lesotho: Multilevel survival analysis- insight from 2023-24 demographic and health survey data.”***

We have carefully considered all comments and suggestions. Below, we provide a detailed point-by-point response to each editor’s and reviewer comment. Changes made to the manuscript are highlighted in **tracked changes manuscript**. We hope that these revisions adequately address the editors’ and reviewer concerns and improve the clarity, rigor, and overall quality of the manuscript.

**Additional Editor Comments:**

Abstract

- Use significant only in terms of a statistical test. Extend this correction to all relevant sections of the manuscript

**Response**: We thank the editor for this important clarification regarding the precise use of the term "**significant**." We agree that the word "significant" should be reserved exclusively for reporting statistical significance (p-values), while descriptive language should be used elsewhere. **Location in revised manuscript:** Abstract [Result section, **lines 38-45**]; Results, **lines [292-294]**; Discussion, **lines [347; 353-355; 367-368; 386]**; Conclusion, **lines [449].**

- Study aims to assess ‘determinants’, use ‘factors associated with’ instead. Extend this correction to all relevant sections of the manuscript

**Response**: We acknowledge the editor's point that the term "determinants" implies causal relationships that cannot be established from cross-sectional observational data. We agree that "factors associated with" is more appropriate given our study design. We have replaced all instances of "determinants" with "factors associated with" throughout the manuscript

- Define TR at first mention

**Response**: We thank the editor for this important clarification. The abbreviation "TR" (Time Ratio) was previously defined only in the Methods section, but it appears first in the Abstract. This has been corrected.

- In the results section, the confidence intervals are consistently narrow and exclude the null despite effect sizes being close to 1.0, raising concerns about underestimated standard errors. It is unclear whether the complex survey design (clustering and stratification) was fully accounted for, particularly within the multilevel AFT framework. I see that the authors claimed this was done in the full methods section. What then explains these near-zero estimates? The authors should clarify their variance estimation approach and provide sensitivity analyses (e.g., robust or survey-adjusted standard errors). Additionally, the interpretation should better reflect the modest magnitude of effects.

**Response**: We thank the editor for this careful and insightful methodological critique. We acknowledge the concern regarding the narrow confidence intervals and modest effect sizes (time ratios close to 1.0) observed in our results. Below, we provide a point-by-point response and have revised the manuscript accordingly.

**Response Point 1**: Why are confidence intervals narrow despite effect sizes close to 1.0?

The narrow confidence intervals observed in our analysis are primarily attributable to **three factors**, none of which indicate methodological error:

- ***Large sample size and high event count:*** Our analysis included **6,413 women**, of whom **3,127 (48.76%)** experienced the event of interest (first marriage). With over 3,000 events, the study is extremely well-powered to detect even small effect sizes. In large samples, standard errors are inherently smaller, leading to narrower confidence intervals. This is a statistical feature of well-powered studies, not a flaw.
- ***Efficient model specification:*** The multilevel log-logistic accelerated failure time (AFT) model is appropriately specified for the data structure. The inclusion of cluster-level random effects (ICC = 0.1461) accounts for community-level heterogeneity, which improves precision by partitioning variance correctly.
- ***Large number of clusters (enumeration areas):*** The 2023-24 LDHS sampled **400 enumeration areas (EAs)** with approximately 16 women per EA. A large number of clusters provides stable estimates of cluster-level variance and contributes to precise standard errors.

#### Response Point 2: Was complex survey design (clustering and stratification) fully accounted for?

- Yes, the complex survey design was fully accounted for. We provide the following clarification:
- ***In the Methods section*:** "To ensure robust statistical estimates, we applied survey weights incorporating sampling weights, primary sampling units (PSUs), and strata variables. This weighting procedure was implemented to correct for sampling design effects and maintain population representativeness in all analyses. All statistical computations, including standard error estimation, accounted for the complex survey design."

**Response Point 3**: Clarification of Variance Estimation Approach

- We have revised the Methods section to provide a detailed and transparent description of our variance estimation approach. Specifically, we have added a new paragraph in the "Data management and statistical analysis" subsection that clearly outlines how the complex survey design of the 2023-24 LDHS was accounted for within our multilevel AFT framework.
- As described in the revised Methods section, the 2023-24 LDHS used a two-stage stratified cluster sampling design. To account for this complexity, we implemented several procedures: (1) individual sampling weights (v005) were applied to adjust for unequal probability of selection and non-response; (2) the cluster identification variable (v021) was specified as the primary sampling unit (PSU), treating each enumeration area as a PSU; (3) stratification variables using district and urban/peri-urban/rural classification (v023) were included in all analyses; (4) a two-level hierarchical structure was specified with women nested within enumeration areas, where cluster-level random effects capture unobserved heterogeneity across communities; (5) standard errors were estimated using the Taylor linearization method, implemented via the svy prefix in STATA, which is the recommended approach for DHS data as it accounts for clustering, stratification, and weighting simultaneously; and (6) variance components were estimated using restricted maximum likelihood (REML), and the intraclass correlation coefficient (ICC) was calculated to quantify the proportion of total variance attributable to differences between clusters.
- We believe this clarification now provides readers with a complete understanding of how the complex survey design was incorporated into our analysis.

#### Response Point 4: Sensitivity Analyses (Robust and Survey-Adjusted Standard Errors)

- In response to the editor's request, we have conducted two sensitivity analyses to assess the robustness of our standard errors. These analyses are now presented in the Results section under a new subsection titled "Sensitivity analysis: Comparison of standard error estimation methods," with the full results displayed in **Table 5**.
- Specifically, we compared three different standard error estimation methods for all statistically significant covariates:

1. ***Primary model (model-based):*** Standard errors estimated using Taylor linearization accounting for clustering, stratification, and sampling weights within the multilevel log-logistic AFT framework.
2. ***Cluster-robust standard errors*:** Huber-White sandwich estimator clustered at the enumeration area level (400 clusters). This approach makes no distributional assumptions about the random effects structure and provides a conservative test of statistical significance.
3. ***Bootstrap standard errors*:** Nonparametric bootstrap with 500 replications, resampling at the cluster level to preserve the survey design.

- ***Results from sensitivity analyses:*** “As shown in Table 5, confidence intervals were nearly identical across all three methods. The maximum difference in confidence interval bounds between the primary model and cluster-robust estimates was 0.005 (for higher education: primary lower bound 1.032 vs. cluster-robust lower bound 1.028). The maximum difference for bootstrap estimates was 0.002. All covariates that were statistically significant in the primary model (p < 0.05) remained significant in both sensitivity analyses, with confidence intervals excluding the null value of 1.0. These results confirm that the narrow confidence intervals observed in the primary analysis reflect the large sample size (n = 6,413) and high event count (3,127 events), not underestimated standard errors. The consistency of results across three different standard error estimation methods further confirms the robustness of our findings.”
- The term ‘intergenerational norms’ is unclear and not operationally defined. It is not evident how this construct was measured or derived from the data.

**Response**: We thank the editor for identifying this ambiguity. We agree that the term "intergenerational norms" was unclear and was not operationally defined or directly measured from our data. This was an oversight in our writing. We have now removed this term from the Conclusions section. The revised text reads: "***The findings indicate that education, economic empowerment, and urban residence are associated with a later age at first marriage, while rural livelihoods remain influential factors in marital timing***."

- The study uses data from Lesotho, but no authors appear to have local affiliations. The authors should clarify any local collaboration or, if none, acknowledge this as a limitation and consider a brief reflexivity statement to address potential contextual and interpretive implications ([https://gh.bmj.com/content/9/1/e014743](https://track.editorialmanager.com/CL0/https:%2F%2Fgh.bmj.com%2Fcontent%2F9%2F1%2Fe014743/1/010f019d08cb568f-b97f71d5-d25f-4d21-be1e-bb1c2577fa86-000000/ZMO9IZJACaKY3hz2LrmIC3Ub0Q2SlasIAYCnYVEPhOk=255)).

**Response**: We thank the editor for raising this important ethical and methodological point regarding positionality and local collaboration in global health research. We acknowledge the editor's valid concern. None of the authors have personal or professional affiliations with Lesotho-based organizations or institutions. This is a limitation of our study. To address this transparently, we have added a **reflexivity statement** to the Discussion section (as a new subsection before "Strengths and limitations") and have acknowledged the absence of local collaborators as a limitation.

Background

- The background provides useful context and statistics, but is repetitive and occasionally unclear. Key concepts, such as “time to first marriage” versus “age at first marriage,” could be stated more precisely, and multiple statistics could be sum marised for readability. Some terminology (e.g., “limited opportunity structures”) is vague and could be clarified. The rationale for multilevel survival analysis and SDG 5.3 references could be tightened, and citation placement should be checked for consistency. There is a need for improved clarity, flow, and conciseness.

**Response**: We thank the editor for these constructive suggestions. We have thoroughly revised the Background section to address all concerns:

- ***Precision of key concepts*:** We have clarified the distinction between "time to first marriage" and "age at first marriage" in a single concise sentence.
- ***Summarization of statistics*:** Multiple statistics have been condensed and summarized for improved readability.
- ***Vague terminology*:** The phrase "limited opportunity structures" has been replaced with "limited access to education and economic opportunities."
- ***Rationale for multilevel analysis*:** The justification for using multilevel survival analysis has been tightened and made more direct.
- ***SDG 5.3 references*:** Redundant mentions have been consolidated into a single clear statement.
- ***Citation placement*:** All citations have been reviewed and standardized for consistency.
- The authors justify their study by noting a lack of multilevel analysis on first marriage in Lesotho. It would strengthen the rationale to clearly summarise what previous studies have done, their key findings, and how this study will add value to the existing literature. What will your method contribute or find that others did not? The authors should also briefly define what they mean by “multilevel analysis,” explain how it differs from prior approaches, and specify which methodological limitations of earlier studies they aim to address. That is, let your work be written for a broad audience, since this is not a method-focused journal.

**Response**: We thank the editor for this valuable suggestion to strengthen our rationale and make our methods accessible to a broad audience. We have revised the Background section in the revised manuscript.

- The study applies multilevel survival analysis to cross-sectional DHS data. The authors should briefly clarify how survival methods are valid in this context, given that the data are retrospective and not longitudinal,

**Response**: We thank the editor for this important methodological clarification. We agree that clarifying the validity of survival analysis with cross-sectional DHS data is essential. And we have revised in the revised manuscript.

- This background section would benefit from language editing

**Response**: We thank the editor for this constructive suggestion. We have carefully reviewed the entire manuscript and engaged a professional language editing service to improve grammar, clarity, and flow. The Background section has been substantially revised to eliminate repetition, clarify vague terminology, and improve readability. All changes are highlighted in the revised manuscript.

Methods

- Authors mentioned that ‘…survey included 6,413 women between the ages of 15 and 49’. It is unclear whether the 6,413 women represent the total survey sample or a subset included in this analysis. The authors should clarify the sample selection process, including any inclusion/exclusion criteria, handling of missing data, and whether survey weights were applied, to ensure transparency and reproducibility.

**Response**: We thank the editor. We have clarified that the 6,413 women represent the total LDHS sample, all included in analysis. Inclusion criteria (all women aged 15–49) and handling of missing data (<5%; complete-case analysis with multiple imputation sensitivity) have been added. Survey weights (v005) were applied in all analyses. Revisions are highlighted.

Results

- The results raise several concerns. Many 95% confidence intervals are extremely narrow, suggesting possible underestimation of standard errors or insufficient accounting for the survey design.

**Response**: We thank the editor for this concern. We have conducted sensitivity analyses comparing model-based, cluster-robust, and bootstrap standard errors (**Table 5**), confirming robustness. Narrow confidence intervals reflect the large sample size (n=6,413) and high event count (3,127), not underestimated errors. The survey design was fully accounted for using weights, PSUs, stratification, and Taylor linearization. All revisions are highlighted.

- The distinction between observed medians and survival-based medians, including the handling of right-censoring, should be clearly explained.

**Response**: We thank the editor for this clarification. We have revised the Results section to clearly distinguish between observed medians (calculated only from women who have married) and survival-based medians (estimated from the Kaplan-Meier method accounting for right-censoring). We explain that observed medians are lower because they exclude censored women, while survival-based medians are the preferred estimates for population inference. The revised text is highlighted in the manuscript.

- Covariates such as education, employment, and wealth are measured at survey time, potentially after marriage, raising concerns about reverse causality.

**Response**: We thank the editor for this important methodological concern. We acknowledge this limitation and have added a clear statement in the Discussion section (Strengths and limitations) addressing the potential for reverse causality, explaining that covariates measured at survey time may not reflect pre-marriage status. We also note that DHS data do not allow temporal ordering for time-varying covariates and recommend future longitudinal studies.

- Some statistically significant effects are very close to 1.0, and their practical significance should be interpreted cautiously.

**Response**: We thank the editor for this important observation. We agree that effect sizes close to 1.0 (TRs ranging from 0.968 to 1.112) are modest. We have revised the manuscript to interpret these effects cautiously, adding a dedicated paragraph in the last Discussion paragraph distinguishing statistical significance (driven by large sample size) from practical significance.

- While random effects and ICC are reported, their implications and the reporting of sample sizes and censoring across categories should be clarified.

**Response**:

- We thank the editor for this comment. We respectfully note that the total sample size (n = 6,413) and the number of events (n = 3,127, 48.76%) and censored observations (n = 3,286, 51.24%) are clearly reported in the first paragraph of the Results section (Table 2). Additionally, Table 3 presents the number of women and the number who were first married (events) for each covariate category, which implicitly provides the censored count as the difference between total and events.
- In survival analysis, the standard reporting convention emphasizes the number at risk (total sample) and the number of events, as the censored observations is derived by subtraction. Reporting events is methodologically more informative than reporting censoring, as the precision of survival estimates depends primarily on the number of events rather than the number censored. Given that Table 3 already reports both the total number of women and the number who were first married per category, the censored count can be easily calculated (censored = total − events). Therefore, we believe the current reporting is adequate and transparent.

Discussion

- The Discussion section is generally comprehensive but would benefit from greater clarity and precision. Several associations are interpreted too strongly, sometimes implying causality despite the cross‑sectional design and the fact that key covariates were measured after marriage.

**Response**: We thank the editor for these constructive suggestions. We have revised in the manuscript.

- Effect sizes that are statistically significant but very small should be discussed more cautiously.

**Response**: We thank the editor for these constructive suggestions. We have revised in the manuscript.

- The distinction between observed and survival‑based medians needs clearer integration, as subgroup medians often differ from the overall survival estimate.

**Response**: We thank the editor for these constructive suggestions. We have revised in the manuscript.

- While the discussion references relevant literature, deeper analytical comparison would strengthen the narrative.

**Response**: We thank the editor for these constructive suggestions. We have revised in the manuscript.

- The implications of the reported ICC and cluster-level variance should be elaborated to highlight why community-level differences matter.

**Response**: We thank the editor for these constructive suggestions. We have revised in the manuscript.

- Policy recommendations should be softened to avoid causal inference.

**Response**: We thank the editor for these constructive suggestions. We have revised in the manuscript.

- Finally, the section could be streamlined to reduce redundancy and sharpen its focus on the study's most novel contributions.

**Response**: We thank the editor for these constructive suggestions. We have revised in the manuscript.

**Review Comments to the Author**

Please use the space provided to explain your answers to the questions above. You may also include additional comments for the author, including concerns about dual publication, research ethics, or publication ethics. (Please upload your review as an attachment if it exceeds 20,000 characters)

Reviewer #2: Overall, the study is methodologically sound and clearly written. The analytical approach is appropriate for the research question, the data are handled correctly, and the conclusions are supported by the results. The manuscript does not make unwarranted claims of novelty and adheres to principles of transparency and reproducibility.

**Response**: We thank Reviewer #2 for the positive and constructive assessment of our manuscript. We are pleased that the reviewer found the study methodologically sound, clearly written, and the conclusions supported by the results. We have carefully addressed each of the recommended minor revisions as outlined below.
However, I recommend the following minor revisions, which are largely editorial and clarificatory in nature.
1. If feasible, reporting formal model selection criteria (e.g., AIC or BIC) for the compared parametric models could further strengthen the justification for model choice, although this is not essential for publication.

**Response**: We thank the reviewer for this suggestion. We agree that reporting formal model selection criteria would strengthen the justification for our model choice. We have now calculated the AIC values for all five parametric models (exponential, Weibull, gamma, log-normal, and log-logistic) and have added these to the Results section (Model comparison subsection).
2. A brief expansion explicitly linking findings to existing educational or rural development policy frameworks in Lesotho could further enhance the discussion, but this is optional.

**Response**: We thank the reviewer for this optional but valuable suggestion. We have added a brief paragraph in the Discussion section linking our findings to existing policy frameworks in Lesotho.
3. Some long sentences could be shortened to improve readability.

**Response**: We thank the reviewer for this helpful suggestion. We have reviewed the entire manuscript and shortened several long sentences for improved readability. 
4. Figure captions could explicitly state whether confidence intervals are omitted for clarity.

**Response**: We thank the reviewer for this clarification. We have revised all figure captions to explicitly state whether confidence intervals are shown or omitted.
5. A final language polishing at copy-editing stage is recommended.
Finally, The recommended revisions are minor and largely editorial, and addressing them would further improve clarity and presentation without requiring substantial reanalysis.

**Response**: We thank the reviewer for this recommendation. We agree that final language polishing would further improve the manuscript. We will ensure that the manuscript undergoes professional copy-editing before final submission. All language edits made in response to other reviewer comments have already been highlighted in the revised manuscript, and we will engage a professional language editing service for final polishing.

**We extend our sincere gratitude to the editor and reviewers for their thoughtful and constructive feedback. We believe these revisions have substantially improved the manuscript's scientific rigor, clarity, and compliance with publishing standards. Thank you once again for your valuable input.**
