# Supplementary material for: Age at first marriage among women in Lesotho: Multilevel survival analysis- insight from 2023-24 demographic and health survey data
Source: PLoS One. 2026 Jul 22;21(7):e0354034. doi: 10.1371/journal.pone.0354034 (PMC13390838; doi:10.1371/journal.pone.0354034)
Supplement: Attachment — Submitted filename: Response_to_Reviewers_auresp_3.docx [file pone.0354034.s007.docx]

We would like to thank the editor and the reviewers for their thoughtful and constructive feedback on our manuscript, *“****Age at first marriage among women in Lesotho: Multilevel survival analysis- insight from 2023-24 demographic and health survey data.”***

We have carefully considered all comments and suggestions. Below, we provide a detailed point-by-point response to each reviewer comment. Changes made to the manuscript are highlighted in **tracked changes manuscript**. We hope that these revisions adequately address the reviewer concerns and improve the clarity, rigor, and overall quality of the manuscript.

**Review Comments to the Author:**

Reviewer #2: Generally, this revised version represents a clear and substantial improvement over the previous submission. Your responses to the editor—particularly regarding standard error estimation and sensitivity analyses—are thorough and commendable.
That said, the following few refinements will further strengthen the manuscript.
1. Despite improvements, a few interpretations still lean toward causality. For example: Statements implying that education or employment delays marriage should consistently reflect association, not effect.
Hence, you should ensure consistent phrasing such as: “associated with later age at first marriage” and Avoid language suggesting direct influence or impact.

**Response**: We sincerely thank the reviewer for the thoughtful suggestions to further refine the language and interpretation. We have systematically revised the entire manuscript to replace any language that could imply causality (e.g., ‘delays,’ ‘accelerates,’ ‘leads to,’ ‘influence,’ ‘effect,’ ‘impact’) with strictly associative phrasing such as ‘associated with,’ ‘correlated with,’ or ‘related to.’ All instances in the Abstract, Results, Discussion, and Conclusions have been amended as detailed in the tracked-changes version. No causal interpretations remain.
2. You acknowledge that variables like education, wealth, and employment are measured at survey time. However, this limitation should be: More prominently emphasized and briefly reiterated when interpreting key findings
I Suggest you should add the following short reinforcing sentence in Discussion:
“These associations should be interpreted cautiously, as key covariates were measured after the occurrence of marriage for many participants.”

**Response**: We thank the reviewer for this important methodological observation. We have now prominently emphasized this limitation in the Strengths and limitations subsection and have added the reviewer's suggested sentence verbatim in the Discussion section, immediately following the interpretation of key findings.
3. You correctly note that TR values are close to 1.0. However, this caution should be made more prominent, not only in one paragraph
I Recommend you add the following sentence in the Results section acknowledging small effect sizes:
“Although statistically significant, the magnitude of these effects is modest.”

**Response**: We thank the reviewer for this important suggestion. We have now added the reviewer's suggested sentence verbatim in the **Results section**, immediately following the presentation of the key statistically significant findings in **Table 4**. Additionally, we have reinforced this caution in the **Abstract** and **Conclusions** sections to ensure readers encounter this important qualification at multiple points in the manuscript.
4. Some sections remain unnecessarily long, particularly:
• Background (repetition of DHS context and early marriage impacts)
• Methods (especially multilevel explanations)
• Discussion (repetition of findings)
I Recommend you should:
• Reduce redundancy by ~10–15% overall
• Focus on precision rather than repetition

**Response**: We thank the reviewer for this constructive feedback. We have systematically revised the manuscript to reduce redundancy and improve conciseness, focusing specifically on the three sections noted. **Background section, methods section, and discussion section.**
5. You report ICC = 0.1461, but interpretation could still be clearer if you explicitly state that: “Approximately 14.6% of the variation in age at first marriage is attributable to differences between communities.” This improves accessibility for non-technical readers.

**Response**: We thank the reviewer for this helpful suggestion to improve accessibility for non-technical readers. We have now revised the interpretation of the ICC throughout the manuscript to explicitly state the percentage of variation attributable to community-level differences.
6. The manuscript states: “N/A” initially, then later provides explanation, I recommend you Remove “N/A”, and keep only the full DHS-based ethical explanation for consistency

**Response**: Thank you for this valuable observation. We agree that the inclusion of “N/A” in the ethical considerations section was inconsistent with the detailed ethical explanation provided later in the manuscript. Accordingly, we have removed the “N/A” statement and retained a comprehensive ethical considerations section describing the DHS data access procedures, anonymization of participant information, informed consent process, and ethical safeguards implemented by the DHS Program.
7. For Language and Grammar, the manuscript has improved, but minor issues persist: Examples:
• “We conducted a retrospective time-to-event data…” (grammatical inconsistency)
• Occasional long sentences affecting readability
To address this, I think a Final professional copy-editing is still needed

**Response**: We thank the reviewer for identifying the remaining minor language issues. Specifically:

1. The **grammatical inconsistency** "We conducted a retrospective time-to-event data..." has been corrected to "We analyzed retrospective time-to-event data..."
2. **Long sentences affecting readability** have been shortened or broken down in five locations: the Background (paragraph 4), Methods (data management and model justification subsections), and Discussion (modest associations paragraph).
3. We agree that professional copy-editing is warranted. The manuscript will undergo a final professional copy-edit prior to submission to ensure the highest standards of language clarity and grammatical correctness.

All changes have been made in the tracked-changes version of the manuscript.

**We extend our sincere gratitude to the editor and reviewers for their thoughtful and constructive feedback. We believe these revisions have substantially improved the manuscript's scientific rigor, clarity, and compliance with publishing standards. Thank you once again for your valuable input.**
