# Supplementary material for: Age at first marriage among women in Lesotho: Multilevel survival analysis- insight from 2023-24 demographic and health survey data
Source: PLoS One. 2026 Jul 22;21(7):e0354034. doi: 10.1371/journal.pone.0354034 (PMC13390838; doi:10.1371/journal.pone.0354034)
Supplement: Attachment — Submitted filename: Response_to_Reviewers_auresp_4.docx [file pone.0354034.s008.docx]

**Manuscript Title:** Age at first marriage among women in Lesotho: Multilevel survival analysis- insight from 2023-24 demographic and health survey data

**Manuscript ID:** PONE-D-25-41860R3

**Journal:** PLOS ONE

**Corresponding Author:** Abdu Hailu Shibeshi

**Date:** June 26, 2026.

## Dear Dr. José Antonio Ortega,

Thank you for your thoughtful and constructive feedback on our manuscript (PONE-D-25-41860R3). We sincerely appreciate the time and effort you have invested in reviewing our work and providing detailed guidance for revision.

We have carefully addressed all the points raised in your editorial comments and have revised the manuscript accordingly. Below, we provide a point-by-point response to each of your specific concerns. All changes in the manuscript have been marked using track changes for your easy reference.

## Response to Academic Editor's Comments

### Comment 1: Internal Contradictions - Reference 17

**Editor's Comment:**

"Reference 17, on adolescent fertility, is the only reference provided for the choice of covariates. But this is very mistaken: adolescent fertility is generally studied with women currently exposed to the risk. Age at first marriage with women 15-49 analysis an event that in many cases happened 20 or 30 years ago. This makes interpretation radically different."

**Response**: We agree with the editor's observation. We have revised the literature review to include more appropriate references that specifically address age at first marriage rather than adolescent fertility. We have also expanded our justification for covariate selection; drawing from studies that examine marriage timing among women aged 15-49 in sub-Saharan African contexts

Comment 2: Variable Description Issues

**Editor's Comment:**

"There are also problems in the description of the variables: 'Head education Educational level', this is the minimum educational level a woman achieved..."

**Response**: We have removed the head education and head occupation variables entirely, as recommended by the editor. Therefore, this description issue is no longer relevant to the manuscript.

### Comment 3: Reverse Causality

**Editor's Comment:**

"For the reason mentioned first, it is not enough to pay lip service to reverse causality. One needs to avoid unnecessary use of variables that are of a short-nature or that have a confusing relationship to the variable under study."

**Response**: We have taken a more rigorous approach to addressing reverse causality. Specifically, we have removed three variables with clear temporal or measurement issues: mass media exposure, head occupation, and head education level. We have also added a birth cohort variable that is fixed and cannot be affected by marriage, which strengthens causal inference.

### Comment 4: Variables to Remove

**Editor's Comment:**

"In particular, in order to qualify for publication three variables that have clear problem should be removed from the model: mass media exposure, head occupation and head education level. The reason for the first is that current mass media exposure cannot be or remotely be a cause of a marriage that might have happened decades ago. The reason to remove the head variables is that they do not capture any meaningful association given that the head variables will be mostly those of the father (or similar figure) for currently unmarried women and the spouse in currently married (or the women in no longer married women). This creates a particular conflict in the kind of analysis used, survival analysis, since the relationship to the ego of the measured variable is dependent on the transition having occurred. I do not believe that any association so poorly measured can be of any value for policy."

**Response**: We fully agree with the editor's assessment and have removed all three variables from the manuscript. This decision is now clearly justified in the Methods section.

**Comment 5: Variable to Add - Birth Cohort**

**Editor's Comment:**

"One more variable should be added to capture changes over time. It is well known that marriage has been postponing in most countries of the world in the last decades including in Lesotho (see, for instance, doi:10.1007/s11113-013-9301-x), although a recent article points to continuity in the context of Lesotho (<https://doi.org/10.1007/s42650-025-00107-6>). Note that some of the variables currently included might be capturing changes over cohorts that are not included in the model as a possibility through other variables that change over cohorts and currently appear in the model. You should at least include some variable indicating the birth cohort, probably as a categorical variable for consistence with the current approach, to capture these possible changes over time."
**Response**: We have added a birth cohort variable to capture secular changes in marriage timing over time. Following the editor's suggestion, we created a categorical variable with 5-year intervals based on the woman's year of birth.

### Comment 6: Revised Manuscript Will Not Be Sent to Reviewers

**Editor's Comment:**

"To compensate for the delays in the revision process, the revised manuscript will not be sent to reviewers. I will personally evaluate whether these changes have been introduced."

**Response**: We appreciate the editor's willingness to personally evaluate the revised manuscript. We have carefully addressed all the points raised and hope that the revisions meet the editor's expectations.
