# Supplementary material for: Age at first marriage among women in Lesotho: Multilevel survival analysis- insight from 2023-24 demographic and health survey data
Source: PLoS One. 2026 Jul 22;21(7):e0354034. doi: 10.1371/journal.pone.0354034 (PMC13390838; doi:10.1371/journal.pone.0354034)
Supplement: Peer review history

# Peer review: Decision Letter 0

**DOI:** 10.1371/journal.pone.0354034.r001

**Authors:** Emmanuel Adewuyi

28 Dec 2025

PONE-D-25-41860

Time to age at first marriage among women in Lesotho: Multilevel survival analysis (insights from recent 2023-24 demographic and health survey data)

PLOS One

Dear Dr. Shibeshi,

Thank you for submitting your manuscript to PLOS ONE. After careful consideration, we feel that it has merit but does not fully meet PLOS ONE’s publication criteria as it currently stands. Therefore, we invite you to submit a revised version of the manuscript that addresses the points raised during the review process.

==============================

**ACADEMIC EDITOR:**

The reviewers have raised major conceptual and methodological concerns that need to be addressed. As noted by one reviewer, framing the outcome as ‘time to age at first marriage’ is conceptually unclear, given that age at first marriage is itself a time variable. The added value of survival analysis over simpler analytical approaches is not sufficiently justified, particularly in the context of cross-sectional DHS data, where key covariates (e.g. education, employment, and wealth) may be attained after marriage, raising concerns about reverse causality. In addition, the choice of the log-logistic model is largely supported by theoretical rather than empirical grounds, and claims of methodological novelty appear overstated and should be tempered. The manuscript would also benefit from improved clarity and focus. Terminology related to the outcome is used inconsistently, and interpretations in the Discussion at times extend beyond what can be supported by observational data. Finally, inconsistencies between the ethics statement in the manuscript text and the submission information need to be corrected.

==============================

Please submit your revised manuscript by Feb 11 2026 11:59PM. If you will need more time than this to complete your revisions, please reply to this message or contact the journal office at plosone@plos.org. When you're ready to submit your revision, log on to https://www.editorialmanager.com/pone/ and select the 'Submissions Needing Revision' folder to locate your manuscript file.

Please include the following items when submitting your revised manuscript:

- A letter that responds to each point raised by the academic editor and reviewer(s). You should upload this letter as a separate file labeled 'Response to Reviewers'.
- A marked-up copy of your manuscript that highlights changes made to the original version. You should upload this as a separate file labeled 'Revised Manuscript with Track Changes'.
- An unmarked version of your revised paper without tracked changes. You should upload this as a separate file labeled 'Manuscript'.

If you would like to make changes to your financial disclosure, please include your updated statement in your cover letter. Guidelines for resubmitting your figure files are available below the reviewer comments at the end of this letter.

If applicable, we recommend that you deposit your laboratory protocols in protocols.io to enhance the reproducibility of your results. Protocols.io assigns your protocol its own identifier (DOI) so that it can be cited independently in the future. For instructions see: https://journals.plos.org/plosone/s/submission-guidelines#loc-laboratory-protocols. Additionally, PLOS ONE offers an option for publishing peer-reviewed Lab Protocol articles, which describe protocols hosted on protocols.io. Read more information on sharing protocols at https://plos.org/protocols?utm_medium=editorial-email&utm_source=authorletters&utm_campaign=protocols.

We look forward to receiving your revised manuscript.

Kind regards,

Emmanuel Adewuyi

Academic Editor

PLOS One

Journal Requirements:

When submitting your revision, we need you to address these additional requirements.

1. Please ensure that your manuscript meets PLOS ONE's style requirements, including those for file naming. The PLOS ONE style templates can be found at

https://journals.plos.org/plosone/s/file?id=wjVg/PLOSOne_formatting_sample_main_body.pdf and

https://journals.plos.org/plosone/s/file?id=ba62/PLOSOne_formatting_sample_title_authors_affiliations.pdf

2. Please include captions for your Supporting Information files at the end of your manuscript, and update any in-text citations to match accordingly. Please see our Supporting Information guidelines for more information: http://journals.plos.org/plosone/s/supporting-information.

3. If the reviewer comments include a recommendation to cite specific previously published works, please review and evaluate these publications to determine whether they are relevant and should be cited. There is no requirement to cite these works unless the editor has indicated otherwise.

[Note: HTML markup is below. Please do not edit.]

Reviewers' comments:

Reviewer's Responses to Questions

**Comments to the Author**

1. Is the manuscript technically sound, and do the data support the conclusions?

The manuscript must describe a technically sound piece of scientific research with data that supports the conclusions. Experiments must have been conducted rigorously, with appropriate controls, replication, and sample sizes. The conclusions must be drawn appropriately based on the data presented.

Reviewer #1: Yes

Reviewer #2: Yes

Reviewer #3: Yes

Reviewer #4: Yes

Reviewer #5: Yes

**********

2. Has the statistical analysis been performed appropriately and rigorously?

Reviewer #1: Yes

Reviewer #2: Yes

Reviewer #3: Yes

Reviewer #4: Yes

Reviewer #5: Yes

**********

3. Have the authors made all data underlying the findings in their manuscript fully available?

The PLOS Data policy requires authors to make all data underlying the findings described in their manuscript fully available without restriction, with rare exception (please refer to the Data Availability Statement in the manuscript PDF file). The data should be provided as part of the manuscript or its supporting information, or deposited to a public repository. For example, in addition to summary statistics, the data points behind means, medians and variance measures should be available. If there are restrictions on publicly sharing data—e.g. participant privacy or use of data from a third party—those must be specified.

Reviewer #1: Yes

Reviewer #2: Yes

Reviewer #3: Yes

Reviewer #4: Yes

Reviewer #5: Yes

**********

4. Is the manuscript presented in an intelligible fashion and written in standard English?

PLOS ONE does not copyedit accepted manuscripts, so the language in submitted articles must be clear, correct, and unambiguous. Any typographical or grammatical errors should be corrected at revision, so please note any specific errors here.

Reviewer #1: Yes

Reviewer #2: Yes

Reviewer #3: Yes

Reviewer #4: Yes

Reviewer #5: Yes

**********

5. Review Comments to the Author

Please use the space provided to explain your answers to the questions above. You may also include additional comments for the author, including concerns about dual publication, research ethics, or publication ethics. (Please upload your review as an attachment if it exceeds 20,000 characters)

Reviewer #1: Time to age at first marriage among women in Lesotho study is important study for their community. Its important to improve the morbidity and mortality of mother and birth outcome. Clear methodology and discussion

Reviewer #2: Overall, the manuscript addresses an important demographic and public health issue using nationally representative DHS data and multilevel log-logistic accelerated failure time (AFT) modeling. The topic is timely, relevant to SDG 5.3, and expands the limited analytical work available for the study area. The methods are generally appropriate, the manuscript is well structured, and the results are clearly presented.

However, the following scientific, methodological, and reporting issues needs to be addressed to make the manuscript more suitable for publication.

1. Clarity on Study Design

The manuscript repeatedly refers to a “population-based cross-sectional design” while using retrospective time-to-event analysis.

Although this is permissible in DHS studies, the design description must be scientifically precise. I recommend the author should State explicitly that the study analyzed retrospective age-at-event data from a cross-sectional DHS survey, not longitudinal data.

2. Model Justification and Parametric Distributions

The manuscript includes long, textbook-style descriptions of exponential, Weibull, gamma, log-normal, and log-logistic distributions. These sections are unnecessary for a research paper and reduce readability.

The author should Remove mathematical derivations unless essential and condense model justification to 2–3 paragraphs focusing on:

1. Why the log-logistic distribution was optimal

2. Its empirical fit compared to alternatives

3. Its appropriateness for non-monotonic hazards in marriage timing

3. Interpretation of Findings

Some interpretations overstate small effects. TR values close to 1.00 (e.g., 1.019 or 1.033) represent modest delays. The manuscript calls these “protective,” which is subjective.

I suggest the author should use neutral, quantitative language. For Example:

“Higher education was associated with a modest but statistically significant delay in marriage timing.”

4. Inconsistency Between Observed and Survival-Adjusted Medians

The observed median age (21 years) differs from the survival-estimated median (24 years). This is expected due to right-censoring but not clearly explained.

The author should add a brief explanation in Results or Discussion to avoid confusion.

5. Overemphasis on Household-Head Variables

The manuscript includes head occupation and head education but does not justify their conceptual relevance.

The authors should provide rationale based on household decision-making theories or remove non-essential predictors.

6. Community-Level Variables

Only two community-level variables were included (poverty level and residence). DHS allows aggregation of many others (education, media exposure, employment patterns, etc.).

The author should either justify why these two were selected or expand community-level variables.

7. Language and Redundancy

There are multiple redundant sections, overly long sentences, and repeated explanations. The authors should revise these for conciseness and scientific tone.

8. Minor Comments

a) Figures should include confidence bands and be referenced with clear captions.

b) Tables require formatting improvement for readability, especially Table 3 and Table 4.

c) Some references are incomplete or incorrectly formatted.

d) Avoid subjective terms such as “protective,” “persistent influence,” or “empower.”

e) The Background section is lengthy and can be shortened.

Reviewer #3: This is a well written manuscript. The authors provided a concise background to the study, clearly defined the objective of the study, and described the methods in sufficient details for reproducibility. The results were clearly presented, and the discussions captured the essence of the findings and its relatedness to previous studies.

However, the authors introduced a new thought in the first paragraph of the conclusions indicating higher age at first marriage in Lesotho compared to other sub-Saharan African countries. This opinion had not been previously established in the discussions. I would encourage the authors to substantiate this claim in the discussion, before introducing it in their conclusions.

Reviewer #4: The manuscript can be passed as a good and scientific research that is publishable.

The title: I suggest that the authors should consider to re-write the title to be simple, catchy and bind every section of the paper of the paper tightly together (Age at first marriage among women in Lesotho: Multilevel survival analysis- insight from 2023-24 demographic and health survey data).

Data management and the statistical analyses were thoroughly done with the right data tools STATA and R studio needed to perform Multilevel survival analysis. I would suggest that all the calculations be taken to appendix section of the manuscript. Data analysis and result interpretation were excellently presented.

All the supporting data needed for this manuscript were available, appropriate and detailed enough for this research. Although the data used was a secondary data derived from Lesotho demographic and health survey data, I expect to see more elements of ethical considerations line 245: data accuracy, reduction of bias and respect for the different categories of women to feel protected and respected. The discussion drew insights from the result analysis for informed decision making and recommending strategic shift in policy making in Lesotho.

Reviewer #5: This manuscript addresses an important demographic and public health issue using recent DHS data and multilevel survival methods. While the topic and dataset are appropriate, there are major methodological, conceptual, and reporting issues that must be addressed before the manuscript can be considered for publication.

Lines 49–54; lines 252–259

The manuscript defines “time to age at first marriage” as duration from birth to first marriage (lines 49–54), yet later interprets results primarily as age at first marriage without revisiting the implications of this time origin.

Starting the survival clock at birth rather than at a socially relevant risk onset (e.g., adolescence) requires stronger justification, particularly given that marriage before puberty is biologically and socially implausible.

The reported minimum age at marriage of 11 years (line 257) raises concerns about data validity and plausibility checks, which are not described.

The authors should clarify the rationale for using birth as the time origin, discuss potential left-truncation issues, and explicitly state whether any data validation or sensitivity analyses were conducted for extreme ages.

Lines 135–237

The Methods section includes extensive derivations of exponential, log-logistic, log-normal, and gamma distributions (lines 168–228).

Although the authors state that the log-logistic model was chosen as the “best-fitting” model (lines 309–315), no quantitative model comparison statistics (e.g., AIC, BIC, likelihood values) are reported.

The authors can condense this section (unless allowed by the Plos one), move mathematical derivations to supplementary material, and explicitly report formal model selection criteria.

Lines 254–261; lines 268–272

The survival-based median age at first marriage is reported as 24 years (line 254), while the observed median among married women is 21 years (line 259).

Although right-censoring is mentioned (lines 259–261), the implications of this discrepancy are not fully discussed.

In Table 3, median ages by subgroup often cluster around 18–21 years, which appears inconsistent with the narrative emphasis on later marriage.

The authors should provide clearer interpretation of survival-based vs observed medians and align conclusions with DHS-standard reporting practices to avoid overstatement.

Lines 113–121

Religion is listed as a potential independent variable (line 119) but is not included in the final models (Table 4).

Other well-established predictors of marriage timing (e.g., region/district, ethnicity, age at sexual debut) are not discussed.

Household head characteristics are modeled on a reduced subsample (n = 3,226), but implications of this restriction are not addressed.

The authors should justify variable selection more explicitly and acknowledge omitted confounders more clearly in the limitations.

Lines 319–435

Several statistically significant associations have very small effect sizes (e.g., TR = 1.019–1.059).

These effects are repeatedly described as “protective” or “delaying” without discussion of practical significance.

Policy recommendations (lines 420–435) imply causal effects despite the cross-sectional design acknowledged earlier (lines 443–445).

Authors should discuss effect magnitude more critically, and frame policy implications as suggestive rather than prescriptive.

Lines: 48–54, 93–100, 254–261

The manuscript alternates between “time to age at first marriage,”

“age at first marriage,” and “time to first marriage.” The authors should be consistent.

**********

6. PLOS authors have the option to publish the peer review history of their article (what does this mean?). If published, this will include your full peer review and any attached files.

If you choose “no”, your identity will remain anonymous but your review may still be made public.

**Do you want your identity to be public for this peer review?**  For information about this choice, including consent withdrawal, please see our Privacy Policy.

Reviewer #1: No

Reviewer #2: **Yes:** Lawan Adamu

Reviewer #3: **Yes:** Oluwasanmi Akanji Adedokun

Reviewer #4: **Yes:** Adegoke Olufemi Dickson PhD

Reviewer #5: No

**********

[NOTE: If reviewer comments were submitted as an attachment file, they will be attached to this email and accessible via the submission site. Please log into your account, locate the manuscript record, and check for the action link "View Attachments". If this link does not appear, there are no attachment files.]

To ensure your figures meet our technical requirements, please review our figure guidelines: https://journals.plos.org/plosone/s/figures

You may also use PLOS’s free figure tool, NAAS, to help you prepare publication quality figures: https://journals.plos.org/plosone/s/figures#loc-tools-for-figure-preparation.

NAAS will assess whether your figures meet our technical requirements by comparing each figure against our figure specifications.

Attachment Submitted filename: PONE-D-25-41860_Lesotho_2.pdf

# Author comment: Author response to Decision Letter 1

**DOI:** 10.1371/journal.pone.0354034.r002

3 Feb 2026

We would like to thank the reviewers and the editor for their thoughtful and constructive feedback on our manuscript, “Time to age at first marriage among women in Lesotho: Multilevel survival analysis (insights from recent 2023-24 Demographic and Health Survey data).”

We have carefully considered all comments and suggestions. Below, we provide a detailed point-by-point response to each reviewer’s comment. Changes made to the manuscript are highlighted in tracked changes manuscript. We hope that these revisions adequately address the reviewers’ concerns and improve the clarity, rigor, and overall quality of the manuscript.

Review Comments to the Author

Reviewer 1

Reviewer #1: Time to age at first marriage among women in Lesotho study is important study for their community. Its important to improve the morbidity and mortality of mother and birth outcome. Clear methodology and discussion.

Response: We thank the reviewer for their positive comments and for recognizing the importance of our study. We appreciate the acknowledgment of our methodology and discussion, and we are encouraged that our work may contribute to improving maternal and child health outcomes in Lesotho.

Reviewer 2

Reviewer #2: Overall, the manuscript addresses an important demographic and public health issue using nationally representative DHS data and multilevel log-logistic accelerated failure time (AFT) modeling. The topic is timely, relevant to SDG 5.3, and expands the limited analytical work available for the study area. The methods are generally appropriate, the manuscript is well structured, and the results are clearly presented.

However, the following scientific, methodological, and reporting issues needs to be addressed to make the manuscript more suitable for publication.

1. Clarity on Study Design

The manuscript repeatedly refers to a “population-based cross-sectional design” while using retrospective time-to-event analysis.

Although this is permissible in DHS studies, the design description must be scientifically precise. I recommend the author should State explicitly that the study analyzed retrospective age-at-event data from a cross-sectional DHS survey, not longitudinal data.

Response: We thank the reviewer for this important comment. We have revised the manuscript to clarify the study design. Specifically, we now explicitly state that the study analyzed retrospective age-at-event (time-to-event) data collected from a cross-sectional DHS survey, rather than longitudinal data. This clarification has been added in the Abstract section (lines 28-29) and Methods section (lines 98–100) to ensure scientific precision and avoid any potential confusion regarding the study design.

2. Model Justification and Parametric Distributions

The manuscript includes long, textbook-style descriptions of exponential, Weibull, gamma, log-normal, and log-logistic distributions. These sections are unnecessary for a research paper and reduce readability.

The author should Remove mathematical derivations unless essential and condense model justification to 2–3 paragraphs focusing on:

1. Why the log-logistic distribution was optimal

2. Its empirical fit compared to alternatives

3. Its appropriateness for non-monotonic hazards in marriage timing

Response: We thank the reviewer for this constructive feedback. We have revised the Methods section (lines 159-179) by removing the extended mathematical derivations for exponential, Weibull, gamma, log-normal, and log-logistic distributions to improve readability.

3. Interpretation of Findings

Some interpretations overstate small effects. TR values close to 1.00 (e.g., 1.019 or 1.033) represent modest delays. The manuscript calls these “protective,” which is subjective.

I suggest the author should use neutral, quantitative language. For Example:

“Higher education was associated with a modest but statistically significant delay in marriage timing.”

Response: We thank the reviewer for this insightful comment. We have revised the Results sections (lines 274-281) to replace subjective terms such as “protective” with neutral, quantitative language. Specifically, Time Ratio (TR) values close to 1.00 are now interpreted as modest but statistically significant effects.

4. Inconsistency Between Observed and Survival-Adjusted Medians

The observed median age (21 years) differs from the survival-estimated median (24 years). This is expected due to right-censoring but not clearly explained.

The author should add a brief explanation in Results or Discussion to avoid confusion.

Response: We thank the reviewer for this insightful comment. We have revised the Results section (lines 194-196) to clarify the difference between the observed and survival-adjusted median ages. Specifically, we now explain that the survival-adjusted median (24 years) accounts for right-censoring, including women who had not yet married at the time of the survey, whereas the observed median (21 years) is based only on women who had already married. This distinction highlights that right-censored observations contribute to the survival analysis but not to the observed median, thereby explaining the slightly higher survival-based estimate.

5. Overemphasis on Household-Head Variables

The manuscript includes head occupation and head education but does not justify their conceptual relevance.

The authors should provide rationale based on household decision-making theories or remove non-essential predictors.

Response: We thank the reviewer for this important observation. We have revised the manuscript to clarify the conceptual relevance of household-head variables under independent variables (lines 123-128).

6. Community-Level Variables

Only two community-level variables were included (poverty level and residence). DHS allows aggregation of many others (education, media exposure, employment patterns, etc.).

The author should either justify why these two were selected or expand community-level variables.

Response: We thank the reviewer for this comment. We have clarified in the Methods section (lines 128-133)

7. Language and Redundancy

There are multiple redundant sections, overly long sentences, and repeated explanations. The authors should revise these for conciseness and scientific tone.

Response: We thank the reviewer for this valuable feedback. We have carefully revised the manuscript to remove redundant sections, shortened overly long sentences, and eliminated repeated explanations.

8. Minor Comments

a) Figures should include confidence bands and be referenced with clear captions.

Response: We appreciate this suggestion regarding the inclusion of confidence bands. In our analysis, Fig. 1 presents the Kaplan–Meier survival curve, which is a nonparametric descriptive plot used to visualize the survival function without modeling assumptions. While confidence bands are sometimes included in such plots, our primary focus was on visual clarity and interpretability of the overall trend specifically, the steep decline in survival probability between ages 15–25, which indicates the period of highest marriage incidence. Adding confidence bands to this curve may obscure the main visual message, particularly given the large sample size where the confidence intervals would be very narrow.

To address the reviewer’s point, we have enhanced the figure caption to clearly describe the data, method, and interpretation, and we have included a note in the figure legend acknowledging that confidence intervals are available upon request. Additionally, we have added a sentence in the Results section noting the precision of the Kaplan–Meier estimate.

We have also ensured that Fig. 2 which compares parametric survival models—is clearly captioned and referenced, with an emphasis on visual model comparison rather than uncertainty estimation, which is standard in model selection displays.

b) Tables require formatting improvement for readability, especially Table 3 and Table 4.

Response: We have reformatted Table 3 and Table 4 for improved clarity and readability.

c) Some references are incomplete or incorrectly formatted.

Response: We have reviewed and corrected all references in accordance with the journal’s style guidelines. Missing details (DOIs, volume/issue numbers, full journal names) have been added, and formatting inconsistencies have been resolved.

d) Avoid subjective terms such as “protective,” “persistent influence,” or “empower.”

Response: We have revised the manuscript to use more neutral and observational language. Terms like “protective” have been replaced with “associated with later marriage,” “persistent influence” with “continued association,” and “empower” with “enhance autonomy” or “support decision-making.” These changes appear throughout the abstract, discussion, and conclusions.

e) The Background section is lengthy and can be shortened.

Response: We have condensed the Background section by removing redundant statements and tightening the narrative, particularly in paragraphs describing DHS trends and regional context. The revised section is now more focused on the research gap and study rationale without sacrificing essential context.

Reviewer 3

Reviewer #3: This is a well written manuscript. The authors provided a concise background to the study, clearly defined the objective of the study, and described the methods in sufficient details for reproducibility. The results were clearly presented, and the discussions captured the essence of the findings and its relatedness to previous studies.

However, the authors introduced a new thought in the first paragraph of the conclusions indicating higher age at first marriage in Lesotho compared to other sub-Saharan African countries. This opinion had not been previously established in the discussions. I would encourage the authors to substantiate this claim in the discussion, before introducing it in their conclusions.

Response: We thank the reviewer for the positive evaluation of the manuscript and for this insightful comment. We agree that introducing a comparative interpretation in the Conclusions without prior discussion may reduce clarity. In response, we have revised the Discussion section (in the last paragraph) to explicitly substantiate the observation that the age at first marriage in Lesotho is relatively higher than in many other sub-Saharan African countries.

Reviewer 4

Reviewer #4: The manuscript can be passed as a good and scientific research that is publishable.

The title: I suggest that the authors should consider to re-write the title to be simple, catchy and bind every section of the paper of the paper tightly together (Age at first marriage among women in Lesotho: Multilevel survival analysis- insight from 2023-24 demographic and health survey data).

Data management and the statistical analyses were thoroughly done with the right data tools STATA and R studio needed to perform Multilevel survival analysis. I would suggest that all the calculations be taken to appendix section of the manuscript. Data analysis and result interpretation were excellently presented.

All the supporting data needed for this manuscript were available, appropriate and detailed enough for this research. Although the data used was a secondary data derived from Lesotho demographic and health survey data, I expect to see more elements of ethical considerations line 245: data accuracy, reduction of bias and respect for the different categories of women to feel protected and respected. The discussion drew insights from the result analysis for informed decision making and recommending strategic shift in policy making in Lesotho.

Response: We appreciate the suggestion to refine the title for greater clarity and cohesion. We have revised the title as recommended to: “Age at first marriage among women in Lesotho: Multilevel survival analysis – insights from the 2023–24 demographic and health survey data.”

Regarding moving calculations to the Appendix, Thank you for this suggestion. We have carefully considered the balance between readability and methodological transparency. While we agree that extensive formulas can sometimes overwhelm readers, the model justification and specification section including the rationale for selecting the log-logistic AFT model and its mathematical formulation is integral to understanding our analytical approach. Therefore, we have revised this content in the main Methods section narratively to ensure that readers can follow the logic behind our model choice without needing to refer to an appendix. We believe this maintains clarity and supports reproducibility.

We are grateful for the reviewer’s positive remarks regarding our data management, statistical rigor, use of STATA and R, result interpretation, and policy-relevant discussion.

Concerning on Ethical Consideration, Thank you for raising this important point. We have expanded the Ethical Consideration section in the revised manuscript to explicitly address data accuracy, bias reduction, and ethical protections for vulnerable participants. The updated section now includes details on the DHS program’s ethical protocols, informed consent, anonymization, and measures taken to ensure dignity and safety for all respondents, especially adolescent and rural women.

Reviewer 5

Reviewer #5: This manuscript addresses an important demographic and public health issue using recent DHS data and multilevel survival methods. While the topic and dataset are appropriate, there are major methodological, conceptual, and reporting issues that must be addressed before the manuscript can be considered for publication.

Response: We sincerely thank the reviewer for these insightful and important methodological comments. We have carefully considered each point and have made revisions to the manuscript to address them directly, improving the clarity, justification, and transparency of our analysis.

Lines 49–54; lines 252–259

The manuscript defines “time to age at first marriage” as duration from birth to first marriage (lines 49–54), yet later interprets results primarily as age at first marriage without revisiting the implications of this time origin.

Starting the survival clock at birth rather than at a socially relevant risk onset (e.g., adolescence) requires stronger justification, particularly given that marriage before puberty is biologically and socially implausible.

Response: We agree that this requires clarification. Using birth as the time origin (t=0) is a standard demographic convention in survival analyses of age at first marriage, as it allows for the modeling of the full life-course and facilitates the direct interpretation of the “survival time” as “age at event.” This approach is widely used in DHS-based studies and avoids introducing subjective or variable thresholds for risk onset (e.g., onset of adolescence, which varies individually and culturally). Our chosen model, the log-logistic Accelerated Failure Time model, is particularly suitable as it can accommodate a hazard function that remains near zero during childhood and rises sharply only in adolescence, aligning with biological and social reality.

To strengthen the justification, we have added the following clarification in the Methods section under "Survival Analysis": “In this study, the survival time (time-to-event) was defined as the woman’s age at first marriage, measured in years since birth. While the risk of marriage is negligible in early childhood, using birth as the time origin is a standard demographic practice that provides a complete life-course perspective and allows the model to incorporate early-life determinants of later marital timing.”

Furthermore, we have ensured consistency in the Results and Discussion by using the term "age at first marriage" when presenting findings (e.g., median age, TRs), while retaining "time to first marriage" only in methodological contexts.

The reported minimum age at marriage of 11 years (line 257) raises concerns about data validity and plausibility checks, which are not described.

Response: We acknowledge this concern. The value of 11 years is an outlier in the lower tail of the distribution, representing 0.1% of married women in the sample. Such extremes can arise from recall error, social desirability bias, or data entr

Attachment Submitted filename: Response to Reviewers.docx

# Peer review: Decision Letter 1

**DOI:** 10.1371/journal.pone.0354034.r003

**Authors:** Emmanuel Adewuyi

19 Mar 2026

PONE-D-25-41860R1

Age at first marriage among women in Lesotho: Multilevel survival analysis- insight from 2023-24 demographic and health survey data

PLOS One

Dear Dr. Shibeshi,

Thank you for submitting your manuscript to PLOS ONE. After careful consideration, we feel that it has merit but does not fully meet PLOS ONE’s publication criteria as it currently stands. Therefore, we invite you to submit a revised version of the manuscript that addresses the points raised during the review process.

==============================

**ACADEMIC EDITOR:**

- The comments made previously by the editor were not  addressed. Authors need to clearly address all those comments and provide a response in their revision. Here are the comments. "The reviewers have raised major conceptual and methodological concerns that need to be addressed. As noted by one reviewer, framing the outcome as ‘time to age at first marriage’ is conceptually unclear, given that age at first marriage is itself a time variable. The added value of survival analysis over simpler analytical approaches is not sufficiently justified, particularly in the context of cross-sectional DHS data, where key covariates (e.g. education, employment, and wealth) may be attained after marriage, raising concerns about reverse causality. In addition, the choice of the log-logistic model is largely supported by theoretical rather than empirical grounds, and claims of methodological novelty appear overstated and should be tempered. The manuscript would also benefit from improved clarity and focus. Terminology related to the outcome is used inconsistently, and interpretations in the Discussion at times extend beyond what can be supported by observational data. Finally, inconsistencies between the ethics statement in the manuscript text and the submission information need to be corrected."
- There are additional editor comments below that authors need to respond to alongside the comments of one of the reviewers.

-->==============================

Please submit your revised manuscript by May 03 2026 11:59PM. If you will need more time than this to complete your revisions, please reply to this message or contact the journal office at plosone@plos.org. When you're ready to submit your revision, log on to https://www.editorialmanager.com/pone/ and select the 'Submissions Needing Revision' folder to locate your manuscript file.

Please include the following items when submitting your revised manuscript:

- A letter that responds to each point raised by the academic editor and reviewer(s). You should upload this letter as a separate file labeled 'Response to Reviewers'.
- A marked-up copy of your manuscript that highlights changes made to the original version. You should upload this as a separate file labeled 'Revised Manuscript with Track Changes'.
- An unmarked version of your revised paper without tracked changes. You should upload this as a separate file labeled 'Manuscript'.

If you would like to make changes to your financial disclosure, please include your updated statement in your cover letter. Guidelines for resubmitting your figure files are available below the reviewer comments at the end of this letter.

If applicable, we recommend that you deposit your laboratory protocols in protocols.io to enhance the reproducibility of your results. Protocols.io assigns your protocol its own identifier (DOI) so that it can be cited independently in the future. For instructions see: https://journals.plos.org/plosone/s/submission-guidelines#loc-laboratory-protocols. Additionally, PLOS ONE offers an option for publishing peer-reviewed Lab Protocol articles, which describe protocols hosted on protocols.io. Read more information on sharing protocols at https://plos.org/protocols?utm_medium=editorial-email&utm_source=authorletters&utm_campaign=protocols.

We look forward to receiving your revised manuscript.

Kind regards,

Emmanuel O Adewuyi, BPharm, MPH, PhD

Academic Editor

PLOS One

**Journal Requirements:**

If the reviewer comments include a recommendation to cite specific previously published works, please review and evaluate these publications to determine whether they are relevant and should be cited. There is no requirement to cite these works unless the editor has indicated otherwise.

**Additional Editor Comments:**

Abstract

- Use significant only in terms of a statistical test. Extend this correction to all relevant sections of the manuscript

- Study aims to assess ‘determinants’, use ‘factors associated with’ instead. Extend this correction to all relevant sections of the manuscript

- Define TR at first mention

- In the results section, the confidence intervals are consistently narrow and exclude the null despite effect sizes being close to 1.0, raising concerns about underestimated standard errors. It is unclear whether the complex survey design (clustering and stratification) was fully accounted for, particularly within the multilevel AFT framework. I see that the authors claimed this was done in the full methods section. What then explains these near-zero estimates? The authors should clarify their variance estimation approach and provide sensitivity analyses (e.g., robust or survey-adjusted standard errors). Additionally, the interpretation should better reflect the modest magnitude of effects.

- The term ‘intergenerational norms’ is unclear and not operationally defined. It is not evident how this construct was measured or derived from the data.
- The study uses data from Lesotho, but no authors appear to have local affiliations. The authors should clarify any local collaboration or, if none, acknowledge this as a limitation and consider a brief reflexivity statement to address potential contextual and interpretive implications (https://gh.bmj.com/content/9/1/e014743).

Background

- The background provides useful context and statistics, but is repetitive and occasionally unclear. Key concepts, such as “time to first marriage” versus “age at first marriage,” could be stated more precisely, and multiple statistics could be summarised for readability. Some terminology (e.g., “limited opportunity structures”) is vague and could be clarified. The rationale for multilevel survival analysis and SDG 5.3 references could be tightened, and citation placement should be checked for consistency. There is a need for improved clarity, flow, and conciseness.

- The authors justify their study by noting a lack of multilevel analysis on first marriage in Lesotho. It would strengthen the rationale to clearly summarise what previous studies have done, their key findings, and how this study will add value to the existing literature. What will your method contribute or find that others did not? The authors should also briefly define what they mean by “multilevel analysis,” explain how it differs from prior approaches, and specify which methodological limitations of earlier studies they aim to address. That is, let your work be written for a broad audience, since this is not a method-focused journal.

- The study applies multilevel survival analysis to cross-sectional DHS data. The authors should briefly clarify how survival methods are valid in this context, given that the data are retrospective and not longitudinal,

- This background section would benefit from language editing

Methods

- Authors mentioned that ‘…survey included 6,413 women between the ages of 15 and 49’. It is unclear whether the 6,413 women represent the total survey sample or a subset included in this analysis. The authors should clarify the sample selection process, including any inclusion/exclusion criteria, handling of missing data, and whether survey weights were applied, to ensure transparency and reproducibility.

Results

- The results raise several concerns. Many 95% confidence intervals are extremely narrow, suggesting possible underestimation of standard errors or insufficient accounting for the survey design.
- The distinction between observed medians and survival-based medians, including the handling of right-censoring, should be clearly explained.
- Covariates such as education, employment, and wealth are measured at survey time, potentially after marriage, raising concerns about reverse causality.
- Some statistically significant effects are very close to 1.0, and their practical significance should be interpreted cautiously.
- While random effects and ICC are reported, their implications and the reporting of sample sizes and censoring across categories should be clarified.

Discussion

- The Discussion section is generally comprehensive but would benefit from greater clarity and precision. Several associations are interpreted too strongly, sometimes implying causality despite the cross‑sectional design and the fact that key covariates were measured after marriage.
- Effect sizes that are statistically significant but very small should be discussed more cautiously.
- The distinction between observed and survival‑based medians needs clearer integration, as subgroup medians often differ from the overall survival estimate.
- While the discussion references relevant literature, deeper analytical comparison would strengthen the narrative. \
- The implications of the reported ICC and cluster-level variance should be elaborated to highlight why community-level differences matter.
- Policy recommendations should be softened to avoid causal inference.
- Finally, the section could be streamlined to reduce redundancy and sharpen its focus on the study's most novel contributions.

[Note: HTML markup is below. Please do not edit.]

Reviewers' comments:

Reviewer's Responses to Questions

**Comments to the Author**

1. If the authors have adequately addressed your comments raised in a previous round of review and you feel that this manuscript is now acceptable for publication, you may indicate that here to bypass the “Comments to the Author” section, enter your conflict of interest statement in the “Confidential to Editor” section, and submit your "Accept" recommendation.

Reviewer #2: All comments have been addressed

Reviewer #3: All comments have been addressed

**********

2. Is the manuscript technically sound, and do the data support the conclusions?

The manuscript must describe a technically sound piece of scientific research with data that supports the conclusions. Experiments must have been conducted rigorously, with appropriate controls, replication, and sample sizes. The conclusions must be drawn appropriately based on the data presented.

Reviewer #2: Yes

Reviewer #3: Yes

**********

3. Has the statistical analysis been performed appropriately and rigorously?

Reviewer #2: Yes

Reviewer #3: Yes

**********

4. Have the authors made all data underlying the findings in their manuscript fully available?

The PLOS Data policy requires authors to make all data underlying the findings described in their manuscript fully available without restriction, with rare exception (please refer to the Data Availability Statement in the manuscript PDF file). The data should be provided as part of the manuscript or its supporting information, or deposited to a public repository. For example, in addition to summary statistics, the data points behind means, medians and variance measures should be available. If there are restrictions on publicly sharing data—e.g. participant privacy or use of data from a third party—those must be specified.

Reviewer #2: Yes

Reviewer #3: Yes

**********

5. Is the manuscript presented in an intelligible fashion and written in standard English?

PLOS ONE does not copyedit accepted manuscripts, so the language in submitted articles must be clear, correct, and unambiguous. Any typographical or grammatical errors should be corrected at revision, so please note any specific errors here.

Reviewer #2: Yes

Reviewer #3: Yes

**********

6. Review Comments to the Author

Please use the space provided to explain your answers to the questions above. You may also include additional comments for the author, including concerns about dual publication, research ethics, or publication ethics. (Please upload your review as an attachment if it exceeds 20,000 characters)

Reviewer #2: Overall, the study is methodologically sound and clearly written. The analytical approach is appropriate for the research question, the data are handled correctly, and the conclusions are supported by the results. The manuscript does not make unwarranted claims of novelty and adheres to principles of transparency and reproducibility.

However, I recommend the following minor revisions, which are largely editorial and clarificatory in nature.

1. If feasible, reporting formal model selection criteria (e.g., AIC or BIC) for the compared parametric models could further strengthen the justification for model choice, although this is not essential for publication.

2. A brief expansion explicitly linking findings to existing educational or rural development policy frameworks in Lesotho could further enhance the discussion, but this is optional.

3. Some long sentences could be shortened to improve readability.

4. Figure captions could explicitly state whether confidence intervals are omitted for clarity.

5. A final language polishing at copy-editing stage is recommended.

Finally, The recommended revisions are minor and largely editorial, and addressing them would further improve clarity and presentation without requiring substantial reanalysis.

Reviewer #3: The manuscript presents a relevant subject for Sub-Saharan Africa. The methods have been well elaborated and results discussed. My previous comments have also been properly addressed.

**********

7. PLOS authors have the option to publish the peer review history of their article (what does this mean?). If published, this will include your full peer review and any attached files.

If you choose “no”, your identity will remain anonymous but your review may still be made public.

**Do you want your identity to be public for this peer review?**  For information about this choice, including consent withdrawal, please see our Privacy Policy.

Reviewer #2: **Yes:** Lawan Adamu

Reviewer #3: **Yes:** Oluwasanmi A. Adedokun

**********

[NOTE: If reviewer comments were submitted as an attachment file, they will be attached to this email and accessible via the submission site. Please log into your account, locate the manuscript record, and check for the action link "View Attachments". If this link does not appear, there are no attachment files.]

To ensure your figures meet our technical requirements, please review our figure guidelines: https://journals.plos.org/plosone/s/figures

You may also use PLOS’s free figure tool, NAAS, to help you prepare publication quality figures: https://journals.plos.org/plosone/s/figures#loc-tools-for-figure-preparation.

NAAS will assess whether your figures meet our technical requirements by comparing each figure against our figure specifications.

# Author comment: Author response to Decision Letter 2

**DOI:** 10.1371/journal.pone.0354034.r004

19 Apr 2026

We would like to thank the editor and the reviewers for their thoughtful and constructive feedback on our manuscript, “Age at first marriage among women in Lesotho: Multilevel survival analysis- insight from 2023-24 demographic and health survey data.”

We have carefully considered all comments and suggestions. Below, we provide a detailed point-by-point response to each editor’s and reviewer comment. Changes made to the manuscript are highlighted in tracked changes manuscript. We hope that these revisions adequately address the editors’ and reviewer concerns and improve the clarity, rigor, and overall quality of the manuscript.

Additional Editor Comments:

Abstract

• Use significant only in terms of a statistical test. Extend this correction to all relevant sections of the manuscript

Response: We thank the editor for this important clarification regarding the precise use of the term "significant." We agree that the word "significant" should be reserved exclusively for reporting statistical significance (p-values), while descriptive language should be used elsewhere. Location in revised manuscript: Abstract [Result section, lines 38-45]; Results, lines [292-294]; Discussion, lines [347; 353-355; 367-368; 386]; Conclusion, lines [449].

• Study aims to assess ‘determinants’, use ‘factors associated with’ instead. Extend this correction to all relevant sections of the manuscript

Response: We acknowledge the editor's point that the term "determinants" implies causal relationships that cannot be established from cross-sectional observational data. We agree that "factors associated with" is more appropriate given our study design. We have replaced all instances of "determinants" with "factors associated with" throughout the manuscript

• Define TR at first mention

Response: We thank the editor for this important clarification. The abbreviation "TR" (Time Ratio) was previously defined only in the Methods section, but it appears first in the Abstract. This has been corrected.

• In the results section, the confidence intervals are consistently narrow and exclude the null despite effect sizes being close to 1.0, raising concerns about underestimated standard errors. It is unclear whether the complex survey design (clustering and stratification) was fully accounted for, particularly within the multilevel AFT framework. I see that the authors claimed this was done in the full methods section. What then explains these near-zero estimates? The authors should clarify their variance estimation approach and provide sensitivity analyses (e.g., robust or survey-adjusted standard errors). Additionally, the interpretation should better reflect the modest magnitude of effects.

Response: We thank the editor for this careful and insightful methodological critique. We acknowledge the concern regarding the narrow confidence intervals and modest effect sizes (time ratios close to 1.0) observed in our results. Below, we provide a point-by-point response and have revised the manuscript accordingly.

Response Point 1: Why are confidence intervals narrow despite effect sizes close to 1.0?

The narrow confidence intervals observed in our analysis are primarily attributable to three factors, none of which indicate methodological error:

• Large sample size and high event count: Our analysis included 6,413 women, of whom 3,127 (48.76%) experienced the event of interest (first marriage). With over 3,000 events, the study is extremely well-powered to detect even small effect sizes. In large samples, standard errors are inherently smaller, leading to narrower confidence intervals. This is a statistical feature of well-powered studies, not a flaw.

• Efficient model specification: The multilevel log-logistic accelerated failure time (AFT) model is appropriately specified for the data structure. The inclusion of cluster-level random effects (ICC = 0.1461) accounts for community-level heterogeneity, which improves precision by partitioning variance correctly.

• Large number of clusters (enumeration areas): The 2023-24 LDHS sampled 400 enumeration areas (EAs) with approximately 16 women per EA. A large number of clusters provides stable estimates of cluster-level variance and contributes to precise standard errors.

Response Point 2: Was complex survey design (clustering and stratification) fully accounted for?

• Yes, the complex survey design was fully accounted for. We provide the following clarification:

• In the Methods section: "To ensure robust statistical estimates, we applied survey weights incorporating sampling weights, primary sampling units (PSUs), and strata variables. This weighting procedure was implemented to correct for sampling design effects and maintain population representativeness in all analyses. All statistical computations, including standard error estimation, accounted for the complex survey design."

Response Point 3: Clarification of Variance Estimation Approach

• We have revised the Methods section to provide a detailed and transparent description of our variance estimation approach. Specifically, we have added a new paragraph in the "Data management and statistical analysis" subsection that clearly outlines how the complex survey design of the 2023-24 LDHS was accounted for within our multilevel AFT framework.

• As described in the revised Methods section, the 2023-24 LDHS used a two-stage stratified cluster sampling design. To account for this complexity, we implemented several procedures: (1) individual sampling weights (v005) were applied to adjust for unequal probability of selection and non-response; (2) the cluster identification variable (v021) was specified as the primary sampling unit (PSU), treating each enumeration area as a PSU; (3) stratification variables using district and urban/peri-urban/rural classification (v023) were included in all analyses; (4) a two-level hierarchical structure was specified with women nested within enumeration areas, where cluster-level random effects capture unobserved heterogeneity across communities; (5) standard errors were estimated using the Taylor linearization method, implemented via the svy prefix in STATA, which is the recommended approach for DHS data as it accounts for clustering, stratification, and weighting simultaneously; and (6) variance components were estimated using restricted maximum likelihood (REML), and the intraclass correlation coefficient (ICC) was calculated to quantify the proportion of total variance attributable to differences between clusters.

• We believe this clarification now provides readers with a complete understanding of how the complex survey design was incorporated into our analysis.

Response Point 4: Sensitivity Analyses (Robust and Survey-Adjusted Standard Errors)

• In response to the editor's request, we have conducted two sensitivity analyses to assess the robustness of our standard errors. These analyses are now presented in the Results section under a new subsection titled "Sensitivity analysis: Comparison of standard error estimation methods," with the full results displayed in Table 5.

• Specifically, we compared three different standard error estimation methods for all statistically significant covariates:

1. Primary model (model-based): Standard errors estimated using Taylor linearization accounting for clustering, stratification, and sampling weights within the multilevel log-logistic AFT framework.

2. Cluster-robust standard errors: Huber-White sandwich estimator clustered at the enumeration area level (400 clusters). This approach makes no distributional assumptions about the random effects structure and provides a conservative test of statistical significance.

3. Bootstrap standard errors: Nonparametric bootstrap with 500 replications, resampling at the cluster level to preserve the survey design.

• Results from sensitivity analyses: “As shown in Table 5, confidence intervals were nearly identical across all three methods. The maximum difference in confidence interval bounds between the primary model and cluster-robust estimates was 0.005 (for higher education: primary lower bound 1.032 vs. cluster-robust lower bound 1.028). The maximum difference for bootstrap estimates was 0.002. All covariates that were statistically significant in the primary model (p < 0.05) remained significant in both sensitivity analyses, with confidence intervals excluding the null value of 1.0. These results confirm that the narrow confidence intervals observed in the primary analysis reflect the large sample size (n = 6,413) and high event count (3,127 events), not underestimated standard errors. The consistency of results across three different standard error estimation methods further confirms the robustness of our findings.”

• The term ‘intergenerational norms’ is unclear and not operationally defined. It is not evident how this construct was measured or derived from the data.

Response: We thank the editor for identifying this ambiguity. We agree that the term "intergenerational norms" was unclear and was not operationally defined or directly measured from our data. This was an oversight in our writing. We have now removed this term from the Conclusions section. The revised text reads: "The findings indicate that education, economic empowerment, and urban residence are associated with a later age at first marriage, while rural livelihoods remain influential factors in marital timing."

• The study uses data from Lesotho, but no authors appear to have local affiliations. The authors should clarify any local collaboration or, if none, acknowledge this as a limitation and consider a brief reflexivity statement to address potential contextual and interpretive implications (https://gh.bmj.com/content/9/1/e014743).

Response: We thank the editor for raising this important ethical and methodological point regarding positionality and local collaboration in global health research. We acknowledge the editor's valid concern. None of the authors have personal or professional affiliations with Lesotho-based organizations or institutions. This is a limitation of our study. To address this transparently, we have added a reflexivity statement to the Discussion section (as a new subsection before "Strengths and limitations") and have acknowledged the absence of local collaborators as a limitation.

Background

• The background provides useful context and statistics, but is repetitive and occasionally unclear. Key concepts, such as “time to first marriage” versus “age at first marriage,” could be stated more precisely, and multiple statistics could be sum marised for readability. Some terminology (e.g., “limited opportunity structures”) is vague and could be clarified. The rationale for multilevel survival analysis and SDG 5.3 references could be tightened, and citation placement should be checked for consistency. There is a need for improved clarity, flow, and conciseness.

Response: We thank the editor for these constructive suggestions. We have thoroughly revised the Background section to address all concerns:

• Precision of key concepts: We have clarified the distinction between "time to first marriage" and "age at first marriage" in a single concise sentence.

• Summarization of statistics: Multiple statistics have been condensed and summarized for improved readability.

• Vague terminology: The phrase "limited opportunity structures" has been replaced with "limited access to education and economic opportunities."

• Rationale for multilevel analysis: The justification for using multilevel survival analysis has been tightened and made more direct.

• SDG 5.3 references: Redundant mentions have been consolidated into a single clear statement.

• Citation placement: All citations have been reviewed and standardized for consistency.

• The authors justify their study by noting a lack of multilevel analysis on first marriage in Lesotho. It would strengthen the rationale to clearly summarise what previous studies have done, their key findings, and how this study will add value to the existing literature. What will your method contribute or find that others did not? The authors should also briefly define what they mean by “multilevel analysis,” explain how it differs from prior approaches, and specify which methodological limitations of earlier studies they aim to address. That is, let your work be written for a broad audience, since this is not a method-focused journal.

Response: We thank the editor for this valuable suggestion to strengthen our rationale and make our methods accessible to a broad audience. We have revised the Background section in the revised manuscript.

• The study applies multilevel survival analysis to cross-sectional DHS data. The authors should briefly clarify how survival methods are valid in this context, given that the data are retrospective and not longitudinal,

Response: We thank the editor for this important methodological clarification. We agree that clarifying the validity of survival analysis with cross-sectional DHS data is essential. And we have revised in the revised manuscript.

• This background section would benefit from language editing

Response: We thank the editor for this constructive suggestion. We have carefully reviewed the entire manuscript and engaged a professional language editing service to improve grammar, clarity, and flow. The Background section has been substantially revised to eliminate repetition, clarify vague terminology, and improve readability. All changes are highlighted in the revised manuscript.

Methods

• Authors mentioned that ‘…survey included 6,413 women between the ages of 15 and 49’. It is unclear whether the 6,413 women represent the total survey sample or a subset included in this analysis. The authors should clarify the sample selection process, including any inclusion/exclusion criteria, handling of missing data, and whether survey weights were applied, to ensure transparency and reproducibility.

Response: We thank the editor. We have clarified that the 6,413 women represent the total LDHS sample, all included in analysis. Inclusion criteria (all women aged 15–49) and handling of missing data (<5%; complete-case analysis with multiple imputation sensitivity) have been added. Survey weights (v005) were applied in all analyses. Revisions are highlighted.

Results

• The results raise several concerns. Many 95% confidence intervals are extremely narrow, suggesting possible underestimation of standard errors or insufficient accounting for the survey design.

Response: We thank the editor for this concern. We have conducted sensitivity analyses comparing model-based, cluster-robust, and bootstrap standard errors (Table 5), confirming robustness. Narrow confidence intervals reflect the large sample size (n=6,413) and high event count (3,127), not underestimated errors. The survey design was fully accounted for using weights, PSUs, stratification, and Taylor linearization. All revisions are highlighted.

• The distinction between observed medians and survival-based medians, including the handling of right-censoring, should be clearly explained.

Response: We thank the editor for this clarification. We have revised the Results section to clearly distinguish between observed medians (calculated only from women who have married) and survival-based medians (estimated from the Kaplan-Meier method accounting for right-censoring). We explain that observed medians are lower because they exclude censored women, while survival-based medians are the preferred estimates for population inference. The revised text is highlighted in the manuscript.

• Covariates such as education, employment, and wealth are measured at survey time, potentially after marriage, raising concerns about reverse causality.

Response: We thank the editor for this important methodological concern. We acknowledge this limitation and have added a clear statement in the Discussion section (Strengths and limitations) addressing the potential for reverse causality, explaining that covariates measured at survey time may not reflect pre-marriage status. We also note that DHS data do not allow temporal ordering for time-varying covariates and recommend future longitudinal studies.

• Some statistically significant effects are very close to 1.0, and their practical significan

Attachment Submitted filename: Response_to_Reviewers_auresp_2.docx

# Peer review: Decision Letter 2

**DOI:** 10.1371/journal.pone.0354034.r005

**Authors:** Jianhong Zhou

11 May 2026

<div>PONE-D-25-41860R2

Age at first marriage among women in Lesotho: Multilevel survival analysis- insight from 2023-24 demographic and health survey data

PLOS One

Dear Dr. Shibeshi,

Thank you for submitting your manuscript to PLOS ONE. After careful consideration, we feel that it has merit but does not fully meet PLOS ONE’s publication criteria as it currently stands. Therefore, we invite you to submit a revised version of the manuscript that addresses the points raised during the review process.

Please submit your revised manuscript by Jun 22 2026 11:59PM. If you will need more time than this to complete your revisions, please reply to this message or contact the journal office at plosone@plos.org. When you're ready to submit your revision, log on to https://www.editorialmanager.com/pone/ and select the 'Submissions Needing Revision' folder to locate your manuscript file.

Please include the following items when submitting your revised manuscript:

- A letter that responds to each point raised by the academic editor and reviewer(s). You should upload this letter as a separate file labeled 'Response to Reviewers'.
- A marked-up copy of your manuscript that highlights changes made to the original version. You should upload this as a separate file labeled 'Revised Manuscript with Track Changes'.
- An unmarked version of your revised paper without tracked changes. You should upload this as a separate file labeled 'Manuscript'.

If you would like to make changes to your financial disclosure, please include your updated statement in your cover letter. Guidelines for resubmitting your figure files are available below the reviewer comments at the end of this letter.

If applicable, we recommend that you deposit your laboratory protocols in protocols.io to enhance the reproducibility of your results. Protocols.io assigns your protocol its own identifier (DOI) so that it can be cited independently in the future. For instructions see: https://journals.plos.org/plosone/s/submission-guidelines#loc-laboratory-protocols. Additionally, PLOS ONE offers an option for publishing peer-reviewed Lab Protocol articles, which describe protocols hosted on protocols.io. Read more information on sharing protocols at https://plos.org/protocols?utm_medium=editorial-email&utm_source=authorletters&utm_campaign=protocols.

As the corresponding author, your ORCID iD is verified in the submission system and will appear in the published article. PLOS supports the use of ORCID, and we encourage all coauthors to register for an ORCID iD and use it as well. Please encourage your coauthors to verify their ORCID iD within the submission system before final acceptance, as unverified ORCID iDs will not appear in the published article. *Only*  the individual author can complete the verification step; PLOS staff *cannot*  verify ORCID iDs on behalf of authors.

We look forward to receiving your revised manuscript.

Kind regards,

Jianhong Zhou

Staff Editor

PLOS One

Journal Requirements:

If the reviewer comments include a recommendation to cite specific previously published works, please review and evaluate these publications to determine whether they are relevant and should be cited. There is no requirement to cite these works unless the editor has indicated otherwise.

Please review your reference list to ensure that it is complete and correct. If you have cited papers that have been retracted, please include the rationale for doing so in the manuscript text, or remove these references and replace them with relevant current references. Any changes to the reference list should be mentioned in the rebuttal letter that accompanies your revised manuscript. If you need to cite a retracted article, indicate the article’s retracted status in the References list and also include a citation and full reference for the retraction notice.

[Note: HTML markup is below. Please do not edit.]

Reviewers' comments:

Reviewer's Responses to Questions

**Comments to the Author**

1. If the authors have adequately addressed your comments raised in a previous round of review and you feel that this manuscript is now acceptable for publication, you may indicate that here to bypass the “Comments to the Author” section, enter your conflict of interest statement in the “Confidential to Editor” section, and submit your "Accept" recommendation.

Reviewer #2: All comments have been addressed

**********

2. Is the manuscript technically sound, and do the data support the conclusions?

The manuscript must describe a technically sound piece of scientific research with data that supports the conclusions. Experiments must have been conducted rigorously, with appropriate controls, replication, and sample sizes. The conclusions must be drawn appropriately based on the data presented.

Reviewer #2: Yes

**********

3. Has the statistical analysis been performed appropriately and rigorously?

Reviewer #2: Yes

**********

4. Have the authors made all data underlying the findings in their manuscript fully available?

The PLOS Data policy requires authors to make all data underlying the findings described in their manuscript fully available without restriction, with rare exception (please refer to the Data Availability Statement in the manuscript PDF file). The data should be provided as part of the manuscript or its supporting information, or deposited to a public repository. For example, in addition to summary statistics, the data points behind means, medians and variance measures should be available. If there are restrictions on publicly sharing data—e.g. participant privacy or use of data from a third party—those must be specified.

Reviewer #2: Yes

**********

5. Is the manuscript presented in an intelligible fashion and written in standard English?

PLOS ONE does not copyedit accepted manuscripts, so the language in submitted articles must be clear, correct, and unambiguous. Any typographical or grammatical errors should be corrected at revision, so please note any specific errors here.

Reviewer #2: Yes

**********

6. Review Comments to the Author

Please use the space provided to explain your answers to the questions above. You may also include additional comments for the author, including concerns about dual publication, research ethics, or publication ethics. (Please upload your review as an attachment if it exceeds 20,000 characters)

Reviewer #2: Generally, this revised version represents a clear and substantial improvement over the previous submission. Your responses to the editor—particularly regarding standard error estimation and sensitivity analyses—are thorough and commendable.

That said, the following few refinements will further strengthen the manuscript.

1. Despite improvements, a few interpretations still lean toward causality. For example: Statements implying that education or employment delays marriage should consistently reflect association, not effect.

Hence, you should ensure consistent phrasing such as: “associated with later age at first marriage” and Avoid language suggesting direct influence or impact

2. You acknowledge that variables like education, wealth, and employment are measured at survey time. However, this limitation should be: More prominently emphasized and briefly reiterated when interpreting key findings

I Suggest you should add the following short reinforcing sentence in Discussion:

“These associations should be interpreted cautiously, as key covariates were measured after the occurrence of marriage for many participants.”

3. You correctly note that TR values are close to 1.0. However, this caution should be made more prominent, not only in one paragraph

I Recommend you add the following sentence in the Results section acknowledging small effect sizes:

“Although statistically significant, the magnitude of these effects is modest.”

4. Some sections remain unnecessarily long, particularly:

• Background (repetition of DHS context and early marriage impacts)

• Methods (especially multilevel explanations)

• Discussion (repetition of findings)

I Recommend you should:

• Reduce redundancy by ~10–15% overall

• Focus on precision rather than repetition

5. You report ICC = 0.1461, but interpretation could still be clearer if you explicitly state that: “Approximately 14.6% of the variation in age at first marriage is attributable to differences between communities.” This improves accessibility for non-technical readers.

6. The manuscript states: “N/A” initially, then later provides explanation, I recommend you Remove “N/A”, and keep only the full DHS-based ethical explanation for consistency

7. For Language and Grammar, the manuscript has improved, but minor issues persist: Examples:

• “We conducted a retrospective time-to-event data…” (grammatical inconsistency)

• Occasional long sentences affecting readability

To address this, I think a Final professional copy-editing is still needed

**********

7. PLOS authors have the option to publish the peer review history of their article (what does this mean?). If published, this will include your full peer review and any attached files.

If you choose “no”, your identity will remain anonymous but your review may still be made public.

**Do you want your identity to be public for this peer review?**  For information about this choice, including consent withdrawal, please see our Privacy Policy.

Reviewer #2: **Yes:** Lawan Adamu

**********

[NOTE: If reviewer comments were submitted as an attachment file, they will be attached to this email and accessible via the submission site. Please log into your account, locate the manuscript record, and check for the action link "View Attachments". If this link does not appear, there are no attachment files.]

To ensure your figures meet our technical requirements, please review our figure guidelines: https://journals.plos.org/plosone/s/figures

You may also use PLOS’s free figure tool, NAAS, to help you prepare publication quality figures: https://journals.plos.org/plosone/s/figures#loc-tools-for-figure-preparation.

NAAS will assess whether your figures meet our technical requirements by comparing each figure against our figure specifications.

# Author comment: Author response to Decision Letter 3

**DOI:** 10.1371/journal.pone.0354034.r006

31 May 2026

We would like to thank the editor and the reviewers for their thoughtful and constructive feedback on our manuscript, “Age at first marriage among women in Lesotho: Multilevel survival analysis- insight from 2023-24 demographic and health survey data.”

We have carefully considered all comments and suggestions. Below, we provide a detailed point-by-point response to each reviewer comment. Changes made to the manuscript are highlighted in tracked changes manuscript. We hope that these revisions adequately address the reviewer concerns and improve the clarity, rigor, and overall quality of the manuscript.

Review Comments to the Author:

Reviewer #2: Generally, this revised version represents a clear and substantial improvement over the previous submission. Your responses to the editor—particularly regarding standard error estimation and sensitivity analyses—are thorough and commendable.

That said, the following few refinements will further strengthen the manuscript.

1. Despite improvements, a few interpretations still lean toward causality. For example: Statements implying that education or employment delays marriage should consistently reflect association, not effect.

Hence, you should ensure consistent phrasing such as: “associated with later age at first marriage” and Avoid language suggesting direct influence or impact.

Response: We sincerely thank the reviewer for the thoughtful suggestions to further refine the language and interpretation. We have systematically revised the entire manuscript to replace any language that could imply causality (e.g., ‘delays,’ ‘accelerates,’ ‘leads to,’ ‘influence,’ ‘effect,’ ‘impact’) with strictly associative phrasing such as ‘associated with,’ ‘correlated with,’ or ‘related to.’ All instances in the Abstract, Results, Discussion, and Conclusions have been amended as detailed in the tracked-changes version. No causal interpretations remain.

2. You acknowledge that variables like education, wealth, and employment are measured at survey time. However, this limitation should be: More prominently emphasized and briefly reiterated when interpreting key findings

I Suggest you should add the following short reinforcing sentence in Discussion:

“These associations should be interpreted cautiously, as key covariates were measured after the occurrence of marriage for many participants.”

Response: We thank the reviewer for this important methodological observation. We have now prominently emphasized this limitation in the Strengths and limitations subsection and have added the reviewer's suggested sentence verbatim in the Discussion section, immediately following the interpretation of key findings.

3. You correctly note that TR values are close to 1.0. However, this caution should be made more prominent, not only in one paragraph

I Recommend you add the following sentence in the Results section acknowledging small effect sizes:

“Although statistically significant, the magnitude of these effects is modest.”

Response: We thank the reviewer for this important suggestion. We have now added the reviewer's suggested sentence verbatim in the Results section, immediately following the presentation of the key statistically significant findings in Table 4. Additionally, we have reinforced this caution in the Abstract and Conclusions sections to ensure readers encounter this important qualification at multiple points in the manuscript.

4. Some sections remain unnecessarily long, particularly:

• Background (repetition of DHS context and early marriage impacts)

• Methods (especially multilevel explanations)

• Discussion (repetition of findings)

I Recommend you should:

• Reduce redundancy by ~10–15% overall

• Focus on precision rather than repetition

Response: We thank the reviewer for this constructive feedback. We have systematically revised the manuscript to reduce redundancy and improve conciseness, focusing specifically on the three sections noted. Background section, methods section, and discussion section.

5. You report ICC = 0.1461, but interpretation could still be clearer if you explicitly state that: “Approximately 14.6% of the variation in age at first marriage is attributable to differences between communities.” This improves accessibility for non-technical readers.

Response: We thank the reviewer for this helpful suggestion to improve accessibility for non-technical readers. We have now revised the interpretation of the ICC throughout the manuscript to explicitly state the percentage of variation attributable to community-level differences.

6. The manuscript states: “N/A” initially, then later provides explanation, I recommend you Remove “N/A”, and keep only the full DHS-based ethical explanation for consistency

Response: Thank you for this valuable observation. We agree that the inclusion of “N/A” in the ethical considerations section was inconsistent with the detailed ethical explanation provided later in the manuscript. Accordingly, we have removed the “N/A” statement and retained a comprehensive ethical considerations section describing the DHS data access procedures, anonymization of participant information, informed consent process, and ethical safeguards implemented by the DHS Program.

7. For Language and Grammar, the manuscript has improved, but minor issues persist: Examples:

• “We conducted a retrospective time-to-event data…” (grammatical inconsistency)

• Occasional long sentences affecting readability

To address this, I think a Final professional copy-editing is still needed

Response: We thank the reviewer for identifying the remaining minor language issues. Specifically:

1. The grammatical inconsistency "We conducted a retrospective time-to-event data..." has been corrected to "We analyzed retrospective time-to-event data..."

2. Long sentences affecting readability have been shortened or broken down in five locations: the Background (paragraph 4), Methods (data management and model justification subsections), and Discussion (modest associations paragraph).

3. We agree that professional copy-editing is warranted. The manuscript will undergo a final professional copy-edit prior to submission to ensure the highest standards of language clarity and grammatical correctness.

All changes have been made in the tracked-changes version of the manuscript.

We extend our sincere gratitude to the editor and reviewers for their thoughtful and constructive feedback. We believe these revisions have substantially improved the manuscript's scientific rigor, clarity, and compliance with publishing standards. Thank you once again for your valuable input.

Attachment Submitted filename: Response_to_Reviewers_auresp_3.docx

# Peer review: Decision Letter 3

**DOI:** 10.1371/journal.pone.0354034.r007

**Authors:** José Antonio Ortega

14 Jun 2026

PONE-D-25-41860R3

Age at first marriage among women in Lesotho: Multilevel survival analysis- insight from 2023-24 demographic and health survey data

PLOS One

Dear Dr. Shibeshi,

Thank you for submitting your manuscript to PLOS ONE. After careful consideration, we feel that it has merit but does not fully meet PLOS ONE’s publication criteria as it currently stands. Therefore, we invite you to submit a revised version of the manuscript that addresses the points raised during the review process.

The previous academic editor was not available and I have taken over. The last reviewer asking for revisions, who insisted on the reverse causality aspects, recommends acceptance.

I understand that, to the extent possible, the editorial service should not revise previous decisions without a good reason, but I believe there are very good reasons why this manuscript needs to be revised.

Let me first point to the internal contradictions: Reference 17, on adolescent fertility, is the only reference provided for the choice of covariates. But this is very mistaken: adolescent fertility is generally studied with women currently exposed to the risk. Age at first marriage with women 15-49 analysis an event that in many cases happened 20 or 30 years ago. This makes interpretation radically different.

There are also problems in the description of the variables: "Head education Educational level", this is the minimum educational level a woman achieved ...

For the reason mentioned first, it is not enough to pay lip service to reverse causality. One needs to avoid unnecessary use of variables that are of a short-nature or that have a confusing relationship to the variable under study.

In particular, in order to qualify for publication three variables that have clear problem should be removed from the model: mass media exposure, head occupation and head education level.

The reason for the first is that current mass media exposure cannot be or remotely be a cause of a marriage that might have happened decades ago. The reason to remove the head variables is that they do not capture any meaningful association given that the head variables will be mostly those of the father (or similar figure) for currently unmarried women and the spouse in currenly married (or the women in no longer married women). This creates a particular conflict in the kind of analysis used, survival analysis, since the relationship to the ego of the measured variable is dependent on the transition having occurred. I do not believe that any association so poorly measured can be of any value for policy.

On the other respect, one more variable should be added to capture changes over time. It is well known that marriage has been postponing in most countries of the world in the last decades including in Lesotho (see, for instance, doi:10.1007/s11113-013-9301-x), although a recent article points to continuity in the context of Lesotho (https://doi.org/10.1007/s42650-025-00107-6). Note that some of the variables currently included might be capturing changes over cohorts that are not included in the model as a possibility through other variables that change over cohorts and currently appear in the model. You should at least include some variable indicating the birth cohort, probably as a categorical variable for consistence with the current approach, to capture these possible changes over time.

To compensate for the delays in the revision process, the revised manuscript will not be sent to reviewers. I will personally evaluate whether these changes have been introduced.

Please submit your revised manuscript by Jul 29 2026 11:59PM. If you will need more time than this to complete your revisions, please reply to this message or contact the journal office at plosone@plos.org. When you're ready to submit your revision, log on to https://www.editorialmanager.com/pone/ and select the 'Submissions Needing Revision' folder to locate your manuscript file.

Please include the following items when submitting your revised manuscript:

- A letter that responds to each point raised by the academic editor and reviewer(s). You should upload this letter as a separate file labeled 'Response to Reviewers'.
- A marked-up copy of your manuscript that highlights changes made to the original version. You should upload this as a separate file labeled 'Revised Manuscript with Track Changes'.
- An unmarked version of your revised paper without tracked changes. You should upload this as a separate file labeled 'Manuscript'.

If you would like to make changes to your financial disclosure, please include your updated statement in your cover letter. Guidelines for resubmitting your figure files are available below the reviewer comments at the end of this letter.

If applicable, we recommend that you deposit your laboratory protocols in protocols.io to enhance the reproducibility of your results. Protocols.io assigns your protocol its own identifier (DOI) so that it can be cited independently in the future. For instructions see: https://journals.plos.org/plosone/s/submission-guidelines#loc-laboratory-protocols. Additionally, PLOS ONE offers an option for publishing peer-reviewed Lab Protocol articles, which describe protocols hosted on protocols.io. Read more information on sharing protocols at https://plos.org/protocols?utm_medium=editorial-email&utm_source=authorletters&utm_campaign=protocols.

As the corresponding author, your ORCID iD is verified in the submission system and will appear in the published article. PLOS supports the use of ORCID, and we encourage all coauthors to register for an ORCID iD and use it as well. Please encourage your coauthors to verify their ORCID iD within the submission system before final acceptance, as unverified ORCID iDs will not appear in the published article. *Only*  the individual author can complete the verification step; PLOS staff *cannot*  verify ORCID iDs on behalf of authors.

We look forward to receiving your revised manuscript.

Kind regards,

José Antonio Ortega, Ph.D.

Academic Editor

PLOS One

Journal Requirements:

If the reviewer comments include a recommendation to cite specific previously published works, please review and evaluate these publications to determine whether they are relevant and should be cited. There is no requirement to cite these works unless the editor has indicated otherwise.

[Note: HTML markup is below. Please do not edit.]

Reviewers' comments:

Reviewer's Responses to Questions

**Comments to the Author**

1. If the authors have adequately addressed your comments raised in a previous round of review and you feel that this manuscript is now acceptable for publication, you may indicate that here to bypass the “Comments to the Author” section, enter your conflict of interest statement in the “Confidential to Editor” section, and submit your "Accept" recommendation.

Reviewer #2: All comments have been addressed

**********

2. Is the manuscript technically sound, and do the data support the conclusions?

The manuscript must describe a technically sound piece of scientific research with data that supports the conclusions. Experiments must have been conducted rigorously, with appropriate controls, replication, and sample sizes. The conclusions must be drawn appropriately based on the data presented.

Reviewer #2: Yes

**********

3. Has the statistical analysis been performed appropriately and rigorously?

Reviewer #2: Yes

**********

4. Have the authors made all data underlying the findings in their manuscript fully available?

The PLOS Data policy requires authors to make all data underlying the findings described in their manuscript fully available without restriction, with rare exception (please refer to the Data Availability Statement in the manuscript PDF file). The data should be provided as part of the manuscript or its supporting information, or deposited to a public repository. For example, in addition to summary statistics, the data points behind means, medians and variance measures should be available. If there are restrictions on publicly sharing data—e.g. participant privacy or use of data from a third party—those must be specified.

Reviewer #2: Yes

**********

5. Is the manuscript presented in an intelligible fashion and written in standard English?

PLOS ONE does not copyedit accepted manuscripts, so the language in submitted articles must be clear, correct, and unambiguous. Any typographical or grammatical errors should be corrected at revision, so please note any specific errors here.

Reviewer #2: Yes

**********

6. Review Comments to the Author

Please use the space provided to explain your answers to the questions above. You may also include additional comments for the author, including concerns about dual publication, research ethics, or publication ethics. (Please upload your review as an attachment if it exceeds 20,000 characters)

Reviewer #2: The manuscript is scientifically sound and methodologically appropriate and suitable for publication

**********

7. PLOS authors have the option to publish the peer review history of their article (what does this mean?). If published, this will include your full peer review and any attached files.

If you choose “no”, your identity will remain anonymous but your review may still be made public.

**Do you want your identity to be public for this peer review?**  For information about this choice, including consent withdrawal, please see our Privacy Policy.

Reviewer #2: **Yes:** Lawan Adamu

**********

[NOTE: If reviewer comments were submitted as an attachment file, they will be attached to this email and accessible via the submission site. Please log into your account, locate the manuscript record, and check for the action link "View Attachments". If this link does not appear, there are no attachment files.]

To ensure your figures meet our technical requirements, please review our figure guidelines: https://journals.plos.org/plosone/s/figures

You may also use PLOS’s free figure tool, NAAS, to help you prepare publication quality figures: https://journals.plos.org/plosone/s/figures#loc-tools-for-figure-preparation.

NAAS will assess whether your figures meet our technical requirements by comparing each figure against our figure specifications.

# Author comment: Author response to Decision Letter 4

**DOI:** 10.1371/journal.pone.0354034.r008

26 Jun 2026

Manuscript Title: Age at first marriage among women in Lesotho: Multilevel survival analysis- insight from 2023-24 demographic and health survey data

Manuscript ID: PONE-D-25-41860R3

Journal: PLOS ONE

Corresponding Author: Abdu Hailu Shibeshi

Date: June 26, 2026.

Dear Dr. José Antonio Ortega,

Thank you for your thoughtful and constructive feedback on our manuscript (PONE-D-25-41860R3). We sincerely appreciate the time and effort you have invested in reviewing our work and providing detailed guidance for revision.

We have carefully addressed all the points raised in your editorial comments and have revised the manuscript accordingly. Below, we provide a point-by-point response to each of your specific concerns. All changes in the manuscript have been marked using track changes for your easy reference.

Response to Academic Editor's Comments

Comment 1: Internal Contradictions - Reference 17

Editor's Comment:

"Reference 17, on adolescent fertility, is the only reference provided for the choice of covariates. But this is very mistaken: adolescent fertility is generally studied with women currently exposed to the risk. Age at first marriage with women 15-49 analysis an event that in many cases happened 20 or 30 years ago. This makes interpretation radically different."

Response: We agree with the editor's observation. We have revised the literature review to include more appropriate references that specifically address age at first marriage rather than adolescent fertility. We have also expanded our justification for covariate selection; drawing from studies that examine marriage timing among women aged 15-49 in sub-Saharan African contexts

Comment 2: Variable Description Issues

Editor's Comment:

"There are also problems in the description of the variables: 'Head education Educational level', this is the minimum educational level a woman achieved..."

Response: We have removed the head education and head occupation variables entirely, as recommended by the editor. Therefore, this description issue is no longer relevant to the manuscript.

Comment 3: Reverse Causality

Editor's Comment:

"For the reason mentioned first, it is not enough to pay lip service to reverse causality. One needs to avoid unnecessary use of variables that are of a short-nature or that have a confusing relationship to the variable under study."

Response: We have taken a more rigorous approach to addressing reverse causality. Specifically, we have removed three variables with clear temporal or measurement issues: mass media exposure, head occupation, and head education level. We have also added a birth cohort variable that is fixed and cannot be affected by marriage, which strengthens causal inference.

Comment 4: Variables to Remove

Editor's Comment:

"In particular, in order to qualify for publication three variables that have clear problem should be removed from the model: mass media exposure, head occupation and head education level. The reason for the first is that current mass media exposure cannot be or remotely be a cause of a marriage that might have happened decades ago. The reason to remove the head variables is that they do not capture any meaningful association given that the head variables will be mostly those of the father (or similar figure) for currently unmarried women and the spouse in currently married (or the women in no longer married women). This creates a particular conflict in the kind of analysis used, survival analysis, since the relationship to the ego of the measured variable is dependent on the transition having occurred. I do not believe that any association so poorly measured can be of any value for policy."

Response: We fully agree with the editor's assessment and have removed all three variables from the manuscript. This decision is now clearly justified in the Methods section.

Comment 5: Variable to Add - Birth Cohort

Editor's Comment:

"One more variable should be added to capture changes over time. It is well known that marriage has been postponing in most countries of the world in the last decades including in Lesotho (see, for instance, doi:10.1007/s11113-013-9301-x), although a recent article points to continuity in the context of Lesotho (https://doi.org/10.1007/s42650-025-00107-6). Note that some of the variables currently included might be capturing changes over cohorts that are not included in the model as a possibility through other variables that change over cohorts and currently appear in the model. You should at least include some variable indicating the birth cohort, probably as a categorical variable for consistence with the current approach, to capture these possible changes over time."

Response: We have added a birth cohort variable to capture secular changes in marriage timing over time. Following the editor's suggestion, we created a categorical variable with 5-year intervals based on the woman's year of birth.

Comment 6: Revised Manuscript Will Not Be Sent to Reviewers

Editor's Comment:

"To compensate for the delays in the revision process, the revised manuscript will not be sent to reviewers. I will personally evaluate whether these changes have been introduced."

Response: We appreciate the editor's willingness to personally evaluate the revised manuscript. We have carefully addressed all the points raised and hope that the revisions meet the editor's expectations.

Attachment Submitted filename: Response_to_Reviewers_auresp_4.docx

# Editor report: Decision Letter 4

**DOI:** 10.1371/journal.pone.0354034.r009

**Authors:** José Antonio Ortega

3 Jul 2026

Age at first marriage among women in Lesotho: Multilevel survival analysis- insight from 2023-24 demographic and health survey data

PONE-D-25-41860R4

Dear Dr. Shibeshi,

We’re pleased to inform you that your manuscript has been judged scientifically suitable for publication and will be formally accepted for publication once it meets all outstanding technical requirements.

Within one week, you’ll receive an e-mail detailing the required amendments. When these have been addressed, you’ll receive a formal acceptance letter and your manuscript will be scheduled for publication.

An invoice will be generated when your article is formally accepted. Please note, if your institution has a publishing partnership with PLOS and your article meets the relevant criteria, all or part of your publication costs will be covered. Please make sure your user information is up-to-date by logging into Editorial Manager at Editorial Manager® and clicking the ‘Update My Information' link at the top of the page. For questions related to billing, please contact billing support.

If your institution or institutions have a press office, please notify them about your upcoming paper to help maximize its impact. If they’ll be preparing press materials, please inform our press team as soon as possible -- no later than 48 hours after receiving the formal acceptance. Your manuscript will remain under strict press embargo until 2 pm Eastern Time on the date of publication. For more information, please contact onepress@plos.org.

Kind regards,

José Antonio Ortega, Ph.D.

Academic Editor

PLOS One

Additional Editor Comments (optional):

The ammendments to the manuscripts have avoided the most problematic issues and the expanded limitations make its limits clearer. The manuscript is apt for publication.

Reviewers' comments:

# Editor report: Acceptance letter

**DOI:** 10.1371/journal.pone.0354034.r010

**Authors:** José Antonio Ortega

PONE-D-25-41860R4

PLOS One

Dear Dr. Shibeshi,

I'm pleased to inform you that your manuscript has been deemed suitable for publication in PLOS One. Congratulations! Your manuscript is now being handed over to our production team.

At this stage, our production department will prepare your paper for publication. This includes ensuring the following:

* All references, tables, and figures are properly cited

* All relevant supporting information is included in the manuscript submission,

* There are no issues that prevent the paper from being properly typeset

You will receive further instructions from the production team, including instructions on how to review your proof when it is ready. Please keep in mind that we are working through a large volume of accepted articles, so please give us a few days to review your paper and let you know the next and final steps.

Lastly, if your institution or institutions have a press office, please let them know about your upcoming paper now to help maximize its impact. If they'll be preparing press materials, please inform our press team within the next 48 hours. Your manuscript will remain under strict press embargo until 2 pm Eastern Time on the date of publication. For more information, please contact onepress@plos.org.

You will receive an invoice from PLOS for your publication fee after your manuscript has reached the completed accept phase. If you receive an email requesting payment before acceptance or for any other service, this may be a phishing scheme. Learn how to identify phishing emails and protect your accounts at https://explore.plos.org/phishing.

If we can help with anything else, please email us at customercare@plos.org.

Thank you for submitting your work to PLOS ONE and supporting open access.

Kind regards,

PLOS ONE Editorial Office Staff

on behalf of

Dr. José Antonio Ortega

Academic Editor

PLOS One
